# Evolution of Cd^2+^ and Cu^+^ binding in *Helix pomatia* metallothioneins

**DOI:** 10.1093/mtomcs/mfad057

**Published:** 2023-09-20

**Authors:** Renato Valsecchi, Christian Baumann, Ardit Lila, Oliver Zerbe

**Affiliations:** Department of Chemistry, University of Zurich, 8057 Zurich, Winterthurerstrasse 190, Switzerland; Department of Chemistry, University of Zurich, 8057 Zurich, Winterthurerstrasse 190, Switzerland; Department of Chemistry, University of Zurich, 8057 Zurich, Winterthurerstrasse 190, Switzerland; Department of Chemistry, University of Zurich, 8057 Zurich, Winterthurerstrasse 190, Switzerland

**Keywords:** NMR, cadmium, copper, conformational exchange, T_2_ relaxation, ancestral proteins

## Abstract

Metallothioneins (MTs) are small proteins present in all kingdoms of life. Their high cysteine content enables them to bind metal ions, such as Zn^2+^, Cd^2+^, and Cu^+^, providing means for detoxification and metal homeostasis. Three MT isoforms with distinct metal binding preferences are present in the Roman Snail *Helix pomatia*. Here, we use nuclear magnetic resonance (NMR) to follow the evolution of Cd^2+^ and Cu^+^ binding from the reconstructed ancestral Stylommatophora MT to the three *H. pomatia* MT (HpMT) isoforms. Information obtained from [^15^N,^1^H]-HSQC spectra and T_2_ relaxation times are combined to describe the conformational stability of the MT-metal complexes. A well-behaved MT-metal complex adopts a unique structure and does not undergo additional conformational exchange. The ancestor to all three HpMTs forms conformationally stable Cd^2+^ complexes and closely resembles the Cd^2+^-specific HpCdMT isoform, suggesting a role in Cd^2+^ detoxification for the ancestral protein. All Cu^+^-MT complexes, including the Cu^+^-specific HpCuMT isoform, undergo a considerable amount of conformational exchange. The unspecific HpCd/CuMT and the Cu^+^-specific HpCuMT isoforms form Cu^+^ complexes with comparable characteristics. It is possible to follow how Cd^2+^ and Cu^+^ binding changed throughout evolution. Interestingly, Cu^+^ binding improved independently in the lineages leading to the unspecific and the Cu^+^-specific HpMT isoforms. C-terminal domains are generally less capable of coordinating the non-cognate metal ion than N-terminal domains, indicating a higher level of specialization of the C-domain. Our findings provide new insights into snail MT evolution, helping to understand the interplay between biological function and structural features toward a comprehensive understanding of metal preference.

## Graphical Abstract

**Graphical Abstract:**
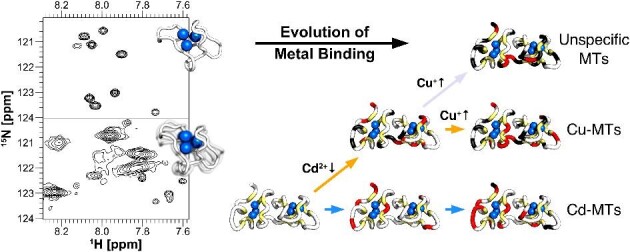
Well-behaved metallothionein-metal complexes adopt unique structures that lack conformational exchange. The conformational stability of these complexes can be probed using NMR. We follow the evolution of metal binding in gastropod metallothioneins by investigating the conformational stability of Cd^2+^ and Cu^+^ complexes formed by extant and ancestral proteins.

## Introduction

Metallothioneins (MTs) are small Cys-rich proteins that coordinate metal ions in metal-thiolate clusters. Amongst the most studied MTs are mammalian MTs, which coordinate metal ions in two clusters, comprising nine (β-cluster) and eleven (α-cluster) cysteine residues, which can bind three and four divalent metal ions, respectively.^1,2^ Although studied since 1957, the biological roles of many MTs remain enigmatic, at least partially because single MTs can have multiple functions.^3^ These functions can even further vary between different life stages of an organism, as is, e.g. the case in the plant *Arabidopsis thaliana*.^4,5^ The situation is different in invertebrate MTs, among which gastropod MTs became the model to study structure–function relationships of MTs. Clear associations to either Cd^2+^ detoxification or Cu^+^ homeostasis are present for a set of gastropod MTs.^6^ However, it remains unclear how metal preferences are determined on a molecular level.

Stylommatophora is an order within Gastropoda that includes land snails and slugs such as the Roman Snail (*Helix pomatia*) or the Great Gray Slug (*Limax maximus*). *Helix pomatia*, like other members of Stylommatophora that belong to the family of Helicidae, possesses three MT isoforms, each with a distinct metal preference. Two isoforms are specific for a single type of metal ion and are purified from *H. pomatia* and the garden snail *Cornu aspersum* mostly in homometallic complexes with their cognate metal ion.^6,7^ One of these isoforms is the Cd^2+^-specific CdMT, which is transcribed in large amounts in digestive tissues upon Cd^2+^ exposure and is thus thought to be responsible for Cd^2+^ detoxification.^7,8^ This MT isoform immobilizes 80–95% of the entire Cd^2+^ pool thereby preventing toxic interactions of Cd^2+^ ions with other cellular components.^6,7^ The second MT isoform is the Cu^+^-specific CuMT, which is constitutively expressed in specialized cells (rhogocytes) and is assumed to be involved in the homeostatic regulation of the cellular Cu^+^-pool to support the correct synthesis of the respiratory pigment hemocyanin.^8–10^

Both MT isoforms give rise to homometallic complexes when expressed in *Escherichia coli* with their cognate metal ion supplemented to the expression medium but result in heterogeneous and sometimes heteronuclear complexes when expressed with the non-cognate metal ion.^8,11^ This led to a classification based on how homogenously MTs bind divalent Zn^2+^/Cd^2+^- and monovalent Cu^+^ ions. The underlying rationale is that, when binding the cognate metal ion species, selective MTs produce a well-folded metal-protein complex with a fixed metal stoichiometry.^11,12^ The binding of non-cognate metal ions on the other hand leads to a heterogeneous ensemble of conformational states, which can accommodate different numbers of metal ions.^11,12^ Recently, this classification system was expanded to distinguish between Cd^2+^- and Zn^2+^-selective MTs as well.^13^ This type of metal-ion preference is termed metal-selectivity to distinguish it from metal-specificity, which refers to the physiological function (such as induction of expression and tissue-specific localization) of a MT.^14^

A third MT isoform was first identified in *C. aspersum* and later in *H. pomatia*, and termed Cd/CuMT because it was purified from snails as heterometallic complexes with Cu^+^ and Cd^2+^.^7,8,15^ A mixture of different metal loadings and stoichiometries are obtained from recombinant expression in *E. coli* as well.^7^ In snails, Cd/CuMT transcription occurs constitutively at very low levels and is unresponsive to metal exposure, suggesting that Cd/CuMT is of only marginal significance for the overall metal balance.^7,8^ Even though unselective, the behavior of Cd/CuMT in *C. aspersum* resembles more the one of CuMT than CdMT and was thus also described as a non-optimized CuMT.^16^ Since the metal-ion binding properties of the Cd/CuMT isoform are unspecific and unselective (for Cd^2+^ and Cu^+^), we prefer to call this isoform UnMT to more clearly distinguish it from the CdMT and the CuMT isoforms.

The three isoforms have their origin in two gene duplication events that occurred within Stylommatophora.^8,14^ The first duplication led to the split between CdMTs and CuMTs/UnMTs, whereas the second duplication led to the split between CuMTs and UnMTs. All three isoforms share the common architecture of two β-domains, each consisting of a metal-thiolate cluster composed of 9 Cys residues, separated by a two-residue linker. The Cys residues appear in highly conserved motifs, which means that metal preferences of the isoforms are encoded by non-Cys residues only.^8^ These motifs formed by conserved Cys residues are used to classify domains of mollusk MTs.^17–19^ In Stylommatophora, the N-terminal domain is a β_3_-domain and the C-terminal domain a β_1_-domain.^18^ For clarity, we refer to them here simply as N-domain and C-domain.

Using a metallomics approach, we and collaborators recently proposed that early Stylommatophora MTs were Cd^2+^-selective and that Cu^+^-selective MTs evolved later on.^14^ Here, we set out to use ancestral sequence reconstruction (ASR) on Stylommatophora MTs to study the evolution of metal binding and to identify substitutions that impacted metal preferences. The conformational properties of Cd^2+^- and Cu^+^-loaded extant and ancestral proteins were determined by nuclear magnetic resonance (NMR) spectroscopy. As noted by us previously^14,20^ and demonstrated in this work in detail, [^15^N,^1^H]-HSQC spectra and backbone ^15^N T_2_ relaxation times are useful proxies for the conformational stability of MTs, and hence heteronuclear NMR allows to conveniently follow the evolution of metal preferences. We hypothesize that MTs, which do not bind a specific metal ion well, undergo conformational exchange processes because the protein does not adopt a unique structure but rather samples different conformational states that are comparable in energy. This is similar to the approach of Palacios *et al.*,^12^ but differs in that NMR probes the protein directly, thereby complementing the mass spectrometry (MS) approach, which reports on the metal content.

## Materials and methods

### Ancestral sequence reconstruction

Gastropod MT sequences were aligned using MEGA7^21^ with MUltiple Sequence Comparison by Log-Expectation (MUSCLE). For MTs with more than two domains, only the C-domain and the adjacent N-domain were used for the alignment. Sequences with large deletions or alterations were not included, resulting in 61 sequences that were used for the ASR. The sequence alignment is provided in the supplementary data 1 (SD1) in Section 1. The phylogenetic tree used for the ASR was based on Dallinger *et al.*^14^ Some ambiguities were resolved using BEAST 2.6.0^22^ with random local clock^23^ and calibrated Yule model with priors according to the available phylogenetic tree. The resulting phylogenetic tree is shown in Fig. S1.1. The best substitution model was found to be Dayhoff + G + I using ProtTest 3.4.2.^24^ Sequences were separately reconstructed using marginal and joint reconstructions with FastML v3.11^25,26^ and Dayhoff model, MEGA7 with Dayhoff + G + I (5 gamma groups) based on ML and BEAST 1.10.4^27^ with Dayhoff + G + I. The consensus sequences of the different reconstructions were used for this study.

### Expression and purification of metallothioneins

MT samples were prepared based on the published protocol.^8^ MTs were expressed as fusion proteins with an N-terminal glutathione S-transferase (GST) tag using BL21(DE3) *E. coli* cells and a pGEX-4T-1 vector. Metals were supplemented during expression by the addition of 100 μM CdSO_4_ or CuSO_4_ to the M9-medium 1 h after induction with IPTG (0.2 mM at an OD_600_ of 1.0–1.2). The expression took place overnight at 30°C in 350 ml culture volume per baffled 2 L Erlenmeyer flasks. CuSO_4_-supplemented expressions were done under the exclusion of oxygen by purging the cell cultures with N_2_ for 5–10 min and sealing the flask with plastic wrap and parafilm for overnight expression. Pelleted cells were resuspended in lysis buffer (50 mM Tris, 100 mM NaCl, and 50 mM TCEP, at pH 8.0; 25 ml per pellet from one 350 ml culture) and lysed by sonication (Digital Sonifier, Branson; 20 min at 30% power, 1 s pulse on, 2 s pulse off) under a stream of N_2_ for Cu^+^-loaded samples. Cell debris was removed by centrifugation (18 000 rpm, 45 min, 4°C, SS-34 rotor). The fusion protein was immobilized using glutathione Sepharose 4B (GE Healthcare), which was first equilibrated with Tris-buffer (20 mM Tris, 20 mM NaCl, and 1 mM TCEP, at pH 8.0). Binding took place with 1 ml Sepharose 4B per 12.5 ml of supernatant for 1.5 h at room temperature (RT). After washing with thrombin-buffer (20 mM Tris, 100 mM NaCl, 2.5 mM CaCl_2_, and 1 mM TCEP, at pH 8.0; 15 ml buffer per 1 ml Sepharose 4B), the GST tag was cut off using thrombin (400 units per 1 ml Sepharose 4B). Cleavage took place overnight at RT in 2.5 ml thrombin-buffer per 1 ml of Sepharose 4B after purging with Ar and sealing with parafilm. The released MT was subsequently collected and concentrated using an Amicon Ultra-15 3 kDa MWCO Centrifugal Filter Unit. The thrombin cleavage site added two amino acids (GS) to the N-terminus of each MT construct (sequences are shown in SD1 in Section 1). MTs were further purified using size exclusion chromatography (SEC) with a HiLoad 16/60 Superdex 75 pg column or a Superdex 75 Increase HiScale 16/40 (GE Healthcare) (running buffer: 20 mM Tris, 20 mM NaCl, and 1 mM TCEP, at pH 8.0). For Cu^+^-loaded samples, the running buffer was continuously purged with N_2_ during SEC and the elution was collected under a stream of N_2_. Before performing NMR or MS measurements, Tris-buffer was exchanged to HEPES-buffer (20 mM HEPES, 20 mM NaCl, and 1 mM TCEP, at pH 7.0) using a PD-10 desalting column (GE Healthcare).

It should be noted that Cu^+^-loaded MTs were purified while extensively preventing the presence of oxygen. This included not only degassing buffers (as was done for the purification of Cd^2+^-loaded MTs) but also purging them with either N_2_ or Ar for 5–10 min before use. Additionally, sample solutions containing Cu^+^-loaded MTs were always purged with Ar when transferred to new containers, which were sealed with parafilm if the samples remained in them for longer than a few minutes. Cd^2+^-loaded samples were also often prepared using the same O_2_-exposure-preventing measures (except for expression) when prepared alongside Cu^+^-loaded samples for practical reasons.

### NMR experiments

All NMR samples contained uniformly ^15^N-labeled MTs (which were additionally ^13^C-labeled for triple-resonance experiments) at concentrations in the range of 20–200 μM for Cu^+^-loaded and generally at least 250 μM for Cd^2+^-loaded MT samples. Proteins were measured at pH 7 with 20 mM HEPES, 20 mM NaCl, and 1 mM TCEP, in 90% H_2_O/10% D_2_O (buffer diluted accordingly) on a Bruker AV-600 (for [^15^N,^1^H]-HSQCs and T_2_ relaxation series, both recorded with 16, 32, or 64 scans depending on sample concentration) or AV-700 spectrometer (for triple-resonance experiments used for assignments) at 298 K. [^15^N,^1^H]-HSQC spectra were recorded using a standard Bruker pulse program, with gradient selection and sensitivity enhancement^28^ as data matrices of 16 ppm (F2) and 35–40 ppm (F1) typically using 128–256 complex data points. ^15^N transverse relaxation (T_2_) was measured at 600 MHz with ^1^H-detected CPMG sequences^29,30^ using delays of 20, 50, 75, 100, 125, 150, 200, 250, and 300 ms. Delays of 200 and 75 ms were not recorded for Cd^2+^-loaded HpCuMT and a_4_UnMT. The T_2_ relaxation times for Cd^2+^-loaded HpCdMT were determined by us previously and thus not re-recorded here.^20^ Spectra were processed with Bruker's Topspin 3.6.5 and 4.2.0, and analyzed with CcpNmr v2.4 and v2.5.^31^ Assignment of Cd_6_-HpCuMT backbone resonances was done using triple-resonance spectra using CARA 1.9.1.

### Calculation of T_2_ relaxation times and peak inhomogeneity

Peak intensities from the T_2_ relaxation series were fitted to exponential decay curves to obtain values for the T_2_ time and the initial intensity (at the delay of 0 ms). Errors for T_2_ times were estimated using a Monte Carlo approach to incorporate the error of the initial intensity into the error of the T_2_ time. To achieve this, the value for the initial intensity was resampled 500 times based on a Gaussian distribution and the standard error from the fit. T_2_ times were fitted again using each of these resampled initial intensities. The thereby obtained T_2_ times were resampled themselves once based on the standard error from the fit. The standard deviation of these resampled T_2_ times was taken as the error of the fit. T_2_ times with errors larger than 25 ms were excluded from the data sets. T_2_ data are listed in the supplementary data 2 (Tables S23–S44).

The inhomogeneity of peak intensities was calculated using the following formula, where ‘*I*’ stands for signal intensity:


\begin{eqnarray*}
&&{I}^{Norm} = I/\bar{I}\\
&&S = \overline {\left| {{I}^{Norm} - 1} \right|} \end{eqnarray*}

Intensities were first normalized *(I^Norm^*) by division through their mean value $( {\bar{I}} )$. Next, the absolute deviations of these intensities from 1.0 were calculated, the mean of which gave the inhomogeneity (*S*). For each spectrum, only the 60 most intense peaks were included in the calculations of *I^Norm^* and *S*. When spectra had fewer than 60 peaks, the remaining intensities were given a value of zero to account for excessively broadened peaks that are absent in spectra. A total of 60 was chosen since it corresponds to the approximate number of peaks that can be expected for all constructs investigated if the protein adopts a unique structure. Peak intensities are listed in the supplementary data 2 (Tables S1–S22). Calculations and graphs were done using R v4.0.3 and v4.3.0^32^ with RStudio v1.4.1103 and 2023.03.1 + 446^33^ and the packages stringr,^34^ minpack.lm,^35^ and sinaplot.^36^ Structures were plotted using PyMol v2.4.2.

## Results

### Cd^2+^-loaded HpMTs

All *H. pomatia* MT (HpMT) isoforms (HpCdMT, HpCuMT, and HpUnMT) were expressed in *E. coli* with Cd^2+^-supplemented culture medium. The number of coordinated metal ions was determined by ESI-MS. HpCdMT and HpCuMT were found to exclusively bind 6 Cd^2+^ ions, whereas HpUnMT binds 6 and 7 Cd^2+^ ions (Fig. S2.1). HpUnMT exists in two single amino acid variants with either Val (HpUnMT1) or Ala (HpUnMT2) at position 32 (Fig. 3E). The binding of 6 Cd^2+^ ions by HpCuMT and HpUnMT suggests that both MTs possess the expected 2-domain architecture as known from HpCdMT.^20^ It is, however, unclear how the 7th Cd^2+^ ion is accommodated in HpUnMT, but NMR and MS data obtained from a single N-domain construct of HpUnMT2 suggests that the C-domain is required to bind it (Fig. S3.2). Our MS data differs from previously published results in which Cd^2+^-supplemented expression of HpCuMT and HpUnMT led to metal complexes with more heterogeneous metal loadings, including complexes that contain sulfide.^8,12^ These differences might stem from the different expression media that were used. While our expressions were done in M9, previous experiments utilized LB-medium. This explanation for the discrepancies in MS data is supported by recent findings, where glycosylation of MTs was detected when expressed in LB-medium but not in M9.^37^

**Fig. 1:**
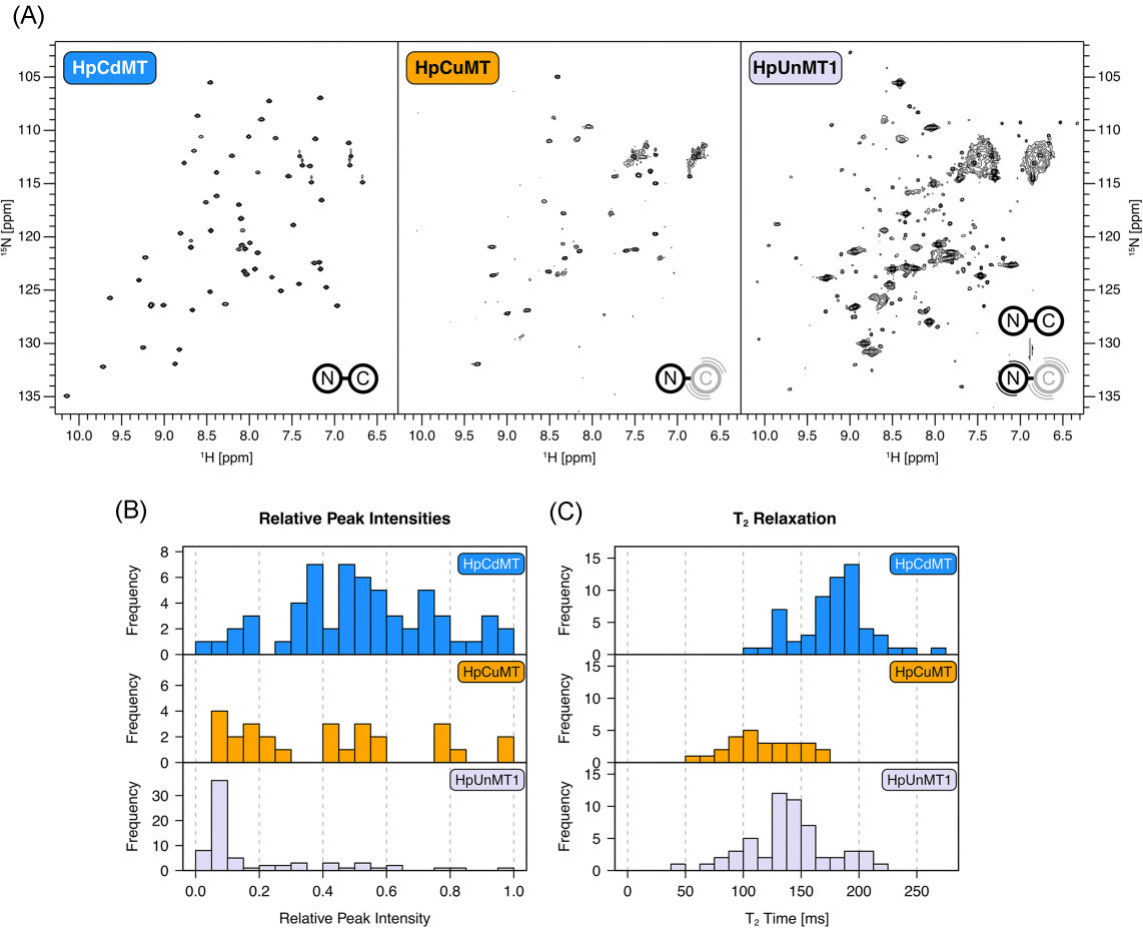
Spectra of Cd^2+^-loaded HpMTs. (A) [^15^N,^1^H]-HSQC spectra of HpCdMT, HpCuMT, and HpUnMT1. Spectra were recorded at 600 MHz at 298 K. Schematics of HpMTs are included to highlight the domains that undergo conformational exchange. (B) Histograms of peak intensities in each spectrum. Intensities were normalized by dividing through the maximum intensity within each spectrum to facilitate comparison. (C) Histograms of T_2_ relaxation times.

The [^15^N,^1^H]-HSQC spectra of the three *H. pomatia* isoforms binding Cd^2+^ ions are depicted in Fig. 1A. In [^15^N,^1^H]-HSQC spectra one peak is usually observed for each non-Pro residue due to the amide in each peptide bond. The intensity of the peaks is determined by the ^15^N transverse relaxation (T_2_) time, which depends on various factors, including the chemical environment of the nucleus, the overall protein tumbling time, the flexibility of the residue, and the presence of conformational exchange. A well-folded (conformationally stable) protein populates a unique conformational state, which leads to fairly homogenous peak intensities. However, when conformational transitions exist the associated exchange results in additional contributions to T_2_ relaxation, leading to peak broadening and therefore loss of signal intensity. This coincides with decreased homogeneity of the peak intensities as long as the conformational changes do not affect the protein globally. The absence of peaks indicates that transitions occur in a millisecond regime (intermediate NMR time regime), whereas additional peaks indicate the presence of conformational transitions that are much slower. The spectrum of Cd_6_-HpCdMT displays the expected number of peaks, which are homogenous in intensity, indicating that HpCdMT is conformationally stable i.e. lacks exchange processes in the milliseconds time regime. This is not the case for the other Cd^2+^-loaded HpMTs (Fig. 1A). Even though HpCuMT was shown to be fully loaded with 6 Cd^2+^ ions by ESI-MS, only half of the expected peaks are present in the [^15^N,^1^H]-HSQC spectrum. Most observed peaks could be assigned to the N-domain using triple-resonance spectra (Fig. S3.3), indicating that the C-domain undergoes conformational exchange in the milliseconds regime.

In contrast to HpCuMT, both HpUnMT variants display more peaks than expected in their [^15^N,^1^H]-HSQC spectra (Fig. 1A). Resonances can be roughly separated into a set of strong and a set of weak peaks, whereas weak peaks are more numerous (Fig. 1B). The strong peaks match the number expected from a single domain and contain only one major chemical shift change between HpUnMT1 and HpUnMT2. This shift is likely caused by the substitution V32A, which is the only difference between both HpUnMT variants, suggesting that the strong peaks belong to the N-domain (Fig. S3.4). This was further confirmed by superimposing spectra with those from an N-domain-only construct of HpUnMT2 (Fig. S3.2). Therefore, as in HpCuMT, conformational exchange in the C-domain leads to peak broadening and the absence of strong C-domain peaks. However, the presence of weak, additional peaks suggest that additional conformational exchange occurs on a much slower timescale. Weak, additional peaks were also present with the N-domain-only construct of HpUnMT2 (Fig. S3.2), indicating that both domains exhibit slow exchange. The peaks did not show any interconversion in a ZZ-exchange experiment (Fig. S3.5), and hence exchange between conformational states occurs on timescales slower than 1 s^−1^ or doesn't take place at all.^29^ Further, the N-domain peaks of HpUnMT are generally broader than for HpCdMT and HpCuMT, indicating that the N-domain of HpUnMT is more dynamic than in the other two HpMTs.

Besides the number of peaks and their intensity distribution, it is also useful to determine T_2_ relaxation times directly to quantify conformational exchange. In the case of Cd^2+^-loaded HpMTs, backbone amide T_2_ times are the longest with HpCdMT, further indicating that this isoform possesses high conformational stability (Fig. 1C). In contrast, HpCuMT and HpUnMT display overall shorter T_2_ times, which indicates the presence of conformational exchange. Strong and weak peaks of HpUnMT do not systematically differ in their T_2_ times, however, the low-intensity peaks of HpUnMT account for the increased variation in T_2_ times. To conclude, the Cd-specific HpCdMT is the only isoform to display high conformational stability when binding Cd^2+^ ions, suggesting that it represents the only HpMT fully optimized for binding Cd^2+^ ions.

### Cu^+^-loaded HpMTs

Cu^+^-loaded HpMTs were obtained by supplementing Cu^2+^ to the expression medium. Cu^+^-loaded MTs are oxidation prone and contact with oxygen was therefore minimized during purification (see methods for details). All HpMTs were found to bind 12 Cu^+^ ions based on ESI-MS. Smaller populations of HpMTs binding 11 and/or 10 Cu^+^ ions were present in all cases as well (Fig. S2.1). This suggests that the Cu^+^-stoichiometries are either generally more heterogenous or that Cu^+^ ions are lost more easily from the proteins. The inherent instability of the Cu_12_-core would agree with previous studies that observed two weakly bound Cu^+^ ions in addition to a stable Cu_10_-core.^12,16,38^ However, as was the case with Cd^2+^-supplemented expressions, our MS results with Cu^2+^-supplementation differ from previous publications by being more homogeneous in their metal compositions.^8,12^ As with Cd^2+^-supplementation, this might be attributed to differences in expression protocols, or in this case also to different amounts of air exposure during purification.

It is unclear whether the 12 Cu^+^ ions are coordinated within the same two-domain structure as formed with divalent metal ions or whether the protein adopts a new fold. To test for the two-domain architecture, the 2-residue linker between the canonical domains in HpCuMT was replaced with the 8-residue long linker from the *Megathura crenulata* MT to spatially separate the two putative domains. This system was used by us previously to investigate domain interactions in HpCdMT and *Littorina littorea* MT (LlMT).^20,39^ The long-linker construct was still binding 12 (and 10) Cu^+^ ions based on ESI-MS and its [^15^N,^1^H]-HSQC spectrum showed some similarities with the HpCuMT spectrum, with many peaks displaying chemical shift changes (Fig. S3.6). Further, ^15^N {^1^H}-NOE data suggests that the longer linker decouples the two putative domains so that they tumble independently (Fig. S3.7). These observations suggest that Cu^+^ ions are distributed to the same two domains as formed by Cd_6_-HpCdMT. Since both domains contain the same number of Cys residues and are similar in length, the likeliest distribution of the 12 Cu^+^ ions is 6 per domain. A construct containing only the N-domain of HpCuMT suggests the binding of 6 Cu^+^ ions by MS (Fig. S3.8), which is in agreement with mouse MT4 β-domain that has been shown to form Cu_6_-complexes as well, although not as the major species.^40^

**Fig. 2:**
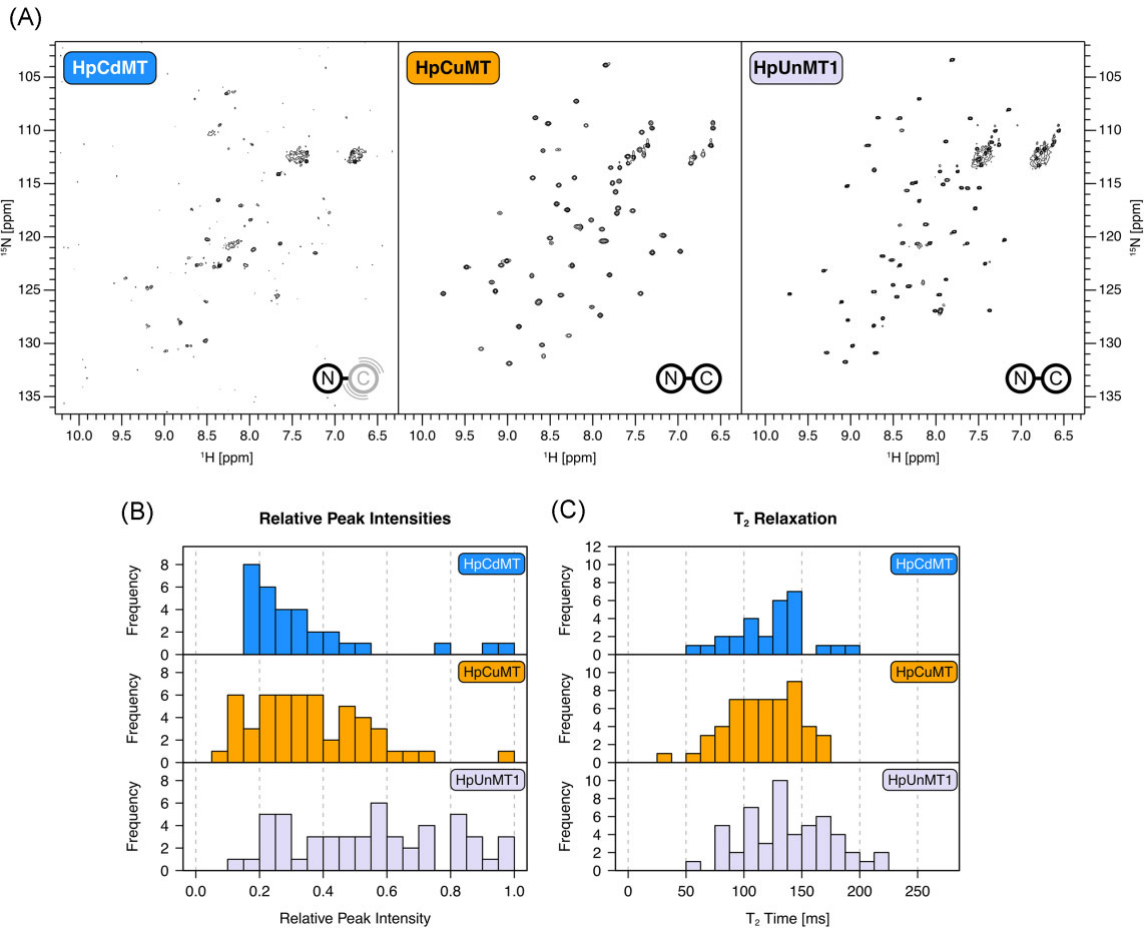
Spectra of Cu^+^-loaded HpMTs. (A) [^15^N,^1^H]-HSQC spectra of HpCdMT, HpCuMT, and HpUnMT1. Spectra were recorded at 600 MHz at 298 K. Schematics of HpMTs are included to highlight the domains that undergo conformational exchange. (B) Histograms of peak intensities in each spectrum. Intensities were normalized by dividing through the maximum intensity within each spectrum to facilitate comparison. (C) Histograms of T_2_ relaxation times.

Despite relatively similar ESI-MS spectra, considerable differences between HpCdMT and the other two isoforms are observed with NMR. [^15^N,^1^H]-HSQC spectra of HpCuMT and HpUnMT suggest conformational stability (Fig. 2A) on a similar level as observed for Cd_6_-HpCdMT (Fig. 1A). The Cu^+^-loaded HpCdMT, on the other hand, shows poor spectral properties that are mostly comparable to Cd^2+^-loaded HpCuMT, with roughly half of the expected peaks missing and many poorly defined weak peaks being present (Fig. 2A). Using chimeric MT constructs that combine domains from HpCdMT and HpCuMT, it is possible to attribute the missing peaks to the C-domain (Fig. S3.9). Therefore, in Cu^+^-loaded HpCdMT it is the C-domain that undergoes conformational exchange in the intermediate (milliseconds) regime. Consequently, the C-domain shows conformational intermediate exchange in HpCdMT as well as in HpCuMT when the non-cognate metal ions are bound. Further, intermediate exchange is present in the C-domain of HpUnMT when Cd^2+^ but not when Cu^+^ is bound.

HpUnMT displays the longest T_2_ relaxation times among the three isoforms (Fig. 2C), which are, however, still notably shorter than the longest T_2_ times obtained with Cd_6_-HpCdMT (Fig. 1C). Overall, the differences in T_2_ times between the Cu^+^-loaded HpMTs are not as pronounced as was the case with Cd^2+^-loaded HpMTs. Interestingly, even though HpCuMT gives very good spectra (Fig. 2A), it shows even slightly smaller T_2_ times than Cu^+^-loaded HpCdMT. Apparently, HpCuMT undergoes a considerable degree of conformational exchange despite binding its cognate metal. This contrasts with Cd^2+^-binding, for which HpCdMT had significantly increased T_2_ times that indicated a conformationally stable fold with the cognate metal ion. Taken together, HpCuMT and HpUnMT give rise to good-quality spectra, which indicates conformationally stable proteins. At the same time, however, T_2_ times reveal that there is still some conformational exchange present, particularly in HpCuMT. Since T_2_ times and peak intensities of HpCuMT are relatively homogenous, conformational exchange seems to similarly affect the entire protein. These conformational dynamics could be functional in HpCuMT as this isoform is involved in Cu-homeostasis and hence might require the release and transfer of Cu^+^ ions to other biomolecules or cellular compartments.^12^

### Ancestral sequences

**Fig. 3:**
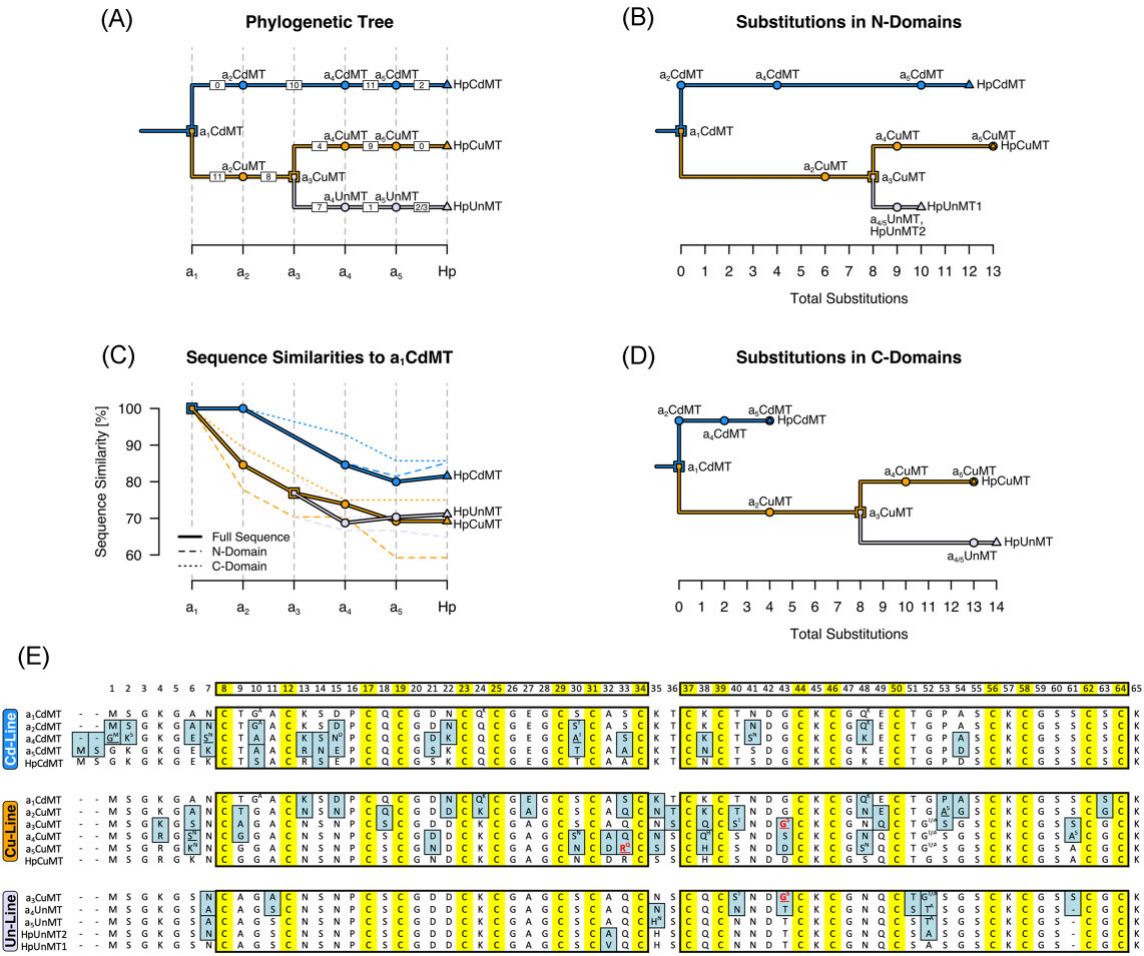
Reconstructed Stylommatophora MT sequences. (A) Phylogenetic tree with reconstructed MTs and extant HpMTs. Vertically aligned proteins were/are present in the same animal. a_3_CdMT is not present because the phylogenetic information allows only a_3_CuMT to be reconstructed although a_3_CdMT existed. Nodes (shown as squares) are ancestral proteins at gene duplication events and circles are ancestral proteins that *Helix pomatia* shares with other gastropods (a_2_MTs with *Arion vulgaris*, a_4_MTs with *Cochlicella acuta*, a_5_MTs with *Cornu aspersum*). Numbers in squares between proteins indicate how many substitutions occurred between them. (B, D) Phylogenetic trees with branch lengths that correspond to the total number of substitutions that occurred since a_1_CdMT in the N- (B) and the C-domain (D). (C) Sequence similarities of full sequences and single domains to a_1_CdMT as calculated with SIAS tool of the Immunomedicine group of the Universidad Complutense Madrid (http://imed.med.ucm.es/Tools/sias). (E) Sequence alignment of the reconstructed and extant MTs. Substitutions are highlighted in light blue and Cys residues in yellow. The domain structure is emphasized by black boxes, and amino acid numbering is shown at the top. Alternative residues are given as superscripts in cases where ambiguities were present between the four different methods used for the reconstruction (FastML (marginal and joint reconstructions), MEGA7, BEAST). If one method disagreed, then the alternative residue is given as a simple superscript, whereas if two methods disagreed, the residue was additionally underlined. Residues are highlighted in red for the two cases (a_3_CuMT and a_5_CuMT) in which all three methods disagreed with the main method used for the reconstruction (FastML, marginal reconstruction).

The three extant isoforms of HpMTs differ in their metal preferences. However, it remains an open question how metal-binding properties are determined by the amino acid sequence, in particular why certain MT sequences prefer one metal over another. Studying how evolution shaped metal binding is a promising approach to learn more about the factors that influence metal binding.^3^ To this end, the ancestral sequences leading up to the extant HpMTs were reconstructed (Fig. 3A and E). Ancestral MTs (aMTs) were numbered according to their position in the phylogenetic tree (a_1_MT to a_5_MT), thus, a_4_CdMT, a_4_CuMT, and a_4_UnMT for instance are isoforms that were present in the same ancestral animal (a_4_). Further, aMTs were labeled with the metal specificity of the descendant MT in *H. pomatia* based on the order of events as proposed by Dallinger *et al.*^14^ The oldest reconstructed ancestor MT (a_1_CdMT) forms the root of the Stylommatophora clade and is expected to have been Cd^2+^-specific (i.e. belonging to the Cd-lineage). This MT underwent a gene duplication event (110–180 Mya^14^) that led to the emergence of Cu-specific isoforms (i.e. the Cu-lineage), whereas the duplication of a_3_CuMT (90–110 Mya^14^) led to the emergence of unspecific MTs (i.e. the Un-lineage).

Many gastropod MTs are known to be selective and specific for Cd^2+^, making it likely that these are properties of the ancestral Stylommatophora MT as well.^14^ This appears to be reflected by the number of mutations occurring right after the first duplication event that led to the branching-off of the Cu-lineage: While the Cd^2+^-specific MT isoform remained perfectly conserved up to a_2_CdMT, the Cu-lineage MTs underwent 11 substitutions during the same time, decreasing their similarity to the ancestral a_1_CdMT considerably (Fig. 3C). This suggests that MTs in the emerging Cu-lineage adopted a new role soon after the gene duplication. Interestingly, seven out of the eleven substitutions from a_1_CdMT to a_2_CuMT are still present in HpCuMT. After the second duplication event, MTs in the Un-lineage underwent more substitutions than in the Cu-lineage, indicating that unspecific MTs likely diverged from Cu-specific MTs as has been noted previously.^14^ Interestingly, unspecific MTs remained highly conserved after a_4_MT, whereas MTs in the Cd- and Cu-lineages still underwent many substitutions.

N- and C-domains evolved with lineage-specific differences in their substitution rates (Fig. 3B and D). The C-domain of Cd-lineage MTs is the most conserved domain among all Stylommatophora MTs and underwent three times fewer mutations than the N-domain. This agrees with the observation that C-domains are generally stronger conserved than N-domains among all gastropod MTs.^14^ The stronger conservation of the C-domain appears to be linked to Cd^2+^-specificity that is shared among many gastropod MTs, since the C-domains of the Cu- and Un-lineage MTs underwent more than three times as many substitutions than in the Cd-lineage. In the Cu-lineage, both domains underwent a similar number of substitutions (Fig. 3B and D), however, the N-domain moved further away from its ancestral state in terms of sequence similarity (Fig. 3C). Therefore, for both the Cd- and the Cu-lineage, it is the C-domain that is conserved more strongly. The opposite is true for the Un-lineage, in which the C-domain underwent three times as many substitutions as the N-domain, mostly due to the large number of changes from a_3_CuMT to a_4_UnMT. Interestingly, a_4_UnMT displays the only instance of a single amino acid deletion among the studied sequences. Further, the N-domain in the Un-lineage is much more conserved than in the other two lineages with a single substitution occurring from a_3_CuMT to HpUnMT2, whereas there were 5 from a_3_CuMT to HpCuMT and 8 from a_4_CdMT to HpCdMT.

**Fig. 4:**
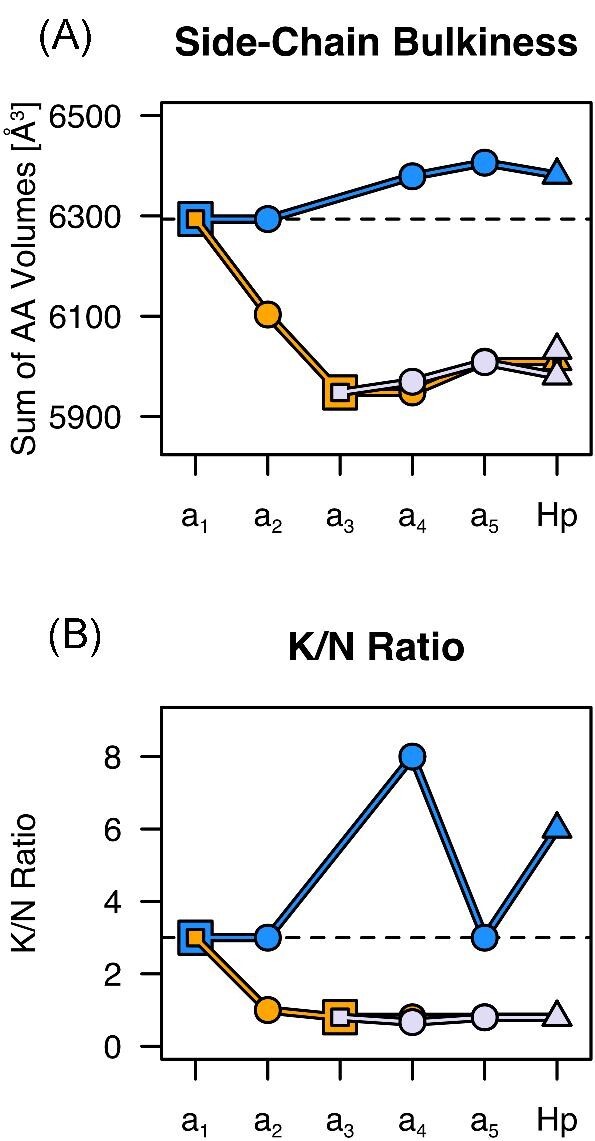
Change in side-chain bulkiness and K/N ratio. (A) Change in side-chain bulkiness throughout Stylommatophora MT evolution. Bulkiness was calculated by summing up the volumes of all residues. Amino acid volumes were taken from www.imgt.org and are based on Zamyatnin.^43^ Symbols and colors correspond to Fig. 3, with blue indicating the Cd-lineage, orange the Cu-lineage and light purple the Unlineage. (B) Change in K/N ratio throughout Stylommatophora MT evolution. Ratios of Cd-lineage MTs fluctuate strongly due to very low numbers of Asn within sequences (e.g. 1 Asn in a_4_CdMT and 2 Asn in a_5_CdMT). All residues N-terminal of the first Cys (C11) were not included in the calculations. Dashed lines indicate the values for a_1_CdMT.

Two general features that distinguish CdMTs from CuMTs have been described in the literature and can be followed through the reconstructed sequences (Fig. 4). One of them is the bulkiness of side chains, where CdMTs typically contain bulkier side chains than CuMTs.^12^ Less bulky side chains in CuMT might result in higher flexibility that provides a higher versatility for Cu^+^ ion coordination.^12^ The other general feature is the ratio of Lys to Asn residues (K/N ratio), which is higher in CdMTs than in CuMTs.^41,42^ Both features change notably with a_2_CuMT, whereas side chain bulkiness changes further with a_3_CuMT before reaching the value of HpCuMT and HpUnMT.

### Evolution of metal binding

**Fig. 5:**
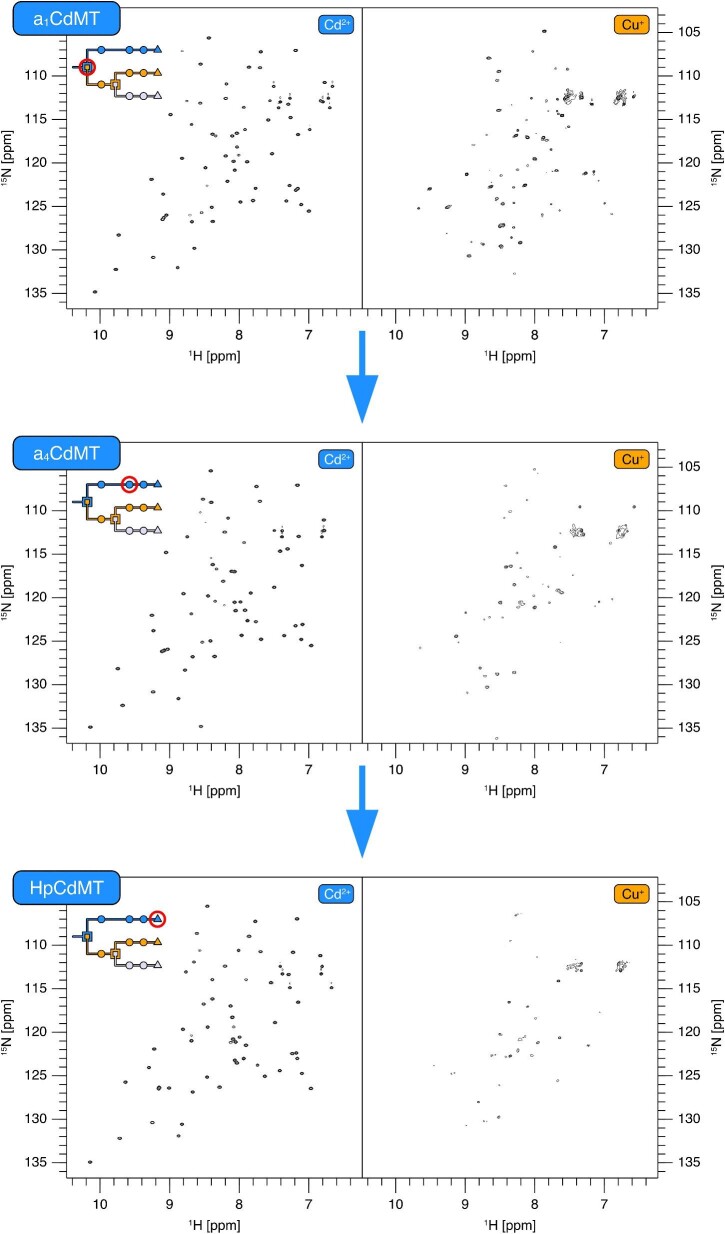
[^15^N,^1^H]-HSQC spectra of MTs in the Cd-lineage. The change in Cd^2+^- und Cu^+^-binding properties of MTs in the Cd-lineage is shown based on a_1_CdMT, a_4_CdMT, and HpCdMT. Spectra of Cd^2+^- and Cu^+^-loaded MTs are shown on the left and right, respectively. The small phylogenetic trees indicate the evolutionary position of the constructs. Note that the spectra of Cd^2+^-loaded MTs are qualitatively very similar to one another, whereas the spectra of Cu^+^-loaded MTs become worse, which can be seen most easily by the reduced number of peaks. The spectra of all constructs are shown in Fig. S3.1.

Ancestral MTs (except a_5_MTs) were expressed recombinantly in metal-supplemented media. The a_5_MT isoforms were not investigated due to their high similarities to the HpMT isoforms. The spectra of MTs from the Cd-lineage are shown in Fig. 5 and the spectra of all constructs are displayed in Fig. S3.1. Due to the disagreement between reconstruction methods, two variants of the a_3_CuMT were studied: a_3_CuMT1 with a Gly and a_3_CuMT2 with a Ser at position 43. This, as well as other discrepancies in the reconstructed sequences mostly concern the timing of when a substitution occurred: it seems likely that a_2_CuMT had a Gly and that a_4_CuMT had a Ser at position 43. However, it is less clear whether the substitution to Ser occurred already in a_3_CuMT or only in a_4_CuMT.

The ancestral proteins showed similar ESI-MS properties as the extant HpMTs. Supplementing Cd^2+^ to the expression medium led consistently to the coordination of 6 Cd^2+^ ions per molecule (Fig. S2.1). The only exception was a_4_UnMT, which was found to bind 7 Cd^2+^ ions in addition to the 6 Cd^2+^ complex, thereby resembling its descendant, HpUnMT. Cu^2+^-supplementation led again to MTs that bound 12 Cu^+^ ions, and, to varying degrees, also 11 and 10 Cu^+^ ions (Fig. S2.1).

**Fig. 6:**
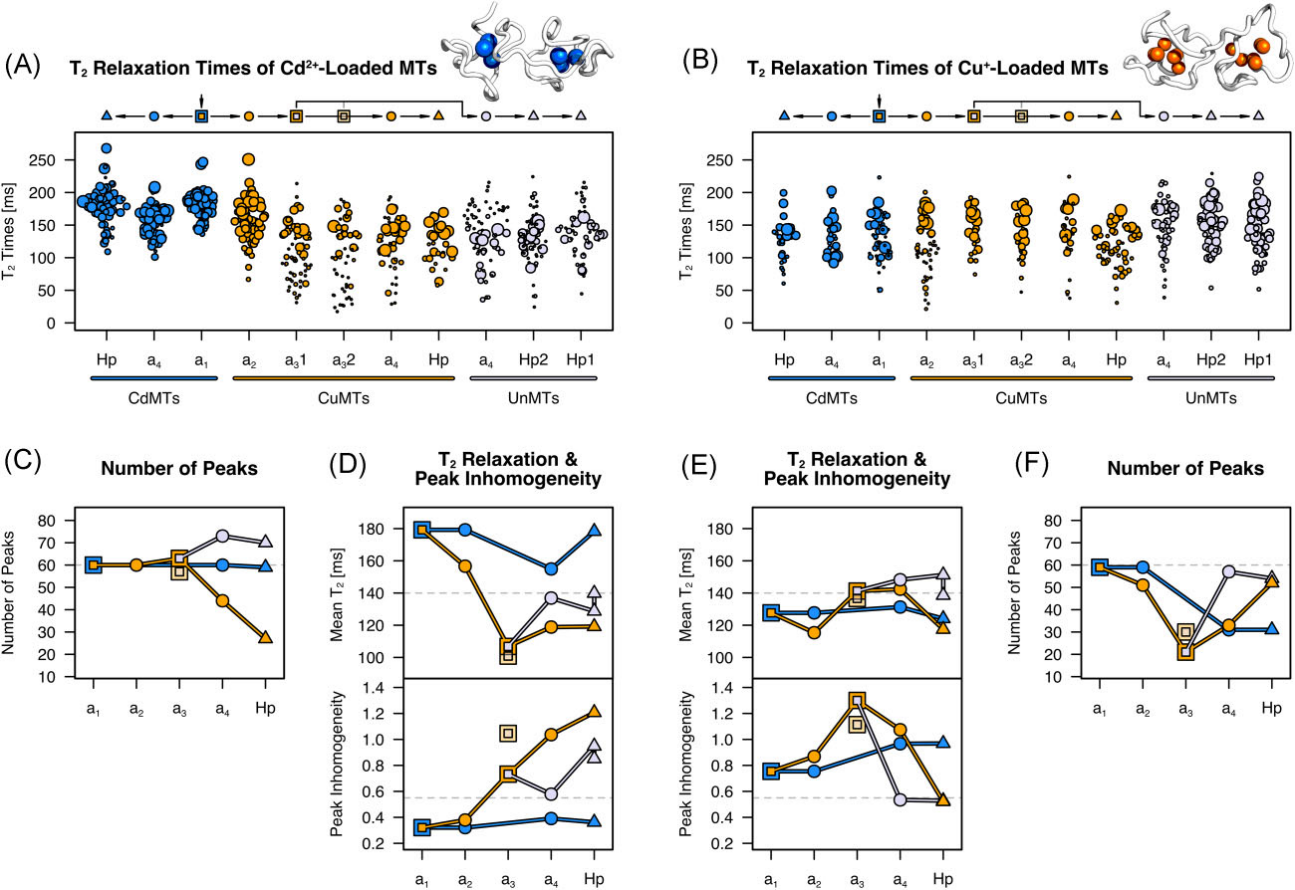
Evolution of Cd^2+^- and Cu^+^-binding in Stylommatophora MTs. (A, B) All obtained T_2_ relaxation times are displayed for each MT when binding Cd^2+^ (A) and Cu^+^ (B) ions. Point sizes correspond to max-normalized signal intensities. The phylogenetic relationships are indicated above the plot. (C, F) Conformational stability probed by the number of peaks in the [^15^N,^1^H]-HSQC spectra with Cd^2+^ (C) and Cu^+^ (F). The dashed line at 60 indicates the approximate number of peaks that can be expected for a conformationally stable MT. The values for a_2_CdMT were taken from a_1_CdMT since both are identical in sequence. (D, E) Conformational stability probed by the average T_2_ relaxation time and the inhomogeneity of peak intensities. A large mean T_2_ relaxation time and a low peak inhomogeneity indicate high conformational stability. Dashed lines at a T_2_ time of 140 ms and a peak inhomogeneity of 0.55 were included as an approximate delimiter for low and high conformational stability. Symbols and colors correspond to Fig. 3, with blue indicating the Cd-lineage, orange the Cu-lineage, and light purple the Unlineage. The variant a_3_CuMT2 with the substitution G43S is indicated by a box in lighter orange.

The conformational stability of the reconstructed MTs was assessed in the same manner as for the HpMTs: First, we simply counted the number of peaks in the [^15^N,^1^H]-HSQC spectra (Fig. 6C and F). If multiple conformational states exist that exchange much slower than ms^−1^, more than the expected number of peaks are present. If conformational exchange occurs in the intermediate regime (milliseconds), fewer than the expected peaks are present due to broadening. Second, we looked at average T_2_ relaxation times, which provides an estimate of the overall extent of conformational exchange (Fig. 6D and E). Third, we assessed how homogenously peak intensities are distributed (Fig. 6D and E). Structurally well-behaved (i.e. conformationally stable) proteins have long average T_2_ times, the expected number of peaks, and little peak inhomogeneity. If part of the protein becomes unstable (i.e. undergoes conformational exchange), peak inhomogeneity increases without necessarily changing the average T_2_ time notably. On the other hand, a protein might have homogenous intensities but low T_2_ times when all amides experience similar chemical exchange rates, for example due to concerted, protein-spanning conformational transitions. We like to emphasize that when peaks are missing, average T_2_ relaxation times probe only the remaining peaks, whereas peak inhomogeneity also takes missing peaks into account by giving them an intensity of zero (see methods for details).

a_1_CdMT is the ancestor to all Stylommatophora MTs and shows Cd^2+^-binding properties similar to the extant HpCdMT (Fig. 6). Overall, the Cd^2+^-binding properties in the Cd-lineage remain essentially the same throughout the ≥110 Myr^14^ that Stylommatophora has existed. Nevertheless, minor differences are observed, such as a small but systematic decrease in T_2_ times for a_4_CdMT compared to HpCdMT, and a larger spread of T_2_ times in HpCdMT compared to a_1_CdMT. In terms of Cu^+^-binding, the most pronounced change over time in the Cd-lineage is found in the number of peaks (Fig. 6F). Whereas, a_1_CdMT (and thus the identical a_2_CdMT) displays the expected number, many peaks are missing in the spectra of a_4_CdMT and HpCdMT. In HpCdMT, the C-domain peaks are absent, implying that this is likely the case for a_4_CdMT as well. Overall, the C-domain of a_1_CdMT binds Cu^+^ at higher conformational stability than does the C-domain in HpCdMT, whereas the N-domain keeps its level of conformational stability toward the non-cognate Cu^+^. This is best seen in the constant average T_2_ times throughout evolution, which only probes the N-domain in HpCdMT due to the missing C-domain peaks (Fig. 6E).

The divergence of the Cu-lineage from the Cd-lineage was marked by eleven substitutions up to a_2_CuMT, which led to a strong decrease in sequence similarity to the ancestral a_1_CdMT. However, these mutations do not significantly alter the overall Cd^2+^- and Cu^+^-binding properties of a_2_CuMT compared to the ancestral a_1_CdMT (Fig. 6). The most pronounced change is in the distribution of T_2_ relaxation times of the Cd^2+^-loaded a_2_CuMT, which become more heterogeneous (Fig. 6A), suggesting that parts of the protein are, to a small extent, affected by conformational exchange. There are also several weak peaks with small T_2_ times in Cu^+^-loaded a_2_CuMT, which could be the first indication of a deterioration in Cu^+^-binding properties within the Cu-lineage.

Conformational stability with Cd^2+^ within the Cu-lineage deteriorates markedly in the next step, from a_2_CuMT to a_3_CuMT, which differ by eight substitutions, four of which are still present in HpCuMT. However, the number of peaks still corresponds to the expected number. Only with a_4_CuMT peaks disappear and the spectrum approaches the appearance of Cd^2+^-loaded HpCuMT. The spectral changes of Cd^2+^-binding a_3_CuMT to a_4_CuMT and further to HpCuMT suggest that the weak peaks in a_3_CuMT belong to the C-domain. In the Un-lineage, a_4_UnMT resembles its successor, HpUnMT, in displaying too many peaks, again with a set of strong peaks corresponding roughly to the number expected from a single domain and a set of more numerous weak peaks. These observations suggest that the C-domain undergoes conformational exchange in a_4_UnMT and possibly a_3_CuMT (Fig. 6A) and therefore, appears to bind Cd^2+^ ions in conformationally less stable complexes than the N-domain.

Whereas, the Cu-lineage shows a clear deterioration in Cd^2+^-binding properties after their split from the Cd-lineage, no directly corresponding improvement in Cu^+^-binding can be found from a_2_CuMT to a_3_CuMT. Conformational stability with Cu^+^ even decreases and reaches a minimum with a_3_CuMT (Fig 6E and F), after which Cu^+^-binding improves independently in the Cu- and the Un-lineages. Conformational stability is much less pronounced in a_4_CuMT than in a_4_UnMT, the stability of which already closely resembles HpUnMT. In the Cu-lineage, it is only with the extant HpCuMT for which all peaks appear in the spectrum and where peak intensities are fairly homogenous. This might reflect the larger number of substitutions that occur in the C-domain of the Un- than the Cu-lineage after a_3_CuMT. Compared to Cd^2+^-loaded MTs, all Cu^+^-loaded MTs have low T_2_ relaxation times, which indicates that a considerable amount of conformational exchange is present in all studied MTs. Therefore, Cu^+^-binding appears to be inherently associated with a higher degree of conformational exchange compared to Cd^2+^-binding.

Even though the C-domain undergoes many more mutations in the Un-lineage than in the Cu-lineage from a_3_MT to a_4_MT (Fig. 3D), two substitutions in the Cu-lineage have counterparts in the Un-lineage: a G43S substitution in a_4_CuMT vs. a G43T substitution in a_4_UnMT and a S61A substitution in a_4_CuMT vs. a deletion at this position in a_4_UnMT. The substitution from Gly to Ser at position 43 can be studied by comparing a_3_CuMT1 (Gly43) with a_3_CuMT2 (Ser43), which shows that this substitution alone increases conformational exchange to some degree for Cd^2+^-binding (Fig. 6D). Interestingly, G43S has a corresponding positive but smaller effect on Cu^+^-binding properties (Fig. 6E).

The most discussed residue in HpCuMT is the sole His at position 38, located between two Cys residues in the C-domain.^12,38^ From bacterial, fungi, and plant MTs, His is known to participate in metal coordination.^44–47^ In HpCuMT, however, H38 appears to be unimportant for Cd^2+^-binding and the mutation of H38 to Ala even improved Cu^+^-binding properties by promoting more stable homonuclear complexes with tighter bound Cu^+^ ions.^12,38^ This gave rise to the speculation that the role of this His residue is rather to facilitate Cu^+^ release, allowing the transfer of Cu^+^ ions to other biomolecules such as hemocyanin.^12,38^ This agrees with our observation that HpCuMT undergoes a considerable amount of conformational exchange. H38 appeared first in a_5_CuMT, but conformational exchange is already very pronounced before a_5_CuMT in the Cu-lineage. This suggests that H38 is not the sole reason for the generally low conformational stability in HpCuMT, but that it might be responsible for the increased conformational exchange in terms of mean T_2_ time from a_4_CuMT to HpCuMT. Interestingly, a substitution to His also occurs from a_4_UnMT to HpUnMT2 in the linker between the domains, which, however, does not affect Cu^+^ binding properties.

### Conformity as a measure of metal preference

**Fig. 7:**
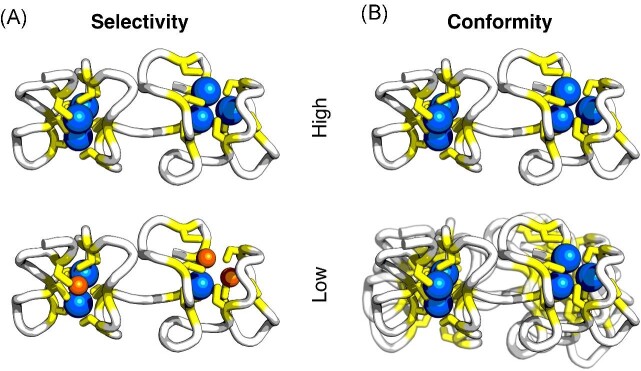
Metal selectivity and conformity. (A) Selective MTs form homometallic complexes with a defined stoichiometry when binding their cognate metal ion. (B) Conform MTs form conformationally stable complexes when binding their cognate metal ion. The structure of Cd_6_-HpCdMT (PDB entry 6QK6) is displayed.

MT-metal complexes have been extensively characterized using MS. If a MT forms homometallic complexes with a defined metal stoichiometry, then it is considered to be selective for that metal.^11,12,14^ Here, we provide complementary measures for the characterization of MT-metal complexes based on NMR, which expands our previous T_2_ relaxation-based approach.^14,20^ In contrast to MS, which probes the metal composition, our NMR approach gives information on the conformational stability of the protein, by characterizing the extent of conformational exchange (Fig. 7). This is done by counting peaks, quantifying the inhomogeneity of peak intensities, and measuring T_2_ relaxation times of backbone amides. A well-behaved protein populates a single, conformationally stable state and therefore lacks conformational exchange. This was found to be the case for Cd^2+^-loaded HpCdMT (a Cd^2+^-selective and Cd^2+^-specific MT), which suggests that the metal ions are accommodated in an optimal way that leads to a single, well-defined energy minimum in its folding landscape. To distinguish our measure from the MS-derived selectivity, we suggest the term conformity. HpCdMT would thus be a Cd^2+^-conform MT since the binding of Cd^2+^ ions causes HpCdMT to adopt a uniform conformational state: HpCdMT conforms to Cd^2+^.

The metal conformities of HpCdMT and HpCuMT mostly correspond to their metal selectivities. However, selectivity and conformity don't need to agree with one another: HpUnMT is Cu^+^-conform but unselective for Cu^+^. The discrepancy could stem from the fact that selectivity mostly reflects affinity or competition for different metal ions, whereas conformity reflects the energetic aspects of binding a single type of metal ion. Thus, HpUnMT might have evolved to bind Cu^+^ ions, without the need to discriminate between Cu^+^, Cd^2+^, or other metal ions. Interestingly, the function that HpUnMT fulfills in the snail remains unknown.^7,12^ This contrasts with HpCuMT, which binds Cu^+^ selectively but is slightly less Cu^+^-conform than HpUnMT, as is reflected in the higher degree of conformational exchange.

### Specializations of the N- and the C-domain

**Fig. 8:**
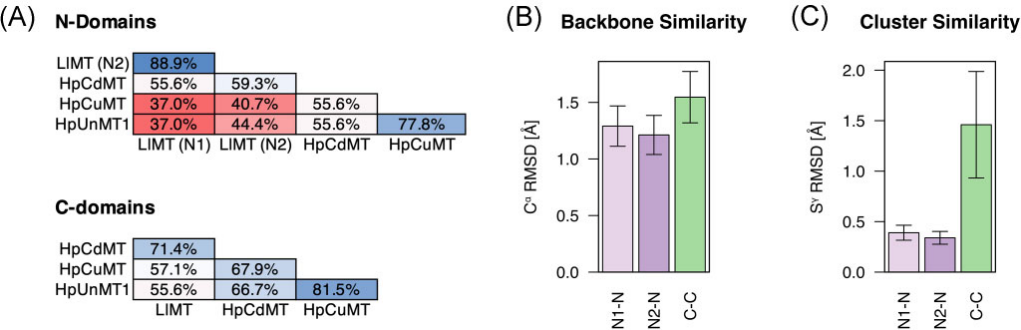
N- and C-domain conservation in sequence and structure. (A) Sequence similarities between HpMT isoforms and LlMT, which has a three-domain (N1-N2-C) architecture. N- and C-domain sequence similarities were calculated using the SIAS tool of the Immunomedicine group of the Universidad Complutense Madrid (http://imed.med.ucm.es/Tools/sias). (B, C) Root mean square deviations (RMSD) for C^α^ (B) and Cys-S^γ^ (C) atoms were calculated as previously done by Beil et al.^20^ Plots show averages of all pairwise RMSDs calculated between structures of the HpCdMT (6QK6)^20^ and of the LlMT (5ML1)^39^ structural bundles. Error bars indicate standard deviations.

The two-domain organization is overall conserved among gastropod MTs, even though exceptions can be found.^19,39^ The advantage of this two-domain organization is, however, less clear since both domains can act in isolation despite forming contacts in the native proteins.^20,39^ Further, the degree to which either domain is conserved varies, with C-domains being generally more conserved among gastropod MTs.^14^ However, a comparison between HpCdMT and the distantly related LlMT reveals that the N-domains are structurally more conserved than the C-domains (Fig. 8).^20^ Therefore, the N-domain appears to be the more generic scaffold for binding Cd^2+^ with a structure that is more resistant to mutations but displays lower affinities for Cd^2+^ than the C-domain.^14^

Similar differences between the domains appear among the aMTs as well. Most notable is the change in Cd^2+^-conformity of the C-domain. Whereas, all Cd-lineage MTs possess highly Cd^2+^-conform C-domains, the Cd^2+^-conformity is lost in the C-domains of HpUnMT and HpCuMT, and possibly their ancestors. This is contrasted by the N-domains, which generally moved further away from the ancestral sequence (in terms of sequence similarity) than the C-domains but remain Cd^2+^-conform at a higher level than the C-domains. This could again highlight that the N-domain is the generalist, which can adapt to bind different metal ions more easily without losing conformity to the original metal. By comparison, the C-domain resembles a specialist that can accommodate only one type of metal ion well but does so with a higher affinity.

## Discussion

The involvement of MTs in metal detoxification is widespread in marine animals, including marine gastropods, and possibly represents the ancestral function of these proteins, which were adapted later on to fulfill other roles such as the homeostasis of essential metal ions.^14,18,42^ In all these different roles, MTs are expected to be optimized at the structural level for metal ion binding, and in some of them also for metal ion release or exchange. Depending on their metal-specific function, this optimization may also require the complete or partial ability to distinguish between different metal ions. However, how exactly MTs adapted their sequences and structures to differentiate between metal ions is still unclear. We reconstructed ancestral MTs to follow the evolution of the Cd^2+^-, Cu^+^-, and unspecific MT isoforms of the snail *H. pomatia*, to derive insight into how evolution shaped metal binding and to identify the underlying molecular features. We hypothesize that an optimal fit of the protein structure to the metal cluster results in a unique structure without any conformational exchange. Accordingly, we probed metal conformity by heteronuclear NMR because conformationally stable MTs give spectra with the expected number of peaks, homogenous peak intensities, and long average T_2_ relaxation times.

In the case of the Cd^2+^-specific HpCdMT, evolution led to a protein that adopts a single, conformationally stable fold, which can be traced back to the ancestral MT within Stylommatophora. The high Cd^2+^-conformity of a_1_CdMT agrees with the proposed function of the ancestral Stylommatophora MT in Cd^2+^ detoxification.^14^ The high Cd^2+^-conformity indicates that the metal ions are accommodated optimally within the protein structure, which might enhance affinity for Cd^2+^ ions and/or increase the discrimination between Cd^2+^ and other ions. Hence, Cd^2+^-conformity could be an adaptation of HpCdMT and its ancestors for Cd^2+^ detoxification. In contrast, Cu^+^-conformity appears most pronounced with the extant proteins but does not reach the same level of conformational stability as Cd^2+^-conformity. Even the most Cu^+^-conform MTs still show a considerable amount of conformational exchange. It is possible that these residual dynamics cannot be avoided due to structural restraints that are generally present with Cu^+^ complexes. Alternatively, they might be due to ancestral adaptations for Cd^2+^-detoxification, which trapped these MTs in a suboptimal state preventing the emergence of full Cu^+^-conformity. However, since HpCuMT is adapted for Cu-homeostasis, the residual conformational freedom present in this MT could be functionally important for the release of Cu^+^ ions.^12^

**Fig. 9:**
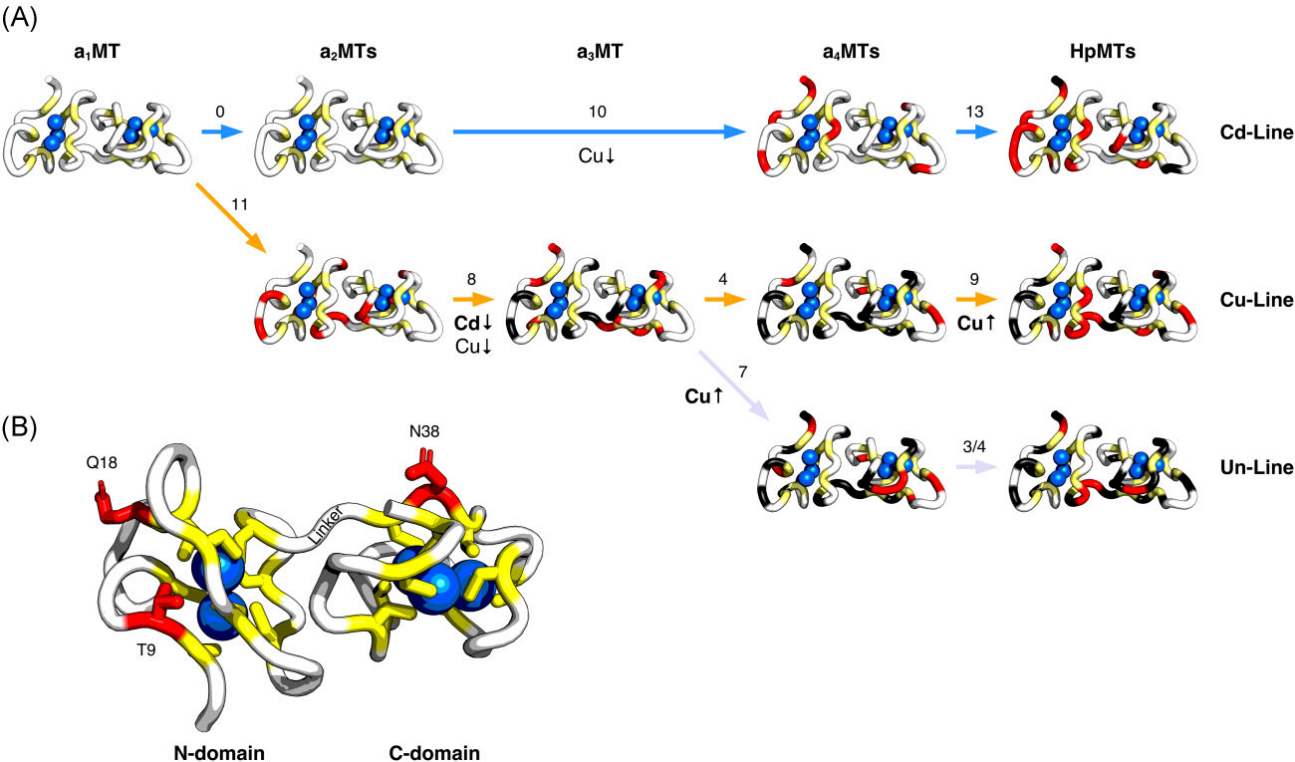
Evolution of Stylommatophora MTs with highlighted substitution sites. (A) Location of substitutions that occurred during the evolution of the Cd-, Cu- and Un-lineages color-coded on the structure of Cd_6_-HpCdMT (PDB entry 6QK6). Substitution sites are coded in red when the mutation occurred and changed to dark grey in later generations. Cys are depicted in yellow and Cd^2+^ ions as blue spheres. Changes in Cd^2+^- and Cu^+^- conformity are annotated in the figure. The largest changes in spectral quality are observed from a_2_CuMT to a_3_CuMT when loaded with Cd^2+^, and from a_3_CuMT to a_4_UnMT and a_4_CuMT to HpCuMT when loaded with Cu^+^. (B) The locations of T9, Q18, and N38 from discussed substitution sites are highlighted in red. N38 is substituted to a Gln in a_3_CuMT and subsequently to a His in a_5_CuMT. T9A and Q18S are substitutions occurring from a_2_CuMT to a_3_CuMT. Each domain is binding three Cd^2+^ ions (one is not visible in the N-domain here because it is located behind another Cd^2+^ ion).

Interestingly, HpCuMT and HpUnMT are both Cu^+^-conform, despite that no Cu^+^-specific function is known for the latter isoform. There is no continuous gain in Cu^+^-conformity after the divergence of the Cu/Un-lineage from the Cd-lineage, but rather Cu^+^-conformity evolved independently in the Cu- and the Un-lineage MTs (Fig. 9A). Many different hypotheses may be formulated why Cu^+^-conformity evolved late in either lineage, such as that Cu- and maybe Un-lineage MTs fulfilled Cu^+^-specific tasks only after a_3_CuMT or that Cu^+^-conformity required pre-adaptative substitutions to occur first (e.g. a substitution occurring in a_3_CuMT might have allowed Cu^+^-conformity to evolve). It is very interesting to note that during and before the gain in Cu^+^-conformity in the Un- and Cu-lineages, respectively, Cu^+^-conformity of the Cd-lineage declined. This could suggest that the Cd-lineage MTs were involved in Cu^+^-specific tasks until Un-lineage MTs replaced them, which in turn were replaced or complemented by Cu-lineage MTs later on.

Over the course of their evolution, Stylommatophora MTs underwent a large number of substitutions, with a total of 23 substitutions accumulating in the Cd-lineage without a major impact on Cd^2+^-conformity (Fig. 9A). Interestingly, mutations in the Cd-lineage differ from those in the Cu- or Un-lineage by the fact that they occurred less frequently in the C-domain. Further, mutations within the Cd-lineage itself occurred less frequently in the C- than in the N-domain, indicating that the C-domain tolerates fewer changes before Cd^2+^-binding properties deteriorate. Interestingly, the increase in Cu^+^-conformity in the Un-lineage coincides with a large number of mutations in the C-domain (5 out of 7 substitutions), suggesting that these improved Cu^+^-binding.

Many substitutions occurred in the Cu-lineage after the branching off from the Cd-lineage, with 11 substitutions being present in a_2_CuMT, 7 of which are still present in HpCuMT. These mutations neither improve Cu^+^-conformity nor notably decrease Cd^2+^-conformity despite being conserved. This suggests that these substitutions fulfilled roles other than changing metal binding properties, and/or that they might have been pre-adaptations that allowed Cu^+^-conformity to emerge later on. Overall, these findings suggest that both isoforms (a_2_CdMT and a_2_CuMT) were involved in Cd^2+^-detoxification (and maybe in Cu^+^-homeostasis) in the same organism.

In the Cu-lineage, Cd^2+^-conformity deteriorates from a_2_CuMT to a_3_CuMT. Two substitutions occur in the N-domain, T9A and Q18S (Fig. 9B), both of which reduce side-chain bulkiness and the number of functional groups that are potentially available for stabilizing interactions. At the same time, there are four substitutions in the C-domain (K38Q, T40S, E49Q, and A53S). The most notable of these substitutions might be K38Q (Fig. 9B) since a positive charge is lost and the K/N ratio is lowered. However, it is unclear whether the decrease in Cd^2+^-conformity stems from a small number of these substitutions or by complex interactions between them (e.g. through epistasis). The gain in Cu^+^-conformity happened independently in the Cu- and the Un-lineage and could be due to different mechanisms since no common substitutions occurred in both lineages. Due to the large number of mutations that occurred before Cu^+^-conformity improved, the impact of each of them, either individually or in an epistatic context, is difficult to deduce.

MTs fulfill complex biological functions, which puts a variety of constraints on the structures and sequences of these proteins. In addition to Cd^2+^-detoxification, HpCdMT appears to fulfill multiple other functions, as its transcription is potentially promoted in response to nonmetallic stressors as well.^48^ Further functional requirements like protein–protein interactions^4^ and metal-uptake promoting Cys orientations in apo-MTs^49^ will put additional constraints on sequences and structures that complicate the identification of metal-conformity shaping substitutions. We provide here new sequences and NMR data that make it possible to follow changes in metal binding properties and to identify associated substitutions, thereby providing a next step towards understanding metal selectivity and conformity of MTs. Further studies are encouraged to solve exactly how the amino acid sequence determines metal binding properties. We also have demonstrated that heteronuclear NMR is a convenient tool to investigate metal binding in MTs by probing metal conformity. This method presents a valuable complement to MS, given the fact that no time-consuming assignments are required, that ^15^N labeling is comparably cheap if heterologous expression in *E. coli* is possible, and that these measurements can be done at comparably low concentrations (10–100 μM). Its potential to look at molecular properties with residue resolution in our view makes this technique very attractive for such investigations.

## Limitations of this study

The quality of the reconstructed sequences depends primarily on the accuracy of the phylogenetic tree and the sequence alignment. Errors in either of them would give rise to reconstructed sequences that did not exist in that form. Recently, Calatayud *et al.*^18^ published many new gastropod MT sequences relevant to our study, which were not used for the ASR presented here and might thus test and improve the accuracy of our reconstruction. The conclusions from this study might further be tested by analyzing additional extant Stylommatophora MT sequences using our NMR approach. The proposed late occurrence of Cu^+^-conformity is falsifiable by the Cu^+^-conformities of other extant Cu^+^-specific and unspecific Stylommatophora MTs. If all of these MTs would prove to be Cu^+^-conform, it would seem highly improbable that they all descended from a non-Cu^+^-conform ancestor (e.g. a_3_CuMT) and evolved Cu^+^-conformity independently every time. A more general limitation of this study is that only fully metalated MT species were investigated, although partially metalated and apo MT species are biologically relevant as well. Therefore, not all metal-binding properties that are biologically important are covered by this study.

## Supplementary Material

mfad057_Supplemental_Files

## Data Availability

The data underlying this article are available in the article and in its online supplementary material.
