# Supplementary material for: Evolution of Cd2+ and Cu+ binding in Helix pomatia metallothioneins
Source: Metallomics. 2023 Sep 20;15(10):mfad057. doi: 10.1093/mtomcs/mfad057 (PMC10548783; doi:10.1093/mtomcs/mfad057)
Supplement: mfad057_Supplemental_Files [file mfad057_supplemental_files.zip › suppl_data_1_CLEAN.pdf]

Supplementary Data 1

Evolution of Cd^{2+} and Cu^{+} binding in *Helix pomatia* metallothioneins

Renato Valsecchi*, Christian Baumann*, Ardit Lila, Oliver Zerbe

<u>**Index**</u>

1. **Ancestral Sequence Reconstruction** **2**
2. **Mass Spectrometry** **6**
3. **NMR** **8**

1. Ancestral Sequence Reconstruction

Sequence selection is based on Dallinger et al. [1]. The sequence alignment used for the reconstruction is shown below. Sequences were aligned using MEGA7 with gap opening penalty of -3.9. Manual adjustments were made to improve the alignment. If multiple N-domains were present, only the N-domain adjacent to the C-domain was included.

```
>VetMegcre AAM51554.1
----------------------MSGKGENCTAECKSD-PCAC---GDSCKCGEG-CACTTCVKTEAKTTCKCG----ESCKC-EG-CKEGEACKCESG-CASCK---------------
>VetHaldiv ABW04628.1
---------------------MSSPQGPGCTASCKSE-PCAC---GTDCKCNPSDCPCTTC--K-DKTVCKCS----DGCQCGKG-CTTGDICKCDDS-CS-CK---------------
>VetHaldis AMS38479.1
---------------------MSSPQGAGCTGECKTD-PCAC---GTDCKCNPDDCACDTC--K-VKKTCKCP----GSCECGKG-CTSGETCKCDDS-CT-CK---------------
>VetHaltub GEAU01019532.1
----------------------MSSSGAGCTAECRSE-PCAC---GDDCRCDPKTCRCTEC-----RKTCTCT--E-AGCRCGRG-CTGPENCRCANA-CT-CKKPATKTYTRTASCHS
>VetHallae GFTT01185788.1
---------------------MSSPQGAGCTPECRSN-PCAC---GENCRCNPSDCVCTTC--K-VKKVCTCS----GVCQCGNG-CTGGDTCTCDDS-CR-CK---------------
>VetTegatr GFBI01039752.1
----------------------MSSTGEKCTTECKTT-PCAC---GTDCKCGPG-CACDSC--KDVKKACKCS----DSCKCGIG-CTGDDTCKCDNS-CS-CK---------------
>NerNerpel SRX644686
---------------------MSDPKGASCTTECKCD-PCAC---GTNCKCGSD-CTCSSC--K--KSSCKCA----DSCACGKG-CTGPSTCKCDSG-CS-CR---------------
>NerTitlim SRX644702
---------------------MSDTKPAGCTTECRTD-PCAC---GTNCKCTAE-CPCSAC--H--KPTCKCA--G-GPCACGKG-CTGPASCKCADD-CS-CH---------------
>NerNerpul1 QIR82898.1
---------------------MSDPKGASCTTECKCN-PCNC---GTNCKCGPD-CTCSSC--K--KSACKCS----GTCACGKG-CTGPDSCKCGAG-CS-CR---------------
>NerNerpul2 QIR82899.1
---------------------MPDPKGKGCTKECKAD-SCQC---GANCKCGGD-CPCKDC--H--KPTCSCS----GSCACGKG-CTGPETCKCADD-CS-CH---------------
>AmpMarcor1 QIR82902.1
MSSSEAHSHHHGECAKECKKSKESCCGGACTDECKKT-PCNC---GDNCKCSDG-CRCQSC-----SAPCKCD----GTCQCGKG-CTGADSCKCDRK-CS-CK---------------
>AmpPombri1 AST14869.1
MSSSEAHAHHHGECAKECKKSKASCCESACTEECKKT-PCNC---GDKCKCSDG-CKCQSC-----SAPCKCD----GTCQCGKG-CTGADSCKCDRK-CS-CK---------------
>AmpMarcor2 QIR82903.1
----------------------MSSANPACTAECKKT-PCNC---GDRCRCVHG-CRCQSC-----SAPCKCR----GTCQCGVG-CTGATSCKCSRQ-CS-CK---------------
>AmpPomcan1 XP_025111372.1
----------------------MSSANPACTAECKKT-PCSC---GDRCQCADG-CRCETC-----SAPCKCR----DTCQCGEG-CTGTTSCKCPLK-CS-CK---------------
>LitPomele1 ARA71541.1
----------------MSTSGANVIYGAGCTGTCKQS-PCGCKNSAAGCGCKDD-CRCPAC-----AKSCKC-----GTCNCGKG-CTGPSNCKCDDG-CS-CK---------------
>LitPomele2 ARA71542.1
-----------------MSSSGANATGAGCTETCKES-PCGCKNSAAGCKCKDD-CQCTTC-----AKSCKCA----GTCNCGKG-CTGPNSCKCDGG-CP-CK---------------
>LitLitlit1 AAK56498.1
---------------------MSSVFGAGCTDTCKQT-PCGC---GSGCNCKED-CRCQSC-----STACKCA--A-GSCKCGKG-CTGPDSCKCDRS-CS-CK---------------
>LitLitlit2 AST14862.1
---------------------MSSVFGAGCTDTCKQT-PCGC---GSECNCKEG-CRCQSC-----STACKCA--A-GSCKCGKG-CTGPDSCRCDRS-CS-CK---------------
>CalBossp SRP042651
--------------------MSSTSTGKGCVDLCTEQ-SCGC--AKGGCDCGDL-CKCQTC------NPCKCG----GSCKCGNG-CTGPADCHCAKS-CTGCK---------------
>CalCrefor SRP042651
---------------------MSATLGAGCTDACNQK-PCGC-IDAGDCQCGAN-CPCLTC------NPCKCS----GACVCGQG-CTGRESCKCVRG-CS-CRK--------------
>BucAnehel QIR82900.1
------------------MSDTEAKHGDGCTDACKET-PCGC-AASGGCKCTGD-CNCTAC-------CCKCD--K-DECKCDQG-CTGPDNCKCEEG-CK-CKSSD------------
>BucTriobs FK716482.1
------------------------MEGTGCTDACKET-PCGC-ASSGGCKCTGN-CSCPSC-------SCKCG--K-GACKCDKG-CEGPGSCKCGPG-CT-CKKS-------------
>AnaAplcal QIR82901.1
----------------------MSGKGPNCTEACGGD-PCNC---ADSCQCGEG-CSCSAC------KKCLCT--A-ESCKCGVG-CQGPASCKCGSS-CG-CK---------------
>PteClilim GESV01103158.1
----------------------MSGKGTTMTEACDT--PCEC---GDNCQCGEE-CSCTSC------RKCKCTAEAAEGCKCGDD-CTCAESCNCS---CRG-K---------------
>PteLimret GBXC01058817.1
----------------------MPGTGPNCSETCKAAGDCNC---GADCKCGKS-CDCTSC-------NCKCS--V-DTCKCGVG-CEGPESCGCGGS-CS-CK---------------
>SacElycri AST14868.1
----------------------MSGKGLICTAPCTND-PCGC---GENCQCGHA-CSCHSC-------SCSCG----ADCKCNQATCHEKSNCKCEAS-CS-CRAK-------------
>SacElycor GBRW01068275.1
----------------------MSGKGASCPPTCRND-PCGC---GQDCQCGQD-CTCSSC-------SCLCG----NECKCTSASCSQGVRCRCDTL-CD-CRPK-------------
>HygPhyacu2 SRP042651
----------------------MSGKGPNCTEACTGE-QCTC---GDSCKCGEG-CNCPSC------KTCKCE----DNCKCGEG-CTGPSTCKCESSDCA-CK---------------
```

```
>HygPhycar SRP042651
----------------------MSGKGPNCTEACTGE-QCTC---GDSCKCGEG-CNCPSC------KTCKCE--D-NACKCGEG-CTGPSTCKCESSDCA-CK---------------
>HygPhygyr SRP042651
----------------------MSGKGP-CTEACTGE-QCNC---GDSCKCGEG-CNCPSC------KTCKCE--D-NACKCGEG-CTGPSTCKCEST-CA-CK---------------
>LimDerretcd1 QIR82908.1
----------------------MSGKGEKCTGDCKSE-PCKC---GQNCQCGND-CTCSQC------KTCKCS--TGSGCQCGHG-CTGVESCKCGSS-CT------------------
>LimDerretcd2 eSnail database CL8321.Contig1
----------------------MSGKGEKCTGDCKSE-PCKC---GQNCQCGND-CTCSQC------KTCKCS--S-SGCQCGHG-CTGVESCKCGSS-CT-CK---------------
>LimLehnyccd eSnail database CL1241.Contig1
----------------------MSGKGAKCTGACKSE-PCQC---GQNCQCGDD-CSCSQC------KTCKCS--AGSTCQCGHG-CTGVESCKCGNS-CS-CK---------------
>LimLimmaxcd AYL40761.1
----------------------MSGKGAKCTGACKSE-PCQC---GQNCQCGDD-CSCSQC------KTCKCS--AGSTCQCGHG-CTGVESCKCGSS-CS-CK---------------
>AriArivulcu AWD77147.1
----------------------MSGRG--CNGTCNSN-PCQC---EDGCQCGDA-CSCAQC------NTCKCT--N-DGCKCGNE-CTATGSCKCGTS-CG-CN---------------
>AriArivulcd AWD77146.1
----------------------MSGKA--CTGACKSE-PCQC---GNNCQCGGD-CDCSQC------KTCKCT--N-EGCKCGQN-CTGQATCSCEKS-CS-CK---------------
>ClaAlibipcu1 QIR82905.1
----------------------MSGKGANCTGACNSN-PCQC---GDDCKCGVG-CSCAEC------NTCKCT--N-DGCKCGHG-CTGAGSCKCGNS-CG-CK---------------
>ClaAlibipcu2 MK639793
----------------------MSGKGANCTGACNSN-PCQS---GDDCKCGVG-CSCAEC------NTCKCT--N-DGCKCGHG-CTGAGSCKCGNS-CG-CK---------------
>ClaAlibipcu3 QIR82904.1
----------------------MSGKGANCSGACNSN-PCQC---GDDCKCGAA-CSCAEC------NTCKCT--N-DSCKCGHD-CSGAGSCKCGNS-CG-CK---------------
>ClaAlibipcd1 QIH55674.1
----------------------MSGKA--CTGDCKSD-PCKC---GDNCQCGDG-CTCASC------KTCKCT--N-EGCKCGQE-CTGPATCKCASG-CS-CK---------------
>ClaAlibipcd2 QIH55673.1
----------------------MSGKA--CTGDCKSD-PCKC---GDNCQCGDG-CTCASC------KTCKCT--N-EACKCGQE-CTGPATCKCASG-CS-CK---------------
>HelCocacucd eSnail database Unigene52262
--------------------MSGKGKAESCTAQCQSN-PCQC---GDKCQCGEG-CACTSC------KTCKCT--S-DGCKCGKE-CTGPASCKCGSS-CS-CK---------------
>HelNessamcd ACC17831.1
----------------------MSGKGELCTSACKSN-PCQC---GDKCQCGEG-CTCSAC------KSCHCT--N-DGCNCGKE-CTGPTSCKCDTS-CS-CK---------------
>HelAriarbcd1 P55946.1
---------------------SGKGKGDLCTAACKNE-PCQC---GSKCQCGEG-CACASC------KTCNCT--S-DGCKCGKE-CTGAASCKCNSS-CS-CK---------------
>HelAriarbcd2 AAB47142.1
---------------------SGKGKGDLCTAACKNE-PCQC---GSKCQCGEG-CACASC------KTCNCT--S-DGCKCGKE-CTGAASCKCGSS-CS-CK---------------
>HelCoraspcd1 ABL73910.1
--------------------MSGKGKGEKCTAACRNE-PCQC---GSKCQCGEG-CTCAAC------KTCNCT--S-DGCKCGKA-CTGPDSCTCGSS-CG-CK---------------
>HelCoraspcd2 ABM66449.1
--------------------MSGKGKGEKCTAACRNE-PCQC---GSKCQCGEG-CTCAAC------KTCNCT--S-DGCKCGKE-CTGPDSCKCSSS-CS-CK---------------
>HelCephorcd1 AYL40762.1
--------------------MSGKGKGEKCTAACRNE-PCQC---GSKCQCGEG-CACAAC------KTCNCT--S-DGCKCGKE-CTGPDSCKCGSL-CS-CK---------------
>HelCephorcd2 AYL40763.1
--------------------MSGKGKGEKCTAACRNE-PCQC---GSKCQCGEG-CACAAC------KTCNCT--S-DGCKCGKE-CTGPDSCKCGSS-CS-CK---------------
>HelHelpomcd AAK84863.1
--------------------MSGKGKGEKCTSACRSE-PCQC---GSKCQCGEG-CTCAAC------KTCNCT--S-DGCKCGKE-CTGPDSCKCGSS-CS-CK---------------
>HelCocacucu eSnail database Unigene18701
----------------------MSGRGN-CGGACKSN-PCSC---GQVCKCGGA-CTCAQC------NACRCS--G-DSCKCGDQ-CTASGSCQCGSG-CG-CK---------------
>HelCoraspcu ABM55268.1
----------------------MSGRGQNCGGACNSN-PCNC---GNDCNCGTG-CNCDQC------SARHCS--N-DDCKCGSQ-CTRSGSCKCGNA-CG-CK---------------
>HelThepiscu eSnail database CL8321.Contig1
----------------------MSGRGKNCGGACNSN-PCNC---ANNCRCGAG-CNCDSC------SSCHCS--N-DDCKCGNQ-CTTSGSCKCGSA-CG-CK---------------
>HelCepnemcu GFLU01065470.1
----------------------MSGRGN-CGGACNSN-PCSC---GDDCKCGAA-CNCDRC------SSCHCS--N-DDCKCGSQ-CTGSGSCKCGSA-CG-CK---------------
>HelHelpomcu AAK84864.1
----------------------MSGRGKNCGGACNSN-PCSC---GNDCKCGAG-CNCDRC------SSCHCS--N-DDCKCGSQ-CTGSGSCKCGSA-CG-CK---------------
>HelCocacuun eSnail database Unigene65576
----------------------MSGKGSACAGSCGNN-PCSC---GDDCRCGAG-CSCAQC------NSCQCN--N-DTCKCGNQ-CSTSGSCKCGS--CG-CK---------------
>HelCoraspun ABM92276.1
----------------------MSGKGSACAGSCNSN-PCSC---GDDCKCGAG-CSCAQC------YSCQCN--N-DTCKCGSQ-CSTSGSCKCGS--CG-CK---------------
>HelCephorun AYL40764.1
----------------------MSGKASACAGSCNSN-PCSC---GDDCQCGAG-CSCAQC------HSCQCN--N-DTCKCGNQ-CSASGSCKCGS--CG-CK---------------
>HelCepnemun GFLU01003885.1
----------------------MSGKGSACAGSCNSN-PCSC---GDDCQCGAG-CSCAQC------QSCQCN--N-DTCKCGNQ-CSASGSCKCGS--CG-CK---------------
>HelHelpomun1 ACY71053.1
----------------------MSGKGSNCAGSCNSN-PCSC---GDDCKCGAG-CSCVQC------HSCQCN--N-DTCKCGNQ-CSASGSCKCGS--CG-CK---------------
>HelHelpomun2 ACY71054.1
----------------------MSGKGSNCAGSCNSN-PCSC---GDDCKCGAG-CSCAQC------HSCQCN--N-DTCKCGNQ-CSASGSCKCGS--CG-CK---------------
```

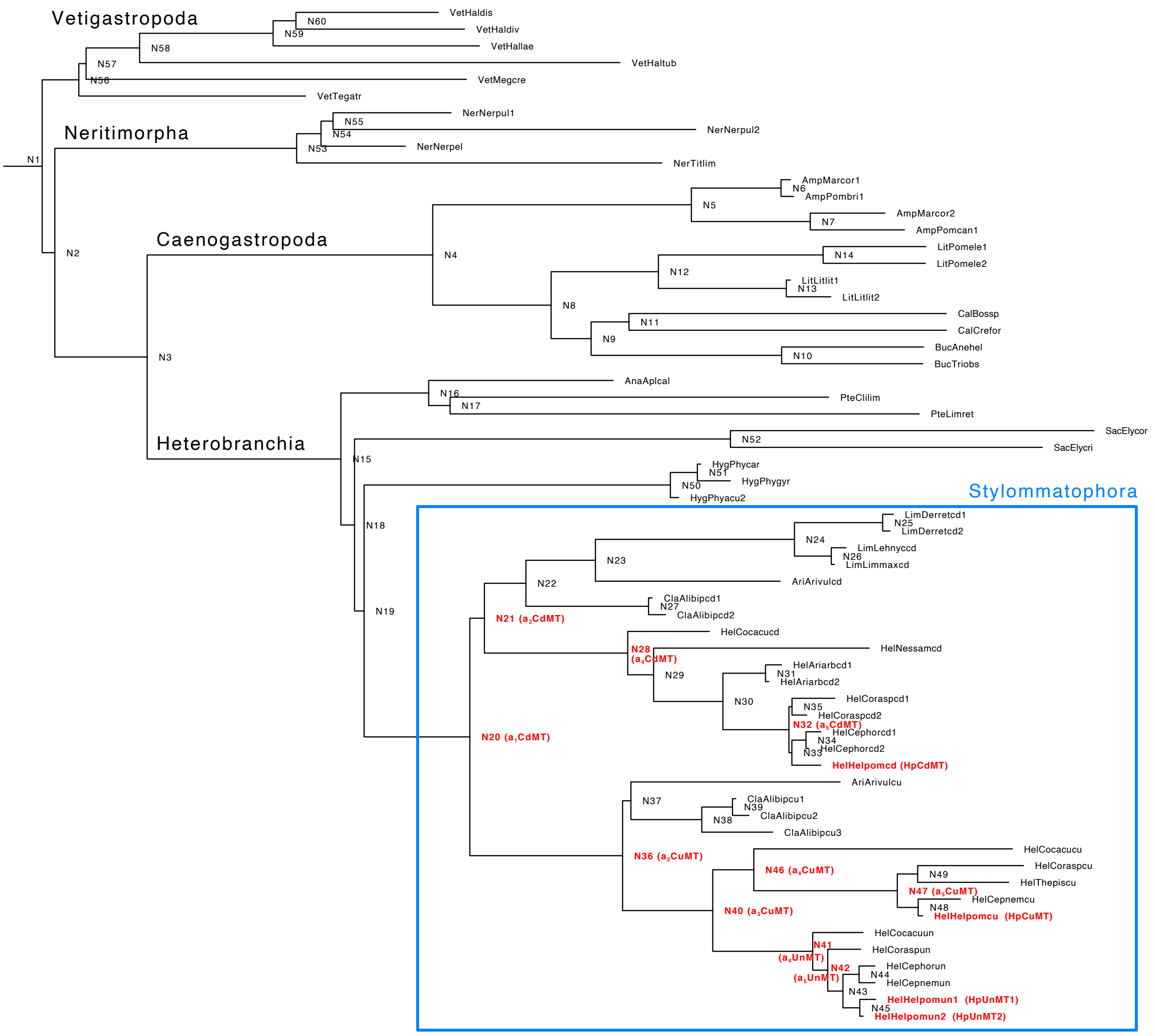

Fig. S1.1. Phylogenetic tree of Gastropoda MTs. The investigated MTs belong to the order Stylommatophora, which is highlighted by the blue box. The names of investigated MTs are shown in red. Extant MTs are abbreviated by clade name (3 letters), genus (3 letters), species (3 letters), and metal preference (2 letters, only for Stylommatophora MTs). For example, HelHelpomcd stands for "Helicoidea *Helix pomatia* Cd-specific" MT. We focused on Stylommatophora MTs due to an increased ambiguity for earlier sequences when the different reconstruction methods were compared.

Sequences were either ordered as G-blocks from Integrated DNA Technologies, Inc (Coralville, Iowa, United States) or obtained via site-directed mutagenesis (HpMTUnMT1 to HpMTUnMT2, a_3CuMT1 to a_3CuMT2). The N-terminal residues (GS or G) are artefacts from the thrombin cleavage site and are highlighted below when absent in the wild-type sequences. The sequences used in this study were:

Sequences of extant MTs:

>HpCdMT AAK84863.1

GSGKGKGEKCTSACRSEPCQCGSKCQCGEGCTCAACKTCNCTSDGCKCGKECTGPDSCKCGSSCSK

>HpCuMT AAK84864.1

GSGRGKNCGGACNSNPCSCGNDCKCGAGCNCDRCSSCHCSNDDCKCGSQCTGSGSCKCGSACGCK

>HpUnMT1 ACY71053.1

GSGKGSNCAGSCNSNPCSCGDDCKCGAGCSCVQCHSCQCNNDTCKCGNQCSASGSCKCGSCGCK

>HpUnMT2 ACY71054.1

GSGKGSNCAGSCNSNPCSCGDDCKCGAGCSCAQCHSCQCNNDTCKCGNQCSASGSCKCGSCGCK

Reconstructed MTs:

>a_1CdMT (N20)

GSGKGANCTGACKSDPCQCGDNCQCGEGCSCASCKTCKCTNDGCKCGQECTGPASCKCGSSCSCK

>a_4CdMT (N28)

GSGKGESCTAACKSNPCQCGDKCQCGEGCACASCKTCKCTSDGCKCGKECTGPASCKCGSSCSCK

>a_2CuMT (N36)

GSGKGANCTGACNSNPCQCGDDCKCGAGCSCAQCNTCKCTNDGCKCGNECTGAGSCKCGSSCGCK

>a_3CuMT1 (N40)

GSGKGSNCAGACNSNPCSCGDDCKCGAGCSCAQCNSCQCSNDGCKCGNQCTGSGSCKCGSSCGCK

>a_3CuMT2 (N40 G43S)

GSGKGSNCAGACNSNPCSCGDDCKCGAGCSCAQCNSCQCSNDSCKCGNQCTGSGSCKCGSSCGCK

>a_4CuMT (N46)

GSGRGSNCGGACNSNPCSCGDDCKCGAGCSCAQCNSCQCSNDSCKCGNQCTGSGSCKCGSACGCK

>a_4UnMT (N41)

GSGKGSACAGSCNSNPCSCGDDCKCGAGCSCAQCNSCQCNNDTCKCGNQCSTSGSCKCGSCGCK

Additional Constructs:

>HpCuMT-Mc-Linker

GSGRGKNCGGACNSNPCSCGNDCKCGAGCNCDRCVKTEAKTTCHCSNDDCKCGSQCTGSGSCKCGSACGCK

>N^{domain}-HpUnMT2

GSGKGSNCAGSCNSNPCSCGDDCKCGAGCSCAQCHS

>N^{domain}-HpCuMT

GSGRGKNCGGACNSNPCSCGNDCKCGAGCNCDRCS

>N^{domain}-HpCuMT/C^{domain}-HpCdMT

GSGRGKNCGGACNSNPCSCGNDCKCGAGCNCDRCSTCNCTSDGCKCGKECTGPDSCKCGSSCSCK

>N^{domain}-HpCdMT/C^{domain}-HpCuMT

GSGKGKGEKCTSACRSEPCQCGSKCQCGEGCTCAACKSCHCSNDDCKCGSQCTGSGSCKCGSACGCK

2. Mass Spectrometry

The mass analysis for the purpose of sample confirmation and metal load elucidation was commissioned to the Functional Genomic Centre Zurich (FGCZ) located at the Campus Irchel at UZH. The description of the analytical specifications was provided by the FGCZ and is summarized below.

Metal-protein complexes were desalted using C_{18} ZipTips (Millipore, Billerica, MA, USA). In order to preserve the complexes from dissociation, they were eluted with 12 mM NH_4Ac in 30 % MeOH or 50 % MeOH/iPrOH (pH 7.5 in any case). The solutions were infused through a fused silica capillary (ID75um) at a flow rate of 1 µL min^{-1} and sprayed through a PicoTips (ID30um). The former was obtained from either New Objective (Woburn, MA) or CoAnn Technologies (Richland WA, USA). Nano ESI-MS analyses of the samples were performed on a Synapt G2_Si mass spectrometer and the data were recorded with the MassLynx 4.2 Software (both Waters, UK). Mass spectra were acquired in the positive-ion mode by scanning m/z ranges of maximally 100 to 5000 and minimally 400 to 4000 Da with a scan duration of 1 s and an interscan delay of 0.1 s. The spray voltage was set to 2.7 or 3 kV, the cone voltage to 50 – 100 V (not respectively) and the source temperature was 80 °C. The recorded m/z data was then deconvoluted into mass spectra (monoisotopic masses) by applying the maximum entropy algorithm MaxEnt1 (MaxLynx) with a resolution of the output mass 0.02–0.03 Da/channel and Uniform Gaussian Damage Model at the half height of 0.1 Da.

Calculation of theoretical MT masses was done as described by Schicht et al. [2]. Complete labelling through supplemented isotopes was assumed. Deviations of <0.05 % between theoretical and experimental mass were tolerated for confirmation of the metalated complex.

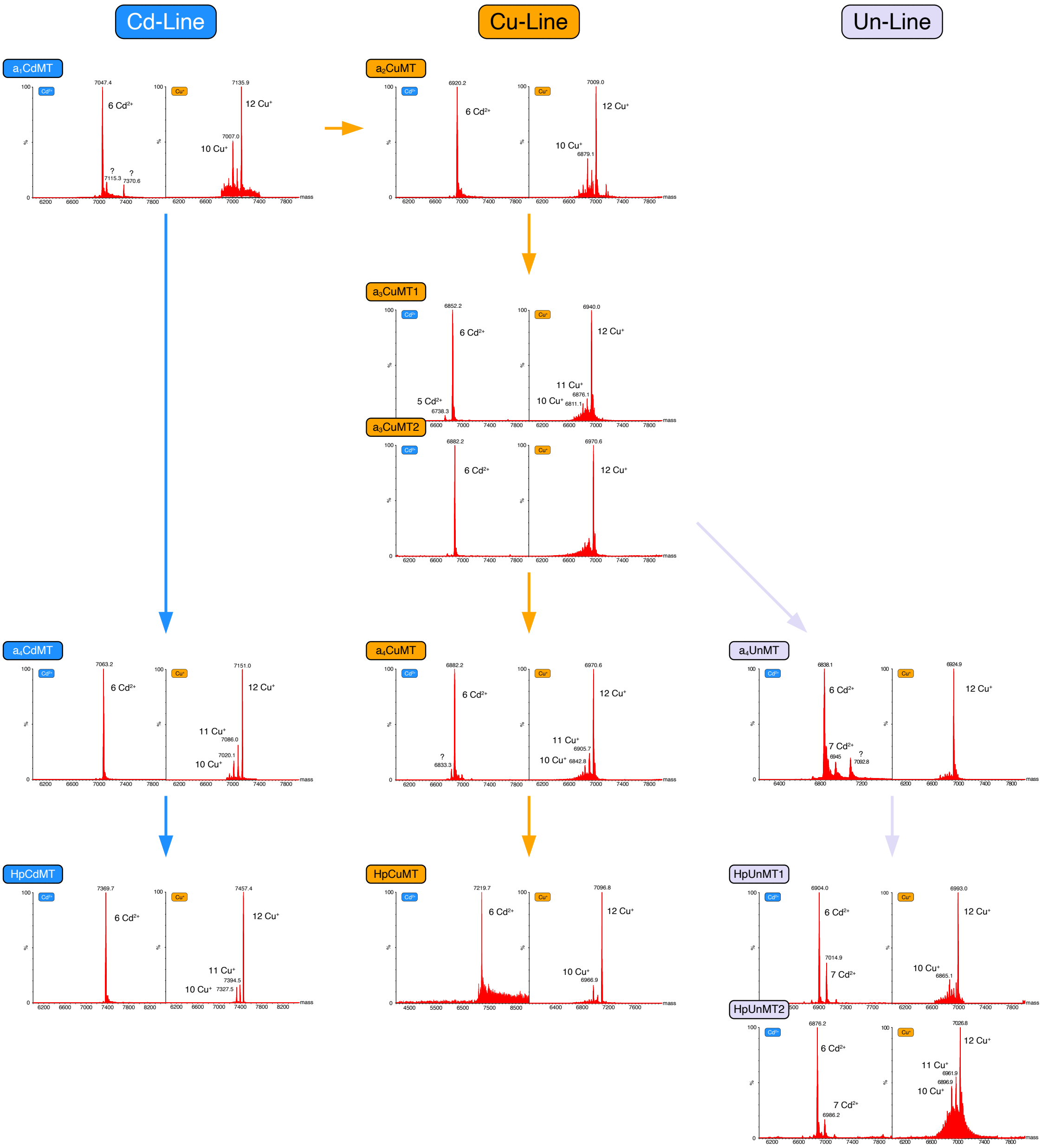

Fig. S2.1. Mass spectra of extant HpMTs and reconstructed ancestral MTs. Mass spectra are from uniformly ^{15}N-labelled proteins except for the spectrum of Cd$_6$-HpCuMT, which was recorded on a uniformly ^{15}N- and ^{13}C-labelled sample. Question marks denote peaks that could not be assigned. Small boxes inside the mass spectra indicate the type of metalation.

3. NMR

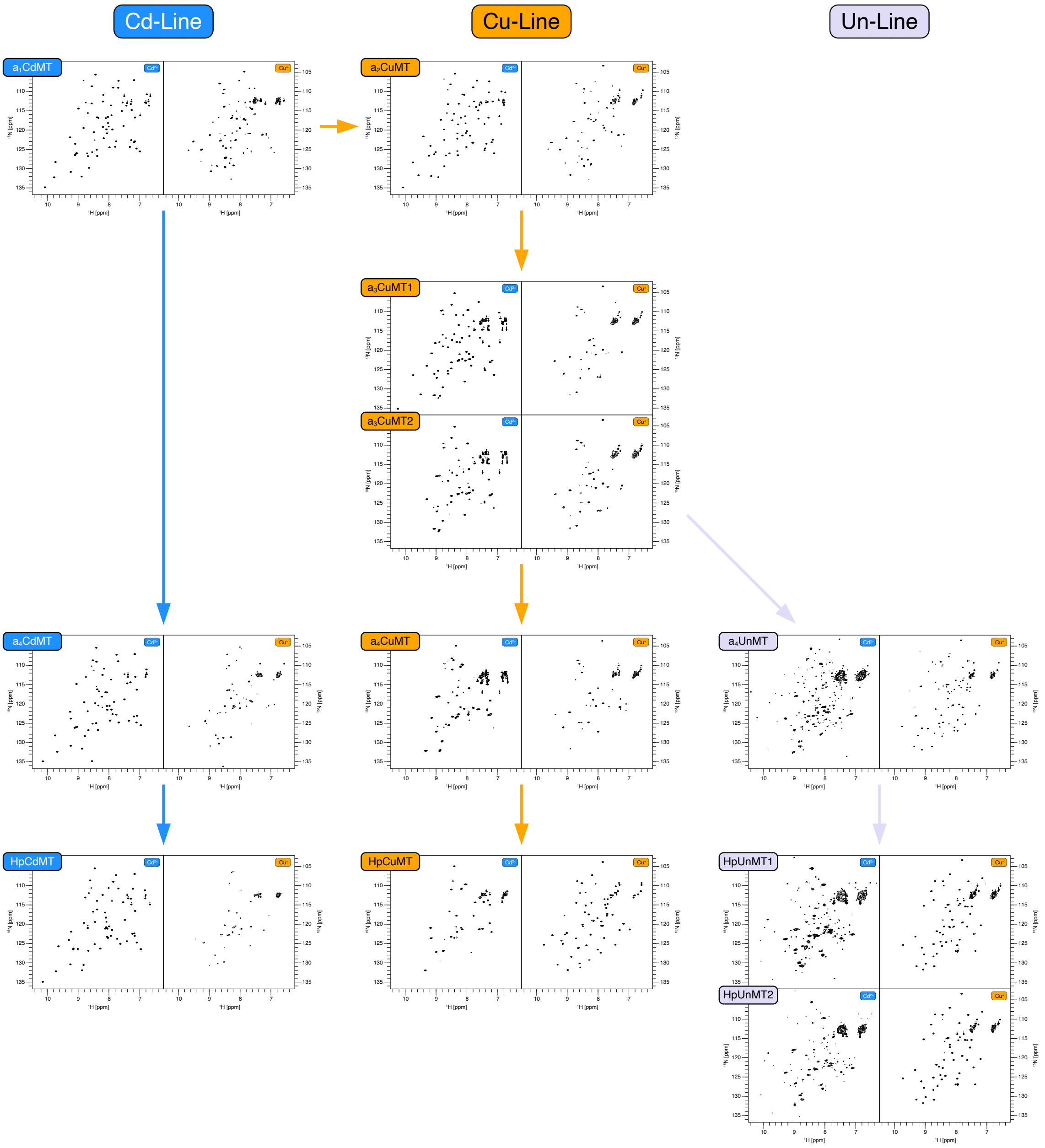

Fig. S3.1. [^{15}N,^{1}H]-HSQC spectra of extant HpMTs and reconstructed ancestral MTs. Small boxes inside the NMR spectra indicate the type of metalation. NMR spectra were recorded at 600 MHz and 298 K.

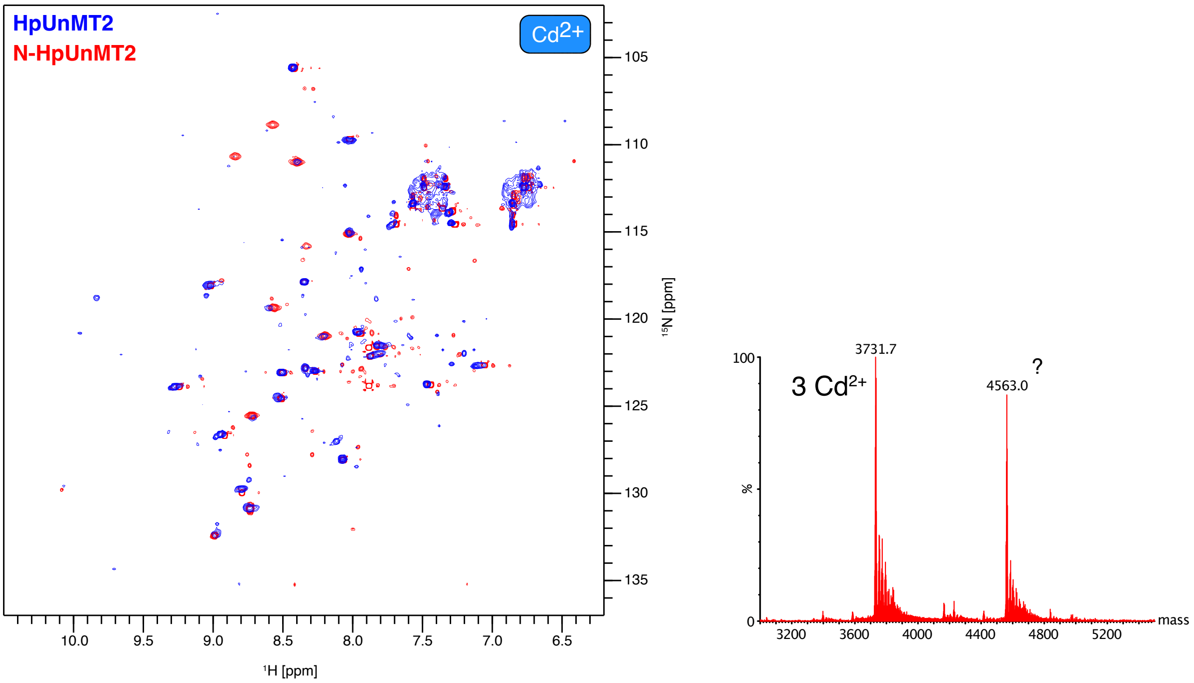

Fig. S3.2. [^{15}N,^{1}H]-HSQC spectra of Cd^{2+}-loaded HpUnMT2 (blue) and N^{domain}-HpUnMT2 (red) with ESI-MS data for Cd^{2+}-loaded N^{domain}-HpUnMT2 on the right. The mass spectrum contains an unidentified mass annotated with a question mark. Frequent overlap of peaks between the two constructs indicates that the strong peaks from Cd^{2+}-HpUnMT2 most likely stem only from the N-domain.

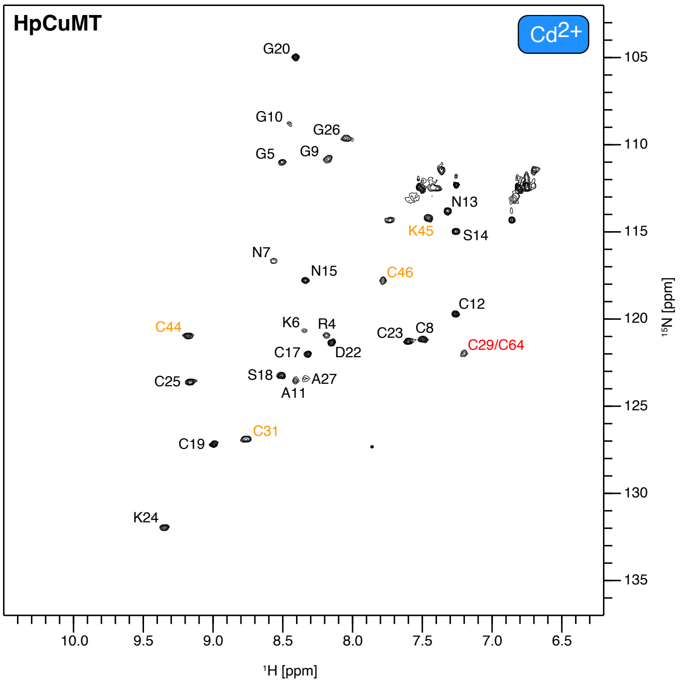

Fig. S3.3. [^{15}N,^{1}H]-HSQC spectrum of Cd^{2+}-loaded HpCuMT including backbone amide assignments as determined with triple resonance spectra. Orange color marks assignments where only a short sequence of residues (C44–C46) or just one residue without any of its neighbors (C31) were assigned. The resonance with the assignment in red (C29/C64) could not unambiguously be assigned to either residue. The assignment of peaks without any of its neighboring residues (C31, C29/C64) is inherently more ambiguous than assignments of sequentially linked residues.

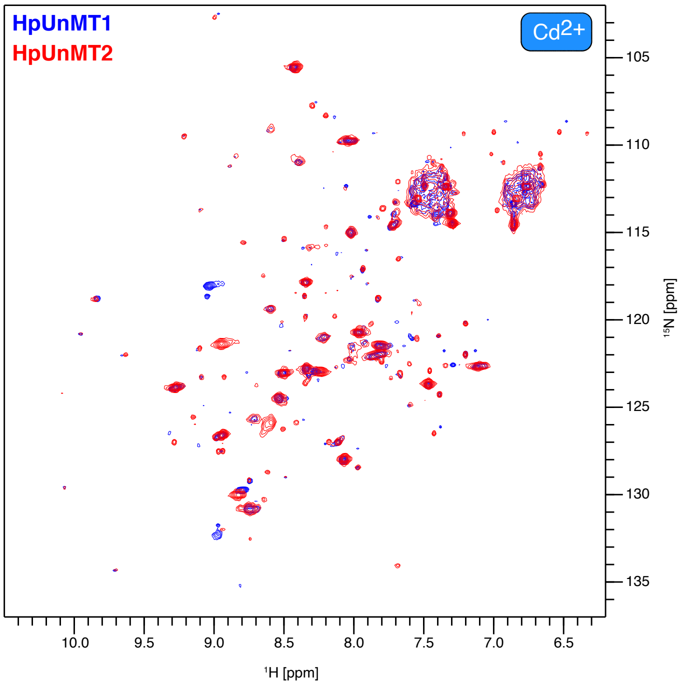

Fig. S3.4. [^{15}N,^{1}H]-HSQC spectra of Cd^{2+}-loaded HpUnMT1 (blue) and HpUnMT2 (red). The two variants differ in a single residue (HpUnMT1 with V32 and HpUnMT2 with A32). The prominent chemical shift change in the center left of the spectrum likely stems from this substitution, which therefore suggests that the strong peaks stem from the N-domain.

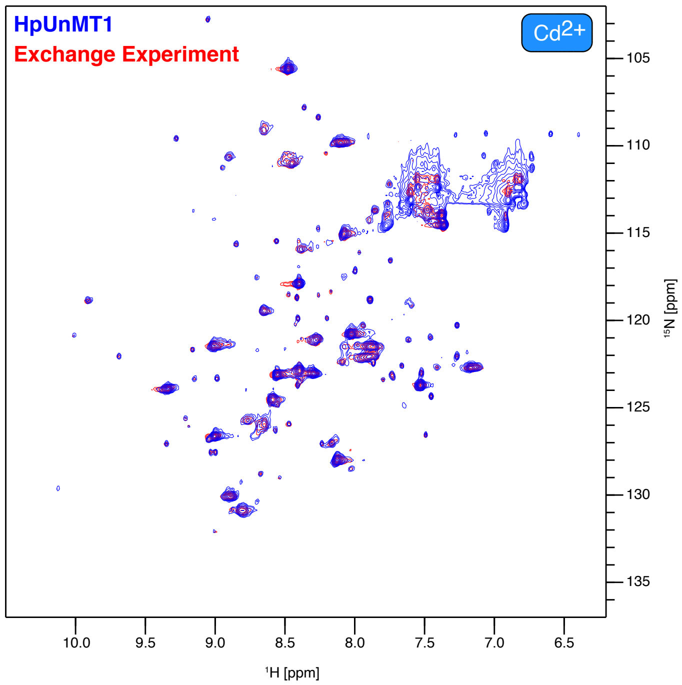

Fig. S3.5. [^{15}N,^{1}H]-HSQC (blue) and zz-^{15}N-HSQC spectrum (red) with a transfer delay τ of 400 ms of Cd^{2+}-HpUnMT1. The absence of additional peaks in the zz-^{15}N-HSQC spectrum indicates that no slow conformational exchange with lifetimes shorter than 100 ms is taking place.

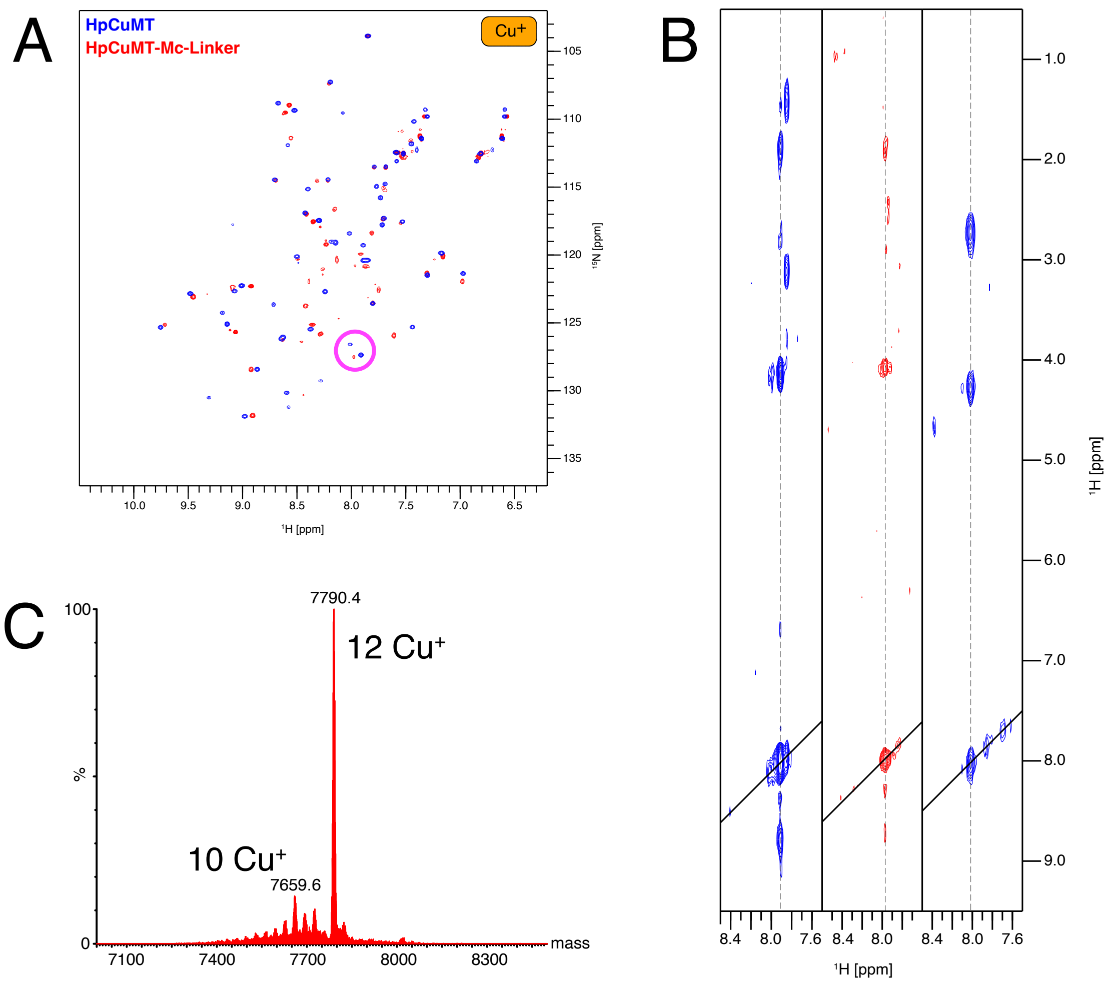

Fig. S3.6. (A) [^{15}N,^{1}H]-HSQC spectra of Cu^{+}-loaded HpCuMT (blue) and HpCuMT-Mc-Linker (red). Although some peak overlap is present, there are many chemical shift changes. **(B)** ^{15}N-TOCSY strips of Cu^{+}-loaded HpCuMT (blue) and HpCuMT-Mc-Linker (red) from the peaks in the pink circle in (A). Due to peak shifting, it is unclear, which of the blue peaks from HpCuMT corresponds to the red peak from HpCuMT-Mc-Linker. However, side chain proton resonances in the ^{15}N-TOCSY clearly indicate that it is the peak at around 7.9 ppm rather than around 8.0 ppm (on the ^{1}H axis) that corresponds to the peak from HpCuMT-Mc-Linker. Using such comparisons of TOCSY strips it is possible to identify the additional peaks from the longer linker allowing to exclude them from the ^{15}N{^{1}H}-NOE data analysis (Fig. S3.7). **(C)** ESI-MS data for Cu^{+}-loaded HpCuMT-Mc-Linker.

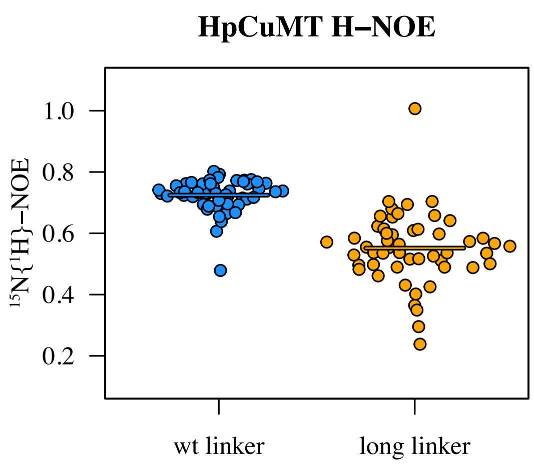

Fig. S3.7. $^{15}N\{^{1}H\}$-NOE ratios of HpCuMT with the 2-residue long wild-type (wt) and the 8-residue long *Megathura crenulata* (Mc) linker. The smaller $^{15}N\{^{1}H\}$-NOE ratios with the long linker indicate that the two putative domains have reduced rotational correlation times because they are motionally decoupled with the long linker.

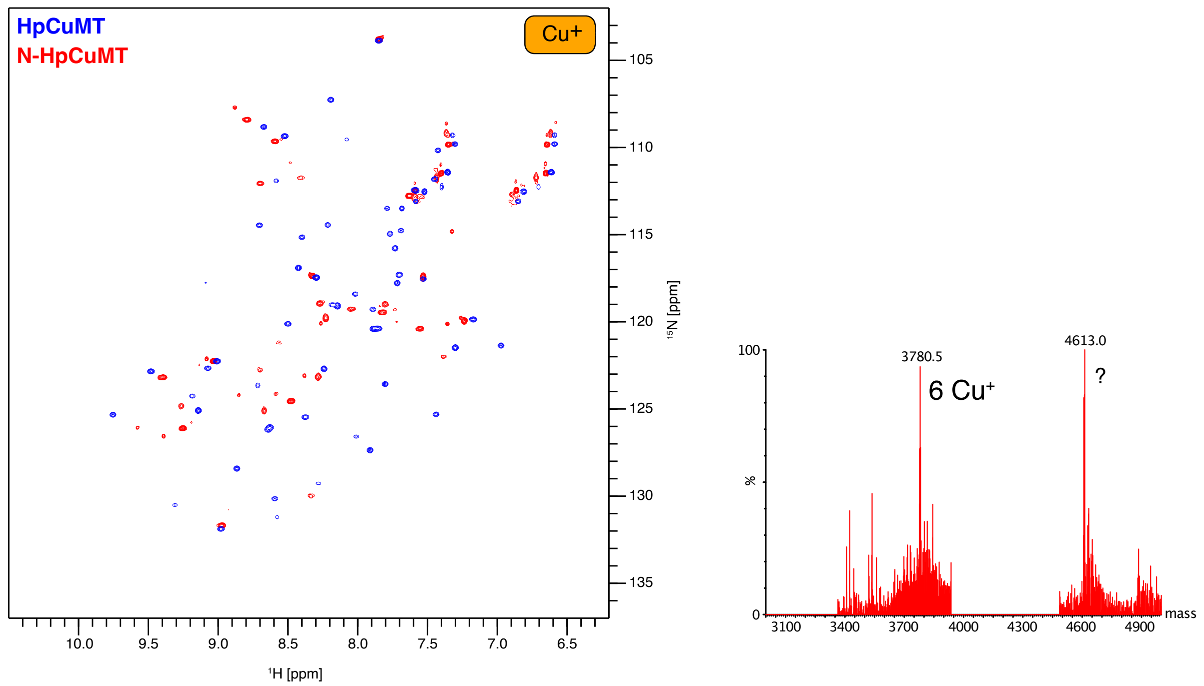

Fig. S3.8. [$^{15}N,^{1}H$]-HSQC spectra of Cu^{+}-loaded HpCuMT (blue) and N^{domain}-HpCuMT (red) with ESI-MS data for N^{domain}-HpCuMT on the right. The NMR spectrum indicates that the N-domain alone coordinates Cu^{+} ions in a defined fold, whereas the MS data shows that the N-domain alone binds 6 Cu^{+} ions in addition to some other species. Interestingly, the unidentified peak in the mass spectrum of Cd^{2+}-loaded N^{domain}-HpUnMT2 (Fig. S3.2) has a very similar mass indicating a complication in either the sample preparation or analysis connected to single domain constructs as it is not present with the full-length constructs.

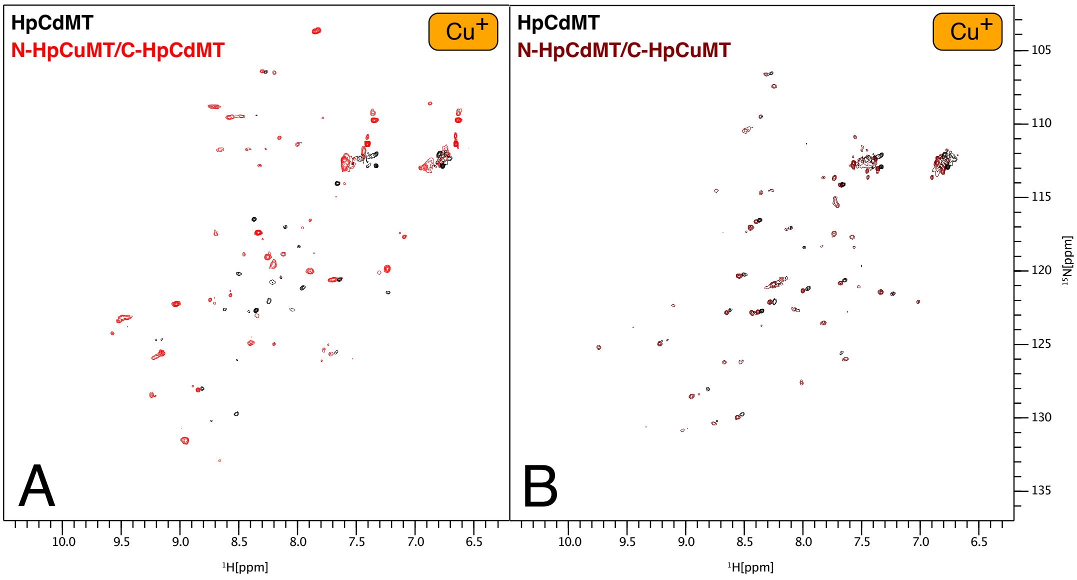

Fig. S3.9. [^{15}N,^{1}H]-HSQC spectrum of Cu^{+}-loaded HpCdMT (black), and two domain-chimeras: **(A)** N^{domain}-HpCuMT/C^{domain}-HpCdMT (red) and **(B)** N^{domain}-HpCdMT/C^{domain}-HpCuMT (brown). No peak overlap in A and almost complete overlap in B indicates that the observed peaks in the [^{15}N,^{1}H]-HSQC spectra of HpCdMT originate from its N-domain. Note that C-domain peaks of N^{domain}-HpCdMT/C^{domain}-HpCuMT are not very numerous or strong when compared to HpCuMT, suggesting that the N-domain of HpCuMT may have stabilizing effects on the C-domain.

References

1. R. Dallinger, O. Zerbe, C. Baumann, B. Egger, M. Capdevila, Ò. Palacios, R. Albalat, S. Calatayud, P. Ladurner, B. Schlick-Steiner, F. Steiner, V. Pedrini-Martha, R. Lackner, H. Lindner, M. Dvorak, M. Niederwanger, R. Schnegg and S. Atrian, Metallomics reveals a persisting impact of cadmium on the evolution of metal-selective snail metallothioneins, *Metallomics*, 2020, 12 (5), 702-720. https://doi.org/10.1039/c9mt00259f

2. O. Schicht and E. Freisinger, Spectroscopic characterization of *Cicer arietinum* metallothionein 1, *Inorg. Chim. Acta.*, 2009, 362 (3), 714-724. https://doi.org/10.1016/j.ica.2008.03.097
