# Supplementary material for: Evolution of Cd2+ and Cu+ binding in Helix pomatia metallothioneins
Source: Metallomics. 2023 Sep 20;15(10):mfad057. doi: 10.1093/mtomcs/mfad057 (PMC10548783; doi:10.1093/mtomcs/mfad057)
Supplement: mfad057_Supplemental_Files [file mfad057_supplemental_files.zip › suppl_data_2_CLEAN.pdf]

# Supplementary Data 2

**Evolution of $Cd^{2+}$ and $Cu^{+}$ binding in *Helix pomatia* metallothioneins**

Renato Valsecchi*, Christian Baumann*, Ardit Lila, Oliver Zerbe

**Index**


| | Peak Intensities | | T₂ Relaxation Times: | |
|---|---|---|---|---|
| $Cd^{2+}$-$a_1$CdMT | Table S1 | p. 2 | Table S23 | p. 12 |
| $Cd^{2+}$-$a_2$CuMT | Table S2 | p. 2 | Table S24 | p. 13 |
| $Cd^{2+}$-$a_3$CuMT1 | Table S3 | p. 3 | Table S25 | p. 14 |
| $Cd^{2+}$-$a_3$CuMT2 | Table S4 | p. 3 | Table S26 | p. 15 |
| $Cd^{2+}$-$a_4$CdMT | Table S5 | p. 4 | Table S27 | p. 16 |
| $Cd^{2+}$-$a_4$CuMT | Table S6 | p. 4 | Table S28 | p. 17 |
| $Cd^{2+}$-$a_4$UnMT | Table S7 | p. 5 | Table S29 | p. 18 |
| $Cd^{2+}$-HpCdMT* | Table S8* | p. 5 | Table S30* | p. 20 |
| $Cd^{2+}$-HpCuMT | Table S9 | p. 6 | Table S31 | p. 21 |
| $Cd^{2+}$-HpUnMT1 | Table S10 | p. 6 | Table S32 | p. 22 |
| $Cd^{2+}$-HpUnMT2 | Table S11 | p. 7 | Table S33 | p. 23 |
| $Cu^{+}$-$a_1$CdMT | Table S12 | p. 7 | Table S34 | p. 25 |
| $Cu^{+}$-$a_2$CuMT | Table S13 | p. 8 | Table S35 | p. 26 |
| $Cu^{+}$-$a_3$CuMT1 | Table S14 | p. 8 | Table S36 | p. 27 |
| $Cu^{+}$-$a_3$CuMT2 | Table S15 | p. 8 | Table S37 | p. 28 |
| $Cu^{+}$-$a_4$CdMT | Table S16 | p. 9 | Table S38 | p. 29 |
| $Cu^{+}$-$a_4$CuMT | Table S17 | p. 9 | Table S39 | p. 30 |
| $Cu^{+}$-$a_4$UnMT | Table S18 | p. 9 | Table S40 | p. 31 |
| $Cu^{+}$-HpCdMT | Table S19 | p. 10 | Table S41 | p. 32 |
| $Cu^{+}$-HpCuMT | Table S20 | p. 10 | Table S42 | p. 33 |
| $Cu^{+}$-HpUnMT1 | Table S21 | p. 11 | Table S43 | p. 34 |
| $Cu^{+}$-HpUnMT2 | Table S22 | p. 11 | Table S44 | p. 35 |


* The same data was previously used by us to calculate $T_2$ relaxation times in Beil A, Jurt S, Walser R et al. *The Solution Structure and Dynamics of Cd-Metallothionein from Helix pomatia Reveal Optimization for Binding Cd over Zn, Biochemistry, 2019, 58 (45), 4570-4581. https://doi.org/10.1021/acs.biochem.9b00830.* We refitted the data here to calculate the $T_2$ error based on the same Monte Carlo approach used for the newly recorded $T_2$ data.

**Table S1.** [$^{15}$N,$^{1}$H]-HSQC peak intensities of **a$_1$CdMT** (N20) binding **Cd$^{2+}$.**

| | Peak Intensity |
|---|---|
| **1** | 1.810E+10 |
| **2** | 1.577E+10 |
| **3** | 1.876E+10 |
| **4** | 1.912E+10 |
| **5** | 1.964E+10 |
| **6** | 1.871E+10 |
| **7** | 1.641E+10 |
| **8** | 1.733E+10 |
| **9** | 1.579E+10 |
| **10** | 1.768E+10 |
| **11** | 1.632E+10 |
| **12** | 1.718E+10 |
| **13** | 1.614E+10 |
| **14** | 1.342E+10 |
| **15** | 1.737E+10 |
| **16** | 1.626E+10 |
| **17** | 1.422E+10 |
| **18** | 1.368E+10 |
| **19** | 1.648E+10 |
| **20** | 1.431E+10 |
| **21** | 1.650E+10 |
| **22** | 1.661E+10 |
| **23** | 1.639E+10 |
| **24** | 1.186E+10 |
| **25** | 1.281E+10 |
| **26** | 1.561E+10 |
| **27** | 1.140E+10 |
| **28** | 1.433E+10 |
| **29** | 1.647E+10 |
| **30** | 1.246E+10 |
| **31** | 1.310E+10 |
| **32** | 1.173E+10 |
| **33** | 1.275E+10 |
| **34** | 9.677E+09 |
| **35** | 1.449E+10 |
| **36** | 1.327E+10 |
| **37** | 1.356E+10 |
| **38** | 1.178E+10 |
| **39** | 1.291E+10 |
| **40** | 1.268E+10 |
| **41** | 1.164E+10 |
| **42** | 1.153E+10 |
| **43** | 1.128E+10 |
| **44** | 9.442E+09 |
| **45** | 9.931E+09 |
| **46** | 8.757E+09 |
| **47** | 9.018E+09 |
| **48** | 9.741E+09 |
| **49** | 8.544E+09 |
| **50** | 7.206E+09 |
| **51** | 6.389E+09 |
| **52** | 5.380E+09 |
| **53** | 4.442E+09 |
| **54** | 5.001E+09 |
| **55** | 3.193E+09 |
| **56** | 3.152E+09 |
| **57** | 2.370E+09 |
| **58** | 1.991E+09 |
| **59** | 1.802E+09 |
| **60** | 1.058E+09 |

**Table S2.** [$^{15}$N,$^{1}$H]-HSQC peak intensities of **a$_2$CuMT** (N36) binding **Cd$^{2+}$.**

| | Peak Intensity |
|---|---|
| **1** | 3.822E+09 |
| **2** | 4.256E+09 |
| **3** | 3.937E+09 |
| **4** | 4.007E+09 |
| **5** | 3.587E+09 |
| **6** | 3.552E+09 |
| **7** | 3.551E+09 |
| **8** | 2.782E+09 |
| **9** | 3.217E+09 |
| **10** | 3.207E+09 |
| **11** | 3.248E+09 |
| **12** | 2.142E+09 |
| **13** | 2.192E+09 |
| **14** | 2.427E+09 |
| **15** | 2.719E+09 |
| **16** | 2.858E+09 |
| **17** | 2.000E+09 |
| **18** | 2.496E+09 |
| **19** | 2.753E+09 |
| **20** | 2.914E+09 |
| **21** | 2.437E+09 |
| **22** | 3.004E+09 |
| **23** | 1.978E+09 |
| **24** | 2.210E+09 |
| **25** | 2.603E+09 |
| **26** | 1.725E+09 |
| **27** | 2.435E+09 |
| **28** | 1.691E+09 |
| **29** | 2.416E+09 |
| **30** | 2.382E+09 |
| **31** | 2.333E+09 |
| **32** | 1.531E+09 |
| **33** | 2.150E+09 |
| **34** | 2.442E+09 |
| **35** | 1.625E+09 |
| **36** | 2.177E+09 |
| **37** | 1.375E+09 |
| **38** | 1.254E+09 |
| **39** | 1.867E+09 |
| **40** | 1.465E+09 |
| **41** | 1.912E+09 |
| **42** | 1.814E+09 |
| **43** | 1.701E+09 |
| **44** | 1.090E+09 |
| **45** | 1.705E+09 |
| **46** | 1.580E+09 |
| **47** | 1.109E+09 |
| **48** | 1.069E+09 |
| **49** | 1.452E+09 |
| **50** | 1.465E+09 |
| **51** | 1.281E+09 |
| **52** | 1.112E+09 |
| **53** | 7.375E+08 |
| **54** | 8.583E+08 |
| **55** | 8.967E+08 |
| **56** | 6.159E+08 |
| **57** | 7.286E+08 |
| **58** | 4.624E+08 |
| **59** | 4.763E+08 |
| **60** | 2.362E+08 |

**Table S3.** [$^{15}$N,$^{1}$H]-HSQC peak intensities of **$a_3$CuMT1** (N40) binding **$Cd^{2+}$**.

| | Peak Intensity |
|---|---|
| **1** | 8.067E+10 |
| **2** | 8.507E+10 |
| **3** | 8.917E+10 |
| **4** | 8.556E+10 |
| **5** | 8.740E+10 |
| **6** | 7.668E+10 |
| **7** | 7.102E+10 |
| **8** | 6.563E+10 |
| **9** | 6.808E+10 |
| **10** | 5.341E+10 |
| **11** | 5.567E+10 |
| **12** | 5.239E+10 |
| **13** | 4.617E+10 |
| **14** | 4.286E+10 |
| **15** | 4.493E+10 |
| **16** | 3.961E+10 |
| **17** | 3.951E+10 |
| **18** | 4.221E+10 |
| **19** | 4.212E+10 |
| **20** | 3.551E+10 |
| **21** | 3.021E+10 |
| **22** | 2.915E+10 |
| **23** | 2.534E+10 |
| **24** | 2.230E+10 |
| **25** | 2.111E+10 |
| **26** | 2.213E+10 |
| **27** | 2.013E+10 |
| **28** | 2.010E+10 |
| **29** | 1.910E+10 |
| **30** | 1.871E+10 |
| **31** | 1.866E+10 |
| **32** | 1.618E+10 |
| **33** | 1.545E+10 |
| **34** | 1.506E+10 |
| **35** | 1.589E+10 |
| **36** | 1.431E+10 |
| **37** | 1.361E+10 |
| **38** | 1.266E+10 |
| **39** | 1.192E+10 |
| **40** | 1.169E+10 |
| **41** | 1.065E+10 |
| **42** | 1.032E+10 |
| **43** | 1.014E+10 |
| **44** | 1.043E+10 |
| **45** | 9.410E+09 |
| **46** | 8.713E+09 |
| **47** | 7.839E+09 |
| **48** | 7.430E+09 |
| **49** | 7.491E+09 |
| **50** | 7.211E+09 |
| **51** | 7.193E+09 |
| **52** | 6.873E+09 |
| **53** | 6.611E+09 |
| **54** | 6.366E+09 |
| **55** | 5.748E+09 |
| **56** | 5.239E+09 |
| **57** | 5.331E+09 |
| **58** | 4.693E+09 |
| **59** | 4.065E+09 |
| **60** | 3.843E+09 |
| **61** | 3.601E+09 |
| **62** | 3.343E+09 |
| **63** | 2.689E+09 |

**Table S4.** [$^{15}$N,$^{1}$H]-HSQC peak intensities of **$a_3$CuMT2** (N40 G43S) binding **$Cd^{2+}$**.

| | Peak Intensity |
|---|---|
| **1** | 9.499E+10 |
| **2** | 5.485E+10 |
| **3** | 6.533E+10 |
| **4** | 5.478E+10 |
| **5** | 4.938E+10 |
| **6** | 5.554E+10 |
| **7** | 3.592E+10 |
| **8** | 4.267E+10 |
| **9** | 3.179E+10 |
| **10** | 4.285E+10 |
| **11** | 3.848E+10 |
| **12** | 2.664E+10 |
| **13** | 3.568E+10 |
| **14** | 2.765E+10 |
| **15** | 2.954E+10 |
| **16** | 2.775E+10 |
| **17** | 2.574E+10 |
| **18** | 2.014E+10 |
| **19** | 2.243E+10 |
| **20** | 1.902E+10 |
| **21** | 1.886E+10 |
| **22** | 1.485E+10 |
| **23** | 1.100E+10 |
| **24** | 7.708E+09 |
| **25** | 6.942E+09 |
| **26** | 7.028E+09 |
| **27** | 7.215E+09 |
| **28** | 5.639E+09 |
| **29** | 4.075E+09 |
| **30** | 3.586E+09 |
| **31** | 3.037E+09 |
| **32** | 3.121E+09 |
| **33** | 3.153E+09 |
| **34** | 3.356E+09 |
| **35** | 2.615E+09 |
| **36** | 2.288E+09 |
| **37** | 2.253E+09 |
| **38** | 2.448E+09 |
| **39** | 2.225E+09 |
| **40** | 2.772E+09 |
| **41** | 2.509E+09 |
| **42** | 2.157E+09 |
| **43** | 2.128E+09 |
| **44** | 1.445E+09 |
| **45** | 1.540E+09 |
| **46** | 1.372E+09 |
| **47** | 1.463E+09 |
| **48** | 1.326E+09 |
| **49** | 1.028E+09 |
| **50** | 1.266E+09 |
| **51** | 9.001E+08 |
| **52** | 1.154E+09 |
| **53** | 1.179E+09 |
| **54** | 1.127E+09 |
| **55** | 1.156E+09 |
| **56** | 1.191E+09 |
| **57** | 1.029E+09 |

**Table S5.** [$^{15}$N,$^{1}$H]-HSQC peak intensities of **a$_4$CdMT** (N28) binding **Cd$^{2+}$**.

| | Peak Intensity |
|---|---|
| **1** | 1.933E+10 |
| **2** | 1.693E+10 |
| **3** | 1.718E+10 |
| **4** | 1.366E+10 |
| **5** | 1.491E+10 |
| **6** | 1.505E+10 |
| **7** | 1.497E+10 |
| **8** | 1.108E+10 |
| **9** | 1.549E+10 |
| **10** | 1.163E+10 |
| **11** | 1.424E+10 |
| **12** | 1.094E+10 |
| **13** | 1.395E+10 |
| **14** | 1.381E+10 |
| **15** | 1.328E+10 |
| **16** | 1.314E+10 |
| **17** | 1.472E+10 |
| **18** | 1.259E+10 |
| **19** | 1.135E+10 |
| **20** | 1.268E+10 |
| **21** | 1.226E+10 |
| **22** | 1.144E+10 |
| **23** | 1.298E+10 |
| **24** | 9.744E+09 |
| **25** | 1.172E+10 |
| **26** | 1.307E+10 |
| **27** | 1.085E+10 |
| **28** | 1.208E+10 |
| **29** | 9.567E+09 |
| **30** | 1.195E+10 |
| **31** | 8.889E+09 |
| **32** | 1.194E+10 |
| **33** | 8.613E+09 |
| **34** | 9.442E+09 |
| **35** | 8.088E+09 |
| **36** | 1.078E+10 |
| **37** | 1.035E+10 |
| **38** | 9.627E+09 |
| **39** | 8.296E+09 |
| **40** | 7.088E+09 |
| **41** | 7.209E+09 |
| **42** | 6.858E+09 |
| **43** | 6.912E+09 |
| **44** | 5.447E+09 |
| **45** | 6.768E+09 |
| **46** | 4.714E+09 |
| **47** | 5.974E+09 |
| **48** | 5.429E+09 |
| **49** | 5.322E+09 |
| **50** | 6.770E+09 |
| **51** | 5.329E+09 |
| **52** | 4.351E+09 |
| **53** | 3.232E+09 |
| **54** | 3.151E+09 |
| **55** | 3.151E+09 |
| **56** | 2.614E+09 |
| **57** | 1.087E+09 |
| **58** | 1.022E+09 |
| **59** | 8.016E+08 |
| **60** | 6.819E+08 |

**Table S6.** [$^{15}$N,$^{1}$H]-HSQC peak intensities of **a$_4$CuMT** (N46) binding **Cd$^{2+}$**.

| | Peak Intensity |
|---|---|
| **1** | 5.034E+10 |
| **2** | 3.169E+09 |
| **3** | 5.267E+09 |
| **4** | 1.522E+09 |
| **5** | 3.840E+09 |
| **6** | 2.206E+10 |
| **7** | 2.894E+10 |
| **8** | 1.545E+10 |
| **9** | 1.623E+09 |
| **10** | 9.928E+08 |
| **11** | 2.542E+10 |
| **12** | 1.319E+09 |
| **13** | 9.467E+08 |
| **14** | 2.892E+10 |
| **15** | 2.156E+09 |
| **16** | 1.772E+10 |
| **17** | 4.821E+09 |
| **18** | 2.826E+10 |
| **19** | 1.855E+10 |
| **20** | 8.564E+09 |
| **21** | 1.302E+09 |
| **22** | 2.975E+09 |
| **23** | 9.866E+09 |
| **24** | 3.819E+09 |
| **25** | 1.645E+10 |
| **26** | 6.382E+09 |
| **27** | 1.176E+09 |
| **28** | 8.515E+09 |
| **29** | 1.465E+09 |
| **30** | 3.407E+10 |
| **31** | 2.576E+10 |
| **32** | 5.118E+10 |
| **33** | 2.370E+09 |
| **34** | 6.138E+10 |
| **35** | 3.318E+10 |
| **36** | 1.365E+10 |
| **37** | 2.405E+10 |
| **38** | 5.110E+10 |
| **39** | 4.549E+10 |
| **40** | 1.749E+09 |
| **41** | 2.348E+10 |
| **42** | 2.640E+10 |
| **43** | 1.206E+10 |
| **44** | 1.864E+10 |

**Table S7.** [$^{15}$N,$^{1}$H]-HSQC peak intensities of **a$_4$UnMT** (N41) binding **Cd$^{2+}$**.

| | Peak Intensity |
|---|---|
| **1** | 5.516E+08 |
| **2** | 2.733E+09 |
| **3** | 3.231E+08 |
| **4** | 1.072E+09 |
| **5** | 2.968E+08 |
| **6** | 5.039E+08 |
| **7** | 5.342E+08 |
| **8** | 4.102E+08 |
| **9** | 3.432E+08 |
| **10** | 6.606E+08 |
| **11** | 4.209E+08 |
| **12** | 4.796E+08 |
| **13** | 5.378E+08 |
| **14** | 8.515E+08 |
| **15** | 2.066E+09 |
| **16** | 3.463E+08 |
| **17** | 3.804E+08 |
| **18** | 6.051E+08 |
| **19** | 3.511E+08 |
| **20** | 3.594E+08 |
| **21** | 6.322E+08 |
| **22** | 4.401E+08 |
| **23** | 3.558E+08 |
| **24** | 2.931E+09 |
| **25** | 6.793E+08 |
| **26** | 3.137E+08 |
| **27** | 5.683E+08 |
| **28** | 1.025E+09 |
| **29** | 4.132E+08 |
| **30** | 3.427E+08 |
| **31** | 4.812E+08 |
| **32** | 3.411E+08 |
| **33** | 3.929E+08 |
| **34** | 5.292E+08 |
| **35** | 4.658E+08 |
| **36** | 3.750E+08 |
| **37** | 3.521E+08 |
| **38** | 5.516E+08 |
| **39** | 7.219E+08 |
| **40** | 6.976E+08 |
| **41** | 3.275E+09 |
| **42** | 1.055E+09 |
| **43** | 8.367E+08 |
| **44** | 3.842E+08 |
| **45** | 3.594E+08 |
| **46** | 4.111E+08 |
| **47** | 3.116E+08 |
| **48** | 4.545E+08 |
| **49** | 5.550E+08 |
| **50** | 1.480E+09 |
| **51** | 3.992E+08 |
| **52** | 1.339E+09 |
| **53** | 3.668E+08 |
| **54** | 6.823E+08 |
| **55** | 4.299E+08 |
| **56** | 2.564E+09 |
| **57** | 3.333E+08 |
| **58** | 1.839E+09 |
| **59** | 3.649E+08 |
| **60** | 1.921E+09 |
| **61** | 5.317E+08 |
| **62** | 7.623E+08 |
| **63** | 9.742E+08 |
| **64** | 9.091E+08 |
| **65** | 1.584E+09 |
| **66** | 5.861E+08 |
| **67** | 3.074E+08 |
| **68** | 2.941E+08 |
| **69** | 6.142E+08 |
| **70** | 4.444E+08 |
| **71** | 4.066E+08 |
| **72** | 3.170E+08 |
| **73** | 3.933E+08 |

**Table S8.** [$^{15}$N,$^{1}$H]-HSQC peak intensities of **HpCdMT** binding **Cd$^{2+}$**.

| | Peak Intensity |
|---|---|
| **1** | 8.901E+09 |
| **2** | 8.405E+09 |
| **3** | 6.174E+09 |
| **4** | 8.764E+09 |
| **5** | 7.258E+09 |
| **6** | 8.260E+09 |
| **7** | 7.738E+09 |
| **8** | 8.023E+09 |
| **9** | 5.402E+09 |
| **10** | 6.926E+09 |
| **11** | 5.624E+09 |
| **12** | 6.928E+09 |
| **13** | 6.337E+09 |
| **14** | 6.918E+09 |
| **15** | 4.195E+09 |
| **16** | 6.538E+09 |
| **17** | 5.037E+09 |
| **18** | 6.434E+09 |
| **19** | 4.670E+09 |
| **20** | 5.534E+09 |
| **21** | 4.113E+09 |
| **22** | 4.987E+09 |
| **23** | 6.399E+09 |
| **24** | 5.321E+09 |
| **25** | 3.339E+09 |
| **26** | 4.263E+09 |
| **27** | 5.079E+09 |
| **28** | 4.472E+09 |
| **29** | 4.127E+09 |
| **30** | 4.488E+09 |
| **31** | 4.355E+09 |
| **32** | 4.757E+09 |
| **33** | 6.250E+09 |
| **34** | 4.952E+09 |
| **35** | 3.557E+09 |
| **36** | 5.787E+09 |
| **37** | 4.409E+09 |
| **38** | 3.451E+09 |
| **39** | 3.722E+09 |
| **40** | 4.729E+09 |
| **41** | 4.733E+09 |
| **42** | 2.642E+09 |
| **43** | 4.024E+09 |
| **44** | 3.043E+09 |
| **45** | 3.147E+09 |
| **46** | 2.929E+09 |
| **47** | 3.909E+09 |
| **48** | 3.546E+09 |
| **49** | 3.385E+09 |
| **50** | 3.329E+09 |
| **51** | 2.825E+09 |
| **52** | 2.790E+09 |
| **53** | 1.587E+09 |
| **54** | 1.732E+09 |
| **55** | 1.621E+09 |
| **56** | 1.217E+09 |
| **57** | 1.149E+09 |
| **58** | 7.288E+08 |
| **59** | 2.050E+08 |

**Table S9.** [$^{15}$N,$^{1}$H]-HSQC peak intensities of **HpCuMT** binding **Cd$^{2+}$**.

| | Peak Intensity |
|---|---|
| **1** | 1.544E+10 |
| **2** | 1.473E+10 |
| **3** | 1.291E+10 |
| **4** | 9.099E+09 |
| **5** | 1.196E+10 |
| **6** | 1.233E+10 |
| **7** | 1.231E+10 |
| **8** | 7.787E+09 |
| **9** | 8.485E+09 |
| **10** | 8.135E+09 |
| **11** | 8.549E+09 |
| **12** | 6.612E+09 |
| **13** | 6.207E+09 |
| **14** | 7.117E+09 |
| **15** | 6.230E+09 |
| **16** | 4.394E+09 |
| **17** | 3.418E+09 |
| **18** | 2.870E+09 |
| **19** | 2.718E+09 |
| **20** | 3.413E+09 |
| **21** | 2.309E+09 |
| **22** | 2.357E+09 |
| **23** | 1.973E+09 |
| **24** | 1.373E+09 |
| **25** | 1.122E+09 |
| **26** | 1.145E+09 |
| **27** | 1.140E+09 |

**Table S10.** [$^{15}$N,$^{1}$H]-HSQC peak intensities of **HpUnMT1** binding **Cd$^{2+}$**.

| | Peak Intensity |
|---|---|
| **1** | 1.394E+09 |
| **2** | 1.743E+09 |
| **3** | 1.958E+09 |
| **4** | 1.433E+09 |
| **5** | 2.640E+09 |
| **6** | 1.591E+09 |
| **7** | 1.700E+09 |
| **8** | 1.909E+09 |
| **9** | 1.542E+10 |
| **10** | 1.465E+09 |
| **11** | 4.302E+09 |
| **12** | 1.642E+09 |
| **13** | 1.721E+10 |
| **14** | 3.474E+09 |
| **15** | 1.732E+09 |
| **16** | 2.986E+09 |
| **17** | 6.436E+09 |
| **18** | 5.476E+09 |
| **19** | 1.309E+10 |
| **20** | 1.340E+10 |
| **21** | 2.365E+09 |
| **22** | 2.425E+10 |
| **23** | 1.846E+10 |
| **24** | 1.562E+10 |
| **25** | 2.493E+09 |
| **26** | 1.666E+09 |
| **27** | 2.049E+09 |
| **28** | 9.350E+09 |
| **29** | 1.832E+10 |
| **30** | 1.366E+09 |
| **31** | 2.565E+09 |
| **32** | 1.490E+09 |
| **33** | 2.247E+09 |
| **34** | 9.366E+09 |
| **35** | 1.866E+09 |
| **36** | 1.334E+09 |
| **37** | 1.663E+09 |
| **38** | 2.433E+09 |
| **39** | 1.301E+10 |
| **40** | 1.430E+09 |
| **41** | 1.401E+09 |
| **42** | 2.499E+09 |
| **43** | 1.537E+09 |
| **44** | 2.352E+09 |
| **45** | 1.882E+09 |
| **46** | 9.328E+09 |
| **47** | 1.733E+09 |
| **48** | 1.503E+09 |
| **49** | 1.643E+09 |
| **50** | 2.758E+09 |
| **51** | 2.021E+09 |
| **52** | 2.369E+09 |
| **53** | 8.259E+09 |
| **54** | 2.031E+09 |
| **55** | 1.567E+09 |
| **56** | 2.628E+09 |
| **57** | 2.032E+09 |
| **58** | 1.677E+09 |
| **59** | 2.318E+10 |
| **60** | 3.183E+09 |
| **61** | 1.310E+10 |
| **62** | 1.607E+10 |
| **63** | 3.136E+09 |
| **64** | 2.184E+09 |
| **65** | 1.412E+09 |
| **66** | 6.335E+09 |
| **67** | 1.793E+09 |
| **68** | 1.933E+09 |
| **69** | 2.943E+10 |
| **70** | 8.636E+09 |

**Table S11.** [$^{15}$N,$^{1}$H]-HSQC peak intensities of **HpUnMT2** binding **$Cd^{2+}$**.

| | Peak Intensity |
|---|---|
| **1** | 3.013E+10 |
| **2** | 1.982E+10 |
| **3** | 2.476E+09 |
| **4** | 1.130E+09 |
| **5** | 1.112E+10 |
| **6** | 1.176E+09 |
| **7** | 1.020E+09 |
| **8** | 9.991E+08 |
| **9** | 1.107E+09 |
| **10** | 6.233E+09 |
| **11** | 1.063E+09 |
| **12** | 1.030E+09 |
| **13** | 9.955E+08 |
| **14** | 9.663E+08 |
| **15** | 9.791E+08 |
| **16** | 1.464E+09 |
| **17** | 1.549E+09 |
| **18** | 1.209E+09 |
| **19** | 9.435E+08 |
| **20** | 1.066E+09 |
| **21** | 7.926E+08 |
| **22** | 1.316E+09 |
| **23** | 2.057E+10 |
| **24** | 1.125E+09 |
| **25** | 9.027E+08 |
| **26** | 1.456E+10 |
| **27** | 3.403E+09 |
| **28** | 2.204E+09 |
| **29** | 3.159E+09 |
| **30** | 8.454E+08 |
| **31** | 1.583E+09 |
| **32** | 1.138E+10 |
| **33** | 9.410E+08 |
| **34** | 2.091E+09 |
| **35** | 3.059E+09 |
| **36** | 8.512E+08 |
| **37** | 8.907E+08 |
| **38** | 2.552E+10 |
| **39** | 1.291E+10 |
| **40** | 2.666E+09 |
| **41** | 1.413E+10 |
| **42** | 8.342E+09 |
| **43** | 1.370E+09 |
| **44** | 1.540E+09 |
| **45** | 9.902E+08 |
| **46** | 1.460E+09 |
| **47** | 1.834E+10 |
| **48** | 4.412E+09 |
| **49** | 1.358E+09 |
| **50** | 1.186E+09 |
| **51** | 2.885E+09 |
| **52** | 5.382E+09 |
| **53** | 2.072E+09 |
| **54** | 1.031E+10 |
| **55** | 2.426E+09 |
| **56** | 1.128E+09 |
| **57** | 7.884E+08 |
| **58** | 9.252E+08 |
| **59** | 9.552E+09 |
| **60** | 6.048E+09 |
| **61** | 1.771E+10 |
| **62** | 9.753E+08 |
| **63** | 1.663E+10 |
| **64** | 1.066E+09 |
| **65** | 8.704E+09 |
| **66** | 1.821E+09 |
| **67** | 1.249E+09 |
| **68** | 1.599E+10 |
| **69** | 3.240E+09 |
| **70** | 1.033E+09 |

**Table S12.** [$^{15}$N,$^{1}$H]-HSQC peak intensities of **$a_1$CdMT** (N20) binding **$Cu^{+}$**.

| | Peak Intensity |
|---|---|
| **1** | 1.061E+09 |
| **2** | 4.838E+09 |
| **3** | 7.358E+08 |
| **4** | 1.248E+09 |
| **5** | 3.146E+09 |
| **6** | 7.040E+08 |
| **7** | 4.462E+08 |
| **8** | 5.970E+09 |
| **9** | 7.219E+08 |
| **10** | 7.709E+08 |
| **11** | 7.862E+08 |
| **12** | 1.144E+09 |
| **13** | 4.381E+08 |
| **14** | 1.935E+09 |
| **15** | 6.064E+08 |
| **16** | 1.199E+09 |
| **17** | 5.141E+08 |
| **18** | 5.011E+09 |
| **19** | 1.682E+09 |
| **20** | 4.370E+08 |
| **21** | 4.739E+08 |
| **22** | 7.802E+08 |
| **23** | 1.865E+09 |
| **24** | 2.480E+09 |
| **25** | 3.756E+08 |
| **26** | 1.108E+09 |
| **27** | 5.797E+08 |
| **28** | 1.624E+09 |
| **29** | 1.072E+09 |
| **30** | 2.220E+09 |
| **31** | 2.458E+09 |
| **32** | 9.205E+08 |
| **33** | 1.495E+09 |
| **34** | 8.231E+09 |
| **35** | 3.481E+08 |
| **36** | 4.445E+09 |
| **37** | 6.756E+08 |
| **38** | 6.402E+09 |
| **39** | 4.236E+09 |
| **40** | 2.376E+09 |
| **41** | 8.135E+08 |
| **42** | 4.967E+08 |
| **43** | 8.942E+08 |
| **44** | 1.550E+09 |
| **45** | 5.414E+08 |
| **46** | 5.055E+08 |
| **47** | 1.142E+09 |
| **48** | 4.671E+09 |
| **49** | 6.113E+08 |
| **50** | 3.787E+09 |
| **51** | 3.728E+08 |
| **52** | 1.391E+09 |
| **53** | 8.657E+08 |
| **54** | 2.391E+09 |
| **55** | 7.598E+08 |
| **56** | 4.585E+08 |
| **57** | 3.104E+09 |
| **58** | 3.852E+08 |
| **59** | 4.254E+08 |

**Table S13.** [$^{15}$N,$^{1}$H]-HSQC peak intensities of **a$_2$CuMT** (N36) binding **Cu$^+$.**

| | Peak Intensity |
|---|---|
| **1** | 1.126E+08 |
| **2** | 1.225E+08 |
| **3** | 1.158E+08 |
| **4** | 1.225E+08 |
| **5** | 1.448E+08 |
| **6** | 1.404E+08 |
| **7** | 1.324E+08 |
| **8** | 1.527E+08 |
| **9** | 1.612E+08 |
| **10** | 1.540E+08 |
| **11** | 1.587E+08 |
| **12** | 1.617E+08 |
| **13** | 1.822E+08 |
| **14** | 1.986E+08 |
| **15** | 1.758E+08 |
| **16** | 1.936E+08 |
| **17** | 1.950E+08 |
| **18** | 2.052E+08 |
| **19** | 1.893E+08 |
| **20** | 2.100E+08 |
| **21** | 2.287E+08 |
| **22** | 2.201E+08 |
| **23** | 2.486E+08 |
| **24** | 2.422E+08 |
| **25** | 2.649E+08 |
| **26** | 2.906E+08 |
| **27** | 2.700E+08 |
| **28** | 3.019E+08 |
| **29** | 2.955E+08 |
| **30** | 3.130E+08 |
| **31** | 3.434E+08 |
| **32** | 3.305E+08 |
| **33** | 3.574E+08 |
| **34** | 3.755E+08 |
| **35** | 4.659E+08 |
| **36** | 4.553E+08 |
| **37** | 5.326E+08 |
| **38** | 5.219E+08 |
| **39** | 6.863E+08 |
| **40** | 7.583E+08 |
| **41** | 7.034E+08 |
| **42** | 8.343E+08 |
| **43** | 8.818E+08 |
| **44** | 1.055E+09 |
| **45** | 1.039E+09 |
| **46** | 1.231E+09 |
| **47** | 1.627E+09 |
| **48** | 1.767E+09 |
| **49** | 1.745E+09 |
| **50** | 2.097E+09 |
| **51** | 2.185E+09 |

**Table S14.** [$^{15}$N,$^{1}$H]-HSQC peak intensities of **a$_3$CuMT1** (N40) binding **Cu$^+$**.

| | Peak Intensity |
|---|---|
| **1** | 1.104E+10 |
| **2** | 9.318E+09 |
| **3** | 9.618E+09 |
| **4** | 9.708E+09 |
| **5** | 7.253E+09 |
| **6** | 6.720E+09 |
| **7** | 6.906E+09 |
| **8** | 6.325E+09 |
| **9** | 6.134E+09 |
| **10** | 4.640E+09 |
| **11** | 4.423E+09 |
| **12** | 3.958E+09 |
| **13** | 3.540E+09 |
| **14** | 3.580E+09 |
| **15** | 2.682E+09 |
| **16** | 2.747E+09 |
| **17** | 2.742E+09 |
| **18** | 2.477E+09 |
| **19** | 2.325E+09 |
| **20** | 1.945E+09 |
| **21** | 1.880E+09 |

**Table S15.** [$^{15}$N,$^{1}$H]-HSQC peak intensities of **a$_3$CuMT2** (N40 G43S) binding **Cu$^+$**.

| | Peak Intensity |
|---|---|
| **1** | 9.414E+09 |
| **2** | 1.037E+10 |
| **3** | 9.887E+09 |
| **4** | 9.506E+09 |
| **5** | 7.271E+09 |
| **6** | 7.260E+09 |
| **7** | 7.204E+09 |
| **8** | 6.736E+09 |
| **9** | 6.185E+09 |
| **10** | 5.361E+09 |
| **11** | 5.258E+09 |
| **12** | 4.781E+09 |
| **13** | 4.170E+09 |
| **14** | 3.699E+09 |
| **15** | 3.137E+09 |
| **16** | 3.200E+09 |
| **17** | 3.017E+09 |
| **18** | 2.642E+09 |
| **19** | 2.378E+09 |
| **20** | 1.692E+09 |
| **21** | 1.749E+09 |
| **22** | 1.579E+09 |
| **23** | 1.504E+09 |
| **24** | 1.618E+09 |
| **25** | 1.439E+09 |
| **26** | 1.363E+09 |
| **27** | 1.341E+09 |
| **28** | 1.285E+09 |
| **29** | 1.329E+09 |
| **30** | 1.226E+09 |

**Table S16.** $[^{15}N,^{1}H]$-HSQC peak intensities of $\mathbf{a_4}$**CdMT** (N28) binding $\mathbf{Cu^+}$.

| | Peak Intensity |
|---|---|
| **1** | 1.058E+09 |
| **2** | 1.171E+09 |
| **3** | 1.281E+09 |
| **4** | 1.264E+09 |
| **5** | 1.336E+09 |
| **6** | 1.218E+09 |
| **7** | 1.233E+09 |
| **8** | 1.457E+09 |
| **9** | 1.407E+09 |
| **10** | 1.331E+09 |
| **11** | 1.444E+09 |
| **12** | 1.464E+09 |
| **13** | 1.699E+09 |
| **14** | 1.484E+09 |
| **15** | 1.855E+09 |
| **16** | 1.700E+09 |
| **17** | 1.627E+09 |
| **18** | 1.962E+09 |
| **19** | 1.903E+09 |
| **20** | 2.121E+09 |
| **21** | 2.132E+09 |
| **22** | 2.098E+09 |
| **23** | 2.066E+09 |
| **24** | 2.226E+09 |
| **25** | 2.347E+09 |
| **26** | 2.661E+09 |
| **27** | 2.727E+09 |
| **28** | 3.144E+09 |
| **29** | 3.374E+09 |
| **30** | 3.041E+09 |
| **31** | 3.905E+09 |

**Table S17.** $[^{15}N,^{1}H]$-HSQC peak intensities of $\mathbf{a_4}$**CuMT** (N46) binding $\mathbf{Cu^+}$.

| | Peak Intensity |
|---|---|
| **1** | 7.754E+08 |
| **2** | 2.070E+09 |
| **3** | 7.767E+08 |
| **4** | 1.112E+09 |
| **5** | 1.243E+10 |
| **6** | 8.495E+08 |
| **7** | 1.066E+09 |
| **8** | 7.115E+08 |
| **9** | 1.875E+09 |
| **10** | 2.842E+09 |
| **11** | 9.518E+08 |
| **12** | 7.400E+09 |
| **13** | 1.663E+09 |
| **14** | 5.280E+09 |
| **15** | 3.823E+09 |
| **16** | 2.050E+09 |
| **17** | 8.524E+08 |
| **18** | 5.789E+09 |
| **19** | 9.742E+08 |
| **20** | 3.434E+09 |
| **21** | 1.271E+09 |
| **22** | 1.082E+09 |
| **23** | 2.668E+09 |
| **24** | 1.136E+09 |
| **25** | 7.640E+08 |
| **26** | 6.626E+08 |
| **27** | 2.385E+09 |
| **28** | 7.926E+08 |
| **29** | 6.680E+08 |
| **30** | 8.307E+08 |
| **31** | 7.864E+08 |
| **32** | 8.649E+08 |
| **33** | 7.153E+08 |

**Table S18.** $[^{15}N,^{1}H]$-HSQC peak intensities of $\mathbf{a_4}$**UnMT** (N41) binding $\mathbf{Cu^+}$.

| | Peak Intensity |
|---|---|
| **1** | 1.898E+08 |
| **2** | 2.032E+08 |
| **3** | 2.352E+08 |
| **4** | 2.397E+08 |
| **5** | 2.444E+08 |
| **6** | 2.609E+08 |
| **7** | 3.913E+08 |
| **8** | 4.047E+08 |
| **9** | 4.520E+08 |
| **10** | 5.103E+08 |
| **11** | 4.887E+08 |
| **12** | 5.396E+08 |
| **13** | 5.129E+08 |
| **14** | 4.885E+08 |
| **15** | 5.981E+08 |
| **16** | 5.975E+08 |
| **17** | 6.137E+08 |
| **18** | 6.839E+08 |
| **19** | 6.972E+08 |
| **20** | 6.661E+08 |
| **21** | 6.899E+08 |
| **22** | 7.311E+08 |
| **23** | 8.401E+08 |
| **24** | 7.314E+08 |
| **25** | 7.808E+08 |
| **26** | 8.407E+08 |
| **27** | 9.206E+08 |
| **28** | 8.273E+08 |
| **29** | 8.600E+08 |
| **30** | 1.000E+09 |
| **31** | 9.755E+08 |
| **32** | 1.038E+09 |
| **33** | 1.001E+09 |
| **34** | 1.062E+09 |
| **35** | 1.022E+09 |
| **36** | 1.186E+09 |
| **37** | 1.192E+09 |
| **38** | 1.055E+09 |
| **39** | 1.158E+09 |
| **40** | 1.056E+09 |
| **41** | 1.302E+09 |
| **42** | 1.327E+09 |
| **43** | 1.322E+09 |
| **44** | 1.288E+09 |
| **45** | 1.498E+09 |
| **46** | 1.419E+09 |
| **47** | 1.593E+09 |
| **48** | 1.737E+09 |
| **49** | 1.789E+09 |
| **50** | 1.855E+09 |
| **51** | 1.976E+09 |
| **52** | 1.720E+09 |
| **53** | 2.161E+09 |
| **54** | 2.100E+09 |
| **55** | 2.591E+09 |
| **56** | 2.973E+09 |
| **57** | 4.151E+09 |

**Table S19.** [$^{15}$N,$^{1}$H]-HSQC peak intensities of **HpCdMT** binding **Cu$^{+}$**.

| | Peak Intensity |
|---|---|
| **1** | 6.881E+08 |
| **2** | 7.509E+08 |
| **3** | 6.826E+08 |
| **4** | 6.917E+08 |
| **5** | 7.517E+08 |
| **6** | 7.675E+08 |
| **7** | 8.110E+08 |
| **8** | 8.131E+08 |
| **9** | 7.912E+08 |
| **10** | 8.127E+08 |
| **11** | 7.574E+08 |
| **12** | 8.250E+08 |
| **13** | 8.494E+08 |
| **14** | 8.593E+08 |
| **15** | 1.141E+09 |
| **16** | 1.210E+09 |
| **17** | 1.086E+09 |
| **18** | 1.143E+09 |
| **19** | 1.204E+09 |
| **20** | 1.375E+09 |
| **21** | 1.399E+09 |
| **22** | 1.404E+09 |
| **23** | 1.472E+09 |
| **24** | 1.643E+09 |
| **25** | 1.555E+09 |
| **26** | 1.704E+09 |
| **27** | 1.874E+09 |
| **28** | 2.189E+09 |
| **29** | 3.205E+09 |
| **30** | 3.691E+09 |
| **31** | 4.018E+09 |

**Table S20.** [$^{15}$N,$^{1}$H]-HSQC peak intensities of **HpCuMT** binding **Cu$^{+}$**.

| | Peak Intensity |
|---|---|
| **1** | 1.520E+08 |
| **2** | 2.271E+08 |
| **3** | 2.310E+08 |
| **4** | 2.257E+08 |
| **5** | 2.505E+08 |
| **6** | 2.710E+08 |
| **7** | 2.473E+08 |
| **8** | 2.432E+08 |
| **9** | 3.142E+08 |
| **10** | 3.417E+08 |
| **11** | 3.708E+08 |
| **12** | 3.541E+08 |
| **13** | 3.631E+08 |
| **14** | 3.994E+08 |
| **15** | 4.393E+08 |
| **16** | 4.309E+08 |
| **17** | 4.370E+08 |
| **18** | 5.117E+08 |
| **19** | 5.055E+08 |
| **20** | 5.238E+08 |
| **21** | 5.414E+08 |
| **22** | 5.160E+08 |
| **23** | 5.676E+08 |
| **24** | 5.602E+08 |
| **25** | 5.229E+08 |
| **26** | 5.774E+08 |
| **27** | 5.571E+08 |
| **28** | 5.549E+08 |
| **29** | 6.523E+08 |
| **30** | 6.452E+08 |
| **31** | 6.546E+08 |
| **32** | 6.242E+08 |
| **33** | 7.012E+08 |
| **34** | 7.150E+08 |
| **35** | 6.930E+08 |
| **36** | 7.927E+08 |
| **37** | 8.406E+08 |
| **38** | 8.295E+08 |
| **39** | 7.352E+08 |
| **40** | 8.704E+08 |
| **41** | 8.546E+08 |
| **42** | 9.612E+08 |
| **43** | 9.096E+08 |
| **44** | 9.480E+08 |
| **45** | 1.024E+09 |
| **46** | 1.061E+09 |
| **47** | 9.379E+08 |
| **48** | 1.018E+09 |
| **49** | 9.697E+08 |
| **50** | 1.217E+09 |
| **51** | 1.302E+09 |
| **52** | 1.754E+09 |

**Table S21.** [$^{15}$N,$^{1}$H]-HSQC peak intensities of **HpUnMT1** binding **Cu$^{+}$**.

| | Peak Intensity |
|---|---|
| **1** | 1.332E+10 |
| **2** | 1.336E+10 |
| **3** | 1.333E+10 |
| **4** | 1.231E+10 |
| **5** | 1.127E+10 |
| **6** | 1.172E+10 |
| **7** | 1.176E+10 |
| **8** | 1.136E+10 |
| **9** | 1.115E+10 |
| **10** | 1.086E+10 |
| **11** | 1.119E+10 |
| **12** | 1.093E+10 |
| **13** | 9.896E+09 |
| **14** | 9.567E+09 |
| **15** | 9.683E+09 |
| **16** | 8.688E+09 |
| **17** | 9.402E+09 |
| **18** | 8.850E+09 |
| **19** | 8.490E+09 |
| **20** | 8.261E+09 |
| **21** | 8.116E+09 |
| **22** | 7.992E+09 |
| **23** | 7.325E+09 |
| **24** | 7.778E+09 |
| **25** | 7.886E+09 |
| **26** | 7.664E+09 |
| **27** | 7.490E+09 |
| **28** | 7.746E+09 |
| **29** | 6.909E+09 |
| **30** | 6.500E+09 |
| **31** | 6.233E+09 |
| **32** | 6.909E+09 |
| **33** | 6.149E+09 |
| **34** | 5.744E+09 |
| **35** | 5.511E+09 |
| **36** | 5.380E+09 |
| **37** | 5.157E+09 |
| **38** | 4.889E+09 |
| **39** | 4.814E+09 |
| **40** | 4.631E+09 |
| **41** | 3.964E+09 |
| **42** | 3.518E+09 |
| **43** | 3.642E+09 |
| **44** | 3.362E+09 |
| **45** | 3.408E+09 |
| **46** | 3.293E+09 |
| **47** | 3.044E+09 |
| **48** | 2.963E+09 |
| **49** | 3.107E+09 |
| **50** | 2.758E+09 |
| **51** | 2.210E+09 |
| **52** | 1.484E+09 |

**Table S22.** [$^{15}$N,$^{1}$H]-HSQC peak intensities of **HpUnMT2** binding **Cu$^{+}$**.

| | Peak Intensity |
|---|---|
| **1** | 1.917E+10 |
| **2** | 1.912E+10 |
| **3** | 1.499E+10 |
| **4** | 1.732E+10 |
| **5** | 1.491E+10 |
| **6** | 1.639E+10 |
| **7** | 1.602E+10 |
| **8** | 1.653E+10 |
| **9** | 1.634E+10 |
| **10** | 1.540E+10 |
| **11** | 1.487E+10 |
| **12** | 1.588E+10 |
| **13** | 1.451E+10 |
| **14** | 1.423E+10 |
| **15** | 1.494E+10 |
| **16** | 1.288E+10 |
| **17** | 1.416E+10 |
| **18** | 1.272E+10 |
| **19** | 1.285E+10 |
| **20** | 1.201E+10 |
| **21** | 1.212E+10 |
| **22** | 1.115E+10 |
| **23** | 1.181E+10 |
| **24** | 1.131E+10 |
| **25** | 1.098E+10 |
| **26** | 1.060E+10 |
| **27** | 1.146E+10 |
| **28** | 1.087E+10 |
| **29** | 1.056E+10 |
| **30** | 1.023E+10 |
| **31** | 9.965E+09 |
| **32** | 9.673E+09 |
| **33** | 8.258E+09 |
| **34** | 7.659E+09 |
| **35** | 8.120E+09 |
| **36** | 7.021E+09 |
| **37** | 6.814E+09 |
| **38** | 6.372E+09 |
| **39** | 6.213E+09 |
| **40** | 6.309E+09 |
| **41** | 5.747E+09 |
| **42** | 5.598E+09 |
| **43** | 5.566E+09 |
| **44** | 5.447E+09 |
| **45** | 5.387E+09 |
| **46** | 5.221E+09 |
| **47** | 4.292E+09 |
| **48** | 4.161E+09 |
| **49** | 3.868E+09 |
| **50** | 3.087E+09 |
| **51** | 1.691E+09 |
| **52** | 1.352E+09 |
| **53** | 1.284E+09 |
| **54** | 1.134E+09 |

**Table S23.** $T_2$ relaxation times of **a$_1$CdMT** (N20) binding **$Cd^{2+}$**. The standard error for $T_2$ relaxation times was estimated using a Monte Carlo approach ($T_2$ MC SE) that combined the standard error for the $T_2$ fit ($T_2$ SE) and the standard error for fitting the initial intensity (M0 SE). The M0 SE is given for an intensity of one and is therefore a relative measure. The fitted absolute initial (delay = 0 ms) intensity (M0) is given in a separate column. The degrees of freedom (df) indicate the number of recorded data points (9 data points for a df of 7).

| | $T_2$ Relaxation [ms] | $T_2$ MC SE [ms] | $T_2$ SE [ms] | M0 SE [rel] | M0 [abs] | df |
|---|---|---|---|---|---|---|
| **1** | 180.89 | 4.83 | 4.73 | 0.014 | 1.393E+10 | 7 |
| **2** | 186.58 | 5.57 | 5.67 | 0.016 | 1.616E+10 | 7 |
| **3** | 170.49 | 3.92 | 3.92 | 0.013 | 9.138E+09 | 7 |
| **4** | 176.15 | 3.82 | 3.84 | 0.012 | 1.601E+10 | 7 |
| **5** | 205.74 | 6.72 | 6.71 | 0.017 | 5.044E+09 | 7 |
| **6** | 169.63 | 3.52 | 3.60 | 0.012 | 1.073E+10 | 7 |
| **7** | 174.47 | 4.91 | 5.04 | 0.016 | 1.049E+10 | 7 |
| **8** | 183.19 | 3.73 | 3.73 | 0.011 | 1.348E+10 | 7 |
| **9** | 184.25 | 11.08 | 10.85 | 0.032 | 1.140E+10 | 7 |
| **10** | 160.90 | 5.65 | 5.57 | 0.020 | 4.208E+09 | 7 |
| **11** | 148.75 | 2.46 | 2.50 | 0.010 | 1.243E+10 | 7 |
| **12** | 200.29 | 5.78 | 5.73 | 0.015 | 1.288E+10 | 7 |
| **13** | 169.81 | 6.36 | 6.46 | 0.021 | 7.525E+09 | 7 |
| **14** | 188.18 | 5.94 | 6.33 | 0.018 | 1.366E+10 | 7 |
| **15** | 184.36 | 8.97 | 9.34 | 0.027 | 8.260E+09 | 7 |
| **16** | 171.63 | 4.29 | 4.15 | 0.014 | 1.232E+10 | 7 |
| **17** | 187.54 | 5.06 | 5.28 | 0.015 | 1.480E+10 | 7 |
| **18** | 181.40 | 4.02 | 4.25 | 0.013 | 1.273E+10 | 7 |
| **19** | 144.09 | 4.39 | 4.39 | 0.019 | 3.385E+09 | 7 |
| **20** | 183.59 | 6.40 | 6.54 | 0.019 | 1.013E+10 | 7 |
| **21** | 192.02 | 4.98 | 4.94 | 0.014 | 1.290E+10 | 7 |
| **22** | 166.94 | 5.24 | 5.35 | 0.018 | 1.200E+10 | 7 |
| **23** | 174.37 | 6.07 | 6.10 | 0.019 | 9.025E+09 | 7 |
| **24** | 195.42 | 5.46 | 5.59 | 0.015 | 5.494E+09 | 7 |
| **25** | 178.14 | 4.62 | 4.42 | 0.014 | 1.466E+10 | 7 |
| **26** | 193.41 | 4.80 | 4.81 | 0.013 | 1.892E+10 | 7 |
| **27** | 136.03 | 6.14 | 6.12 | 0.028 | 1.868E+09 | 7 |
| **28** | 153.77 | 3.75 | 3.73 | 0.014 | 1.161E+10 | 7 |
| **29** | 201.25 | 5.00 | 4.98 | 0.013 | 1.412E+10 | 7 |
| **30** | 145.52 | 4.04 | 3.98 | 0.017 | 9.985E+09 | 7 |
| **31** | 188.69 | 4.53 | 4.41 | 0.013 | 1.367E+10 | 7 |
| **32** | 179.38 | 5.43 | 5.51 | 0.017 | 9.787E+09 | 7 |
| **33** | 154.48 | 4.34 | 4.48 | 0.017 | 1.225E+10 | 7 |
| **34** | 189.61 | 4.54 | 4.41 | 0.012 | 1.299E+10 | 7 |
| **35** | 168.82 | 4.50 | 4.61 | 0.015 | 1.366E+10 | 7 |
| **36** | 143.03 | 4.72 | 4.57 | 0.019 | 4.096E+09 | 7 |
| **37** | 195.58 | 4.47 | 4.60 | 0.012 | 1.126E+10 | 7 |
| **38** | 186.17 | 2.81 | 2.77 | 0.008 | 1.132E+10 | 7 |
| **39** | 188.08 | 4.49 | 4.35 | 0.012 | 1.243E+10 | 7 |
| **40** | 136.46 | 4.81 | 4.91 | 0.022 | 6.882E+09 | 7 |
| **41** | 171.59 | 4.34 | 4.38 | 0.014 | 1.343E+10 | 7 |
| **42** | 174.70 | 3.07 | 3.12 | 0.010 | 1.471E+10 | 7 |
| **43** | 150.98 | 7.27 | 7.10 | 0.028 | 1.298E+10 | 7 |
| **44** | 182.71 | 4.48 | 4.56 | 0.014 | 1.279E+10 | 7 |
| **45** | 169.98 | 4.60 | 4.71 | 0.016 | 1.244E+10 | 7 |
| **46** | 204.71 | 8.72 | 8.75 | 0.022 | 1.147E+10 | 7 |
| **47** | 166.75 | 4.03 | 3.99 | 0.014 | 7.597E+09 | 7 |
| **48** | 185.60 | 3.97 | 3.98 | 0.012 | 1.198E+10 | 7 |
| **49** | 194.24 | 3.91 | 3.88 | 0.011 | 1.338E+10 | 7 |
| **50** | 243.20 | 4.77 | 4.72 | 0.009 | 1.620E+10 | 7 |
| **51** | 142.68 | 3.91 | 3.86 | 0.017 | 1.426E+10 | 7 |
| **52** | 192.85 | 3.16 | 3.20 | 0.009 | 1.359E+10 | 7 |
| **53** | 202.19 | 3.93 | 4.04 | 0.010 | 1.277E+10 | 7 |
| **54** | 198.21 | 5.00 | 5.16 | 0.014 | 1.217E+10 | 7 |
| **55** | 161.71 | 3.92 | 3.85 | 0.014 | 1.093E+10 | 7 |
| **56** | 190.50 | 4.54 | 4.69 | 0.013 | 1.240E+10 | 7 |
| **57** | 164.29 | 4.78 | 4.76 | 0.017 | 1.085E+10 | 7 |
| **58** | 246.68 | 6.43 | 6.26 | 0.012 | 1.494E+10 | 7 |
| **59** | 198.29 | 3.62 | 3.68 | 0.010 | 1.483E+10 | 7 |
| **60** | 194.00 | 4.57 | 4.59 | 0.012 | 1.432E+10 | 7 |

**Table S24.** $T_2$ relaxation times of **a₂CuMT** (N36) binding $\mathbf{Cd^{2+}}$. The standard error for $T_2$ relaxation times was estimated using a Monte Carlo approach ($T_2$ MC SE) that combined the standard error for the $T_2$ fit ($T_2$ SE) and the standard error for fitting the initial intensity (M0 SE). The M0 SE is given for an intensity of one and is therefore a relative measure. The fitted absolute initial (delay = 0 ms) intensity (M0) is given in a separate column. The degrees of freedom (df) indicate the number of recorded data points (9 data points for a df of 7).

| | $T_2$ Relaxation [ms] | $T_2$ MC SE [ms] | $T_2$ SE [ms] | M0 SE [rel] | M0 [abs] | df |
|---|---|---|---|---|---|---|
| **1** | 142.96 | 3.54 | 3.57 | 0.015 | 3.232E+10 | 7 |
| **2** | 167.82 | 2.99 | 2.97 | 0.010 | 3.066E+10 | 7 |
| **3** | 129.97 | 3.84 | 3.81 | 0.019 | 3.027E+10 | 7 |
| **4** | 128.64 | 5.35 | 5.38 | 0.027 | 2.890E+10 | 7 |
| **5** | 107.62 | 3.53 | 3.40 | 0.022 | 1.959E+10 | 7 |
| **6** | 122.59 | 3.47 | 3.40 | 0.018 | 3.047E+10 | 7 |
| **7** | 164.18 | 3.42 | 3.44 | 0.012 | 3.105E+10 | 7 |
| **8** | 166.97 | 4.28 | 4.34 | 0.015 | 2.324E+10 | 7 |
| **9** | 121.35 | 3.71 | 3.71 | 0.020 | 2.843E+10 | 7 |
| **10** | 143.64 | 4.54 | 4.40 | 0.019 | 2.404E+10 | 7 |
| **11** | 195.66 | 4.90 | 4.90 | 0.013 | 3.361E+10 | 7 |
| **12** | 136.71 | 4.91 | 4.89 | 0.022 | 2.856E+10 | 7 |
| **13** | 190.15 | 5.18 | 5.21 | 0.015 | 3.337E+10 | 7 |
| **14** | 163.95 | 4.00 | 3.93 | 0.014 | 8.667E+09 | 7 |
| **15** | 126.83 | 3.88 | 3.80 | 0.019 | 1.659E+10 | 7 |
| **16** | 196.53 | 6.81 | 6.65 | 0.018 | 3.427E+10 | 7 |
| **17** | 154.74 | 6.49 | 6.52 | 0.025 | 3.111E+10 | 7 |
| **18** | 160.58 | 4.64 | 4.67 | 0.017 | 2.132E+10 | 7 |
| **19** | 214.87 | 4.17 | 4.10 | 0.009 | 3.557E+10 | 7 |
| **20** | 146.57 | 6.59 | 6.59 | 0.027 | 2.749E+10 | 7 |
| **21** | 155.75 | 3.18 | 3.22 | 0.012 | 2.918E+10 | 7 |
| **22** | 66.77 | 3.75 | 3.84 | 0.045 | 1.479E+10 | 7 |
| **23** | 159.22 | 5.38 | 5.51 | 0.020 | 3.556E+10 | 7 |
| **24** | 157.57 | 4.69 | 4.42 | 0.016 | 2.723E+10 | 7 |
| **25** | 131.11 | 3.32 | 3.35 | 0.016 | 2.620E+10 | 7 |
| **26** | 159.24 | 4.38 | 4.32 | 0.016 | 2.954E+10 | 7 |
| **27** | 179.37 | 4.28 | 4.22 | 0.013 | 3.781E+10 | 7 |
| **28** | 185.35 | 4.16 | 4.29 | 0.012 | 3.880E+10 | 7 |
| **29** | 178.77 | 3.76 | 3.83 | 0.012 | 3.392E+10 | 7 |
| **30** | 126.11 | 4.32 | 4.39 | 0.022 | 2.701E+10 | 7 |
| **31** | 140.59 | 3.50 | 3.48 | 0.015 | 4.191E+10 | 7 |
| **32** | 204.36 | 5.84 | 5.81 | 0.015 | 2.958E+10 | 7 |
| **33** | 148.27 | 4.20 | 4.17 | 0.017 | 2.655E+10 | 7 |
| **34** | 133.24 | 3.40 | 3.42 | 0.016 | 1.851E+10 | 7 |
| **35** | 170.45 | 4.24 | 4.07 | 0.013 | 3.826E+10 | 7 |
| **36** | 103.20 | 3.68 | 3.66 | 0.024 | 2.021E+10 | 7 |
| **37** | 170.24 | 4.09 | 4.02 | 0.013 | 2.464E+10 | 7 |
| **38** | 250.72 | 5.02 | 4.89 | 0.009 | 4.837E+10 | 7 |
| **39** | 183.61 | 3.87 | 3.85 | 0.011 | 4.107E+10 | 7 |
| **40** | 177.78 | 4.34 | 4.31 | 0.013 | 3.864E+10 | 7 |
| **41** | 157.71 | 3.87 | 3.82 | 0.014 | 3.022E+10 | 7 |
| **42** | 195.96 | 7.99 | 8.04 | 0.022 | 4.040E+10 | 7 |
| **43** | 177.51 | 3.98 | 3.99 | 0.012 | 3.611E+10 | 7 |
| **44** | 166.62 | 3.86 | 4.01 | 0.014 | 4.480E+10 | 7 |
| **45** | 142.90 | 4.21 | 4.16 | 0.018 | 2.411E+10 | 7 |
| **46** | 153.27 | 4.42 | 4.48 | 0.017 | 3.103E+10 | 7 |
| **47** | 110.44 | 4.88 | 4.85 | 0.030 | 2.673E+10 | 7 |
| **48** | 152.71 | 3.43 | 3.49 | 0.014 | 2.881E+10 | 7 |
| **49** | 195.11 | 3.87 | 3.86 | 0.010 | 4.221E+10 | 7 |
| **50** | 135.08 | 3.73 | 3.65 | 0.017 | 3.612E+10 | 7 |
| **51** | 150.82 | 5.02 | 4.81 | 0.019 | 3.575E+10 | 7 |
| **52** | 172.52 | 4.54 | 4.45 | 0.014 | 3.297E+10 | 7 |
| **53** | 144.44 | 4.59 | 4.49 | 0.019 | 3.101E+10 | 7 |
| **54** | 185.81 | 4.01 | 4.12 | 0.012 | 4.520E+10 | 7 |
| **55** | 176.78 | 4.49 | 4.38 | 0.014 | 3.638E+10 | 7 |
| **56** | 85.27 | 3.03 | 3.14 | 0.027 | 2.185E+10 | 7 |
| **57** | 114.46 | 3.04 | 2.97 | 0.017 | 2.058E+10 | 7 |
| **58** | 185.66 | 3.66 | 3.64 | 0.011 | 3.742E+10 | 7 |
| **59** | 161.71 | 4.24 | 4.21 | 0.015 | 3.080E+10 | 7 |
| **60** | 171.34 | 5.00 | 5.08 | 0.017 | 4.057E+10 | 7 |

**Table S25.** $T_2$ relaxation times of **a$_3$CuMT1** (N40) binding **$Cd^{2+}$**. The standard error for $T_2$ relaxation times was estimated using a Monte Carlo approach ($T_2$ MC SE) that combined the standard error for the $T_2$ fit ($T_2$ SE) and the standard error for fitting the initial intensity (M0 SE). The M0 SE is given for an intensity of one and is therefore a relative measure. The fitted absolute initial (delay = 0 ms) intensity (M0) is given in a separate column. The degrees of freedom (df) indicate the number of recorded data points (9 data points for a df of 7).

| | $T_2$ Relaxation [ms] | $T_2$ MC SE [ms] | $T_2$ SE [ms] | M0 SE [rel] | M0 [abs] | df |
|---|---|---|---|---|---|---|
| **1** | 88.81 | 1.75 | 1.81 | 0.015 | 1.026E+10 | 7 |
| **2** | 67.57 | 1.74 | 1.74 | 0.020 | 1.541E+10 | 7 |
| **3** | 91.24 | 2.12 | 2.11 | 0.017 | 3.120E+09 | 7 |
| **4** | 147.46 | 4.94 | 4.95 | 0.020 | 6.084E+09 | 7 |
| **5** | 77.00 | 3.44 | 3.47 | 0.034 | 7.594E+09 | 7 |
| **6** | 49.36 | 1.40 | 1.42 | 0.024 | 1.230E+10 | 7 |
| **7** | 31.14 | 0.83 | 0.82 | 0.025 | 6.824E+09 | 7 |
| **8** | 95.94 | 2.87 | 2.89 | 0.021 | 2.320E+10 | 7 |
| **9** | 63.75 | 1.73 | 1.73 | 0.022 | 1.285E+10 | 7 |
| **10** | 89.19 | 2.75 | 2.73 | 0.022 | 7.488E+09 | 7 |
| **11** | 42.45 | 1.84 | 1.81 | 0.037 | 5.600E+09 | 7 |
| **12** | 179.07 | 4.55 | 4.49 | 0.014 | 4.657E+10 | 7 |
| **13** | 104.81 | 3.82 | 3.99 | 0.026 | 5.291E+10 | 7 |
| **14** | 176.66 | 4.85 | 4.93 | 0.015 | 4.345E+10 | 7 |
| **15** | 140.30 | 4.28 | 4.32 | 0.019 | 4.336E+10 | 7 |
| **16** | 98.59 | 2.52 | 2.56 | 0.018 | 1.451E+10 | 7 |
| **17** | 38.56 | 1.06 | 1.05 | 0.024 | 1.576E+10 | 7 |
| **18** | 120.81 | 4.47 | 4.50 | 0.024 | 1.034E+10 | 7 |
| **19** | 145.13 | 7.07 | 6.92 | 0.029 | 5.213E+10 | 7 |
| **20** | 72.37 | 1.78 | 1.79 | 0.019 | 1.060E+10 | 7 |
| **21** | 147.30 | 4.43 | 4.51 | 0.018 | 4.176E+10 | 7 |
| **22** | 90.68 | 2.20 | 2.21 | 0.018 | 1.774E+10 | 7 |
| **23** | 115.80 | 2.68 | 2.71 | 0.016 | 5.740E+10 | 7 |
| **24** | 120.58 | 4.04 | 4.07 | 0.022 | 3.900E+09 | 7 |
| **25** | 45.31 | 1.26 | 1.29 | 0.024 | 1.761E+10 | 7 |
| **26** | 98.90 | 3.36 | 3.40 | 0.024 | 1.305E+10 | 7 |
| **27** | 75.04 | 2.42 | 2.35 | 0.024 | 9.002E+09 | 7 |
| **28** | 173.14 | 5.52 | 5.47 | 0.018 | 5.316E+10 | 7 |
| **29** | 134.99 | 3.67 | 3.60 | 0.017 | 4.249E+10 | 7 |
| **30** | 113.36 | 2.83 | 2.86 | 0.017 | 9.164E+09 | 7 |
| **31** | 135.12 | 3.83 | 3.86 | 0.018 | 4.460E+10 | 7 |
| **32** | 132.35 | 5.65 | 5.79 | 0.028 | 4.648E+10 | 7 |
| **33** | 129.62 | 5.74 | 5.70 | 0.028 | 7.123E+09 | 7 |
| **34** | 79.87 | 3.42 | 3.52 | 0.033 | 6.102E+09 | 7 |
| **35** | 156.22 | 5.95 | 6.00 | 0.023 | 6.301E+10 | 7 |
| **36** | 136.90 | 3.29 | 3.28 | 0.015 | 4.625E+10 | 7 |
| **37** | 75.65 | 1.76 | 1.67 | 0.017 | 1.631E+10 | 7 |
| **38** | 89.39 | 2.52 | 2.51 | 0.020 | 1.355E+10 | 7 |
| **39** | 73.07 | 1.58 | 1.63 | 0.017 | 1.760E+10 | 7 |
| **40** | 100.21 | 4.30 | 4.00 | 0.028 | 1.644E+10 | 7 |
| **41** | 137.32 | 3.24 | 3.19 | 0.014 | 2.218E+10 | 7 |
| **42** | 55.13 | 1.89 | 1.85 | 0.028 | 1.011E+10 | 7 |
| **43** | 107.89 | 2.89 | 2.85 | 0.018 | 2.340E+10 | 7 |
| **44** | 129.62 | 2.02 | 2.06 | 0.010 | 1.628E+10 | 7 |
| **45** | 158.23 | 5.84 | 5.70 | 0.021 | 3.290E+10 | 7 |
| **46** | 142.88 | 4.55 | 4.50 | 0.019 | 7.928E+10 | 7 |
| **47** | 66.65 | 1.61 | 1.54 | 0.018 | 9.397E+09 | 7 |
| **48** | 64.11 | 1.85 | 1.80 | 0.022 | 1.097E+10 | 7 |
| **49** | 148.45 | 3.38 | 3.40 | 0.014 | 3.346E+10 | 7 |
| **50** | 193.17 | 7.10 | 6.93 | 0.019 | 1.274E+10 | 7 |
| **51** | 55.18 | 1.63 | 1.62 | 0.024 | 9.996E+09 | 7 |
| **52** | 84.78 | 1.75 | 1.75 | 0.015 | 2.194E+10 | 7 |
| **53** | 116.89 | 4.54 | 4.39 | 0.025 | 2.963E+10 | 7 |
| **54** | 60.19 | 1.68 | 1.64 | 0.022 | 3.774E+10 | 7 |
| **55** | 142.91 | 4.56 | 4.44 | 0.019 | 1.677E+10 | 7 |
| **56** | 95.13 | 3.47 | 3.25 | 0.024 | 7.819E+09 | 7 |
| **57** | 213.69 | 8.74 | 8.74 | 0.020 | 1.001E+10 | 7 |
| **58** | 142.15 | 3.64 | 3.65 | 0.016 | 4.515E+10 | 7 |
| **59** | 98.07 | 2.26 | 2.32 | 0.017 | 8.091E+09 | 7 |
| **60** | 101.09 | 2.38 | 2.31 | 0.016 | 3.027E+10 | 7 |
| **61** | 59.14 | 1.70 | 1.65 | 0.022 | 2.118E+10 | 7 |

**Table S26.** $T_2$ relaxation times of **a$_3$CuMT2** (N40 G43S) binding **$Cd^{2+}$**. The standard error for $T_2$ relaxation times was estimated using a Monte Carlo approach ($T_2$ MC SE) that combined the standard error for the $T_2$ fit ($T_2$ SE) and the standard error for fitting the initial intensity (M0 SE). The M0 SE is given for an intensity of one and is therefore a relative measure. The fitted absolute initial (delay = 0 ms) intensity (M0) is given in a separate column. The degrees of freedom (df) indicate the number of recorded data points (9 data points for a df of 7).

| | $T_2$ Relaxation [ms] | $T_2$ MC SE [ms] | $T_2$ SE [ms] | M0 SE [rel] | M0 [abs] | df |
|---|---|---|---|---|---|---|
| **1** | 114.39 | 4.35 | 4.41 | 0.026 | 2.149E+10 | 7 |
| **2** | 42.15 | 2.08 | 2.12 | 0.044 | 4.970E+09 | 7 |
| **3** | 141.00 | 3.85 | 3.82 | 0.017 | 1.219E+10 | 7 |
| **4** | 185.86 | 6.13 | 6.16 | 0.018 | 9.789E+09 | 7 |
| **5** | 148.27 | 3.20 | 3.39 | 0.014 | 7.352E+10 | 7 |
| **6** | 81.63 | 3.15 | 3.17 | 0.029 | 5.403E+09 | 7 |
| **7** | 27.14 | 2.70 | 2.58 | 0.093 | 1.328E+09 | 7 |
| **8** | 55.01 | 3.55 | 3.52 | 0.052 | 2.157E+09 | 7 |
| **9** | 116.95 | 3.18 | 3.17 | 0.018 | 8.202E+09 | 7 |
| **10** | 102.09 | 6.74 | 6.67 | 0.045 | 1.585E+09 | 7 |
| **11** | 106.30 | 4.65 | 4.83 | 0.031 | 3.914E+10 | 7 |
| **12** | 131.38 | 3.26 | 3.22 | 0.015 | 2.003E+10 | 7 |
| **13** | 161.07 | 3.40 | 3.34 | 0.012 | 4.673E+10 | 7 |
| **14** | 138.86 | 4.37 | 4.16 | 0.019 | 3.249E+10 | 7 |
| **15** | 28.77 | 5.11 | 4.38 | 0.147 | 1.817E+09 | 7 |
| **16** | 52.27 | 4.18 | 4.15 | 0.066 | 1.251E+09 | 7 |
| **17** | 146.31 | 3.57 | 3.52 | 0.015 | 3.344E+10 | 7 |
| **18** | 133.59 | 5.01 | 4.99 | 0.023 | 3.478E+10 | 7 |
| **19** | 113.75 | 2.95 | 2.91 | 0.017 | 5.240E+09 | 7 |
| **20** | 169.01 | 4.19 | 4.17 | 0.014 | 3.245E+10 | 7 |
| **21** | 37.09 | 7.09 | 6.65 | 0.160 | 9.679E+08 | 7 |
| **22** | 115.47 | 2.85 | 2.81 | 0.016 | 4.486E+10 | 7 |
| **23** | 26.28 | 3.42 | 3.24 | 0.123 | 2.081E+09 | 7 |
| **24** | 180.23 | 3.98 | 3.96 | 0.012 | 3.686E+10 | 7 |
| **25** | 39.10 | 1.39 | 1.41 | 0.032 | 4.088E+09 | 7 |
| **26** | 27.63 | 8.09 | 6.58 | 0.233 | 8.074E+08 | 7 |
| **27** | 132.57 | 3.29 | 3.22 | 0.015 | 3.692E+10 | 7 |
| **28** | 100.61 | 2.92 | 2.81 | 0.019 | 1.420E+10 | 7 |
| **29** | 48.13 | 1.55 | 1.51 | 0.026 | 2.386E+09 | 7 |
| **30** | 98.83 | 2.81 | 2.81 | 0.020 | 2.063E+10 | 7 |
| **31** | 96.30 | 2.55 | 2.60 | 0.019 | 1.507E+10 | 7 |
| **32** | 75.91 | 2.89 | 2.93 | 0.029 | 2.203E+09 | 7 |
| **33** | 135.52 | 3.16 | 3.18 | 0.015 | 1.470E+10 | 7 |
| **34** | 17.35 | 2.47 | 2.31 | 0.167 | 2.318E+09 | 7 |
| **35** | 74.98 | 4.05 | 3.92 | 0.040 | 3.184E+09 | 7 |
| **36** | 145.77 | 5.49 | 5.30 | 0.022 | 3.911E+10 | 7 |
| **37** | 175.19 | 3.97 | 4.05 | 0.013 | 3.962E+10 | 7 |
| **38** | 162.94 | 3.25 | 3.19 | 0.011 | 1.735E+10 | 7 |
| **39** | 112.68 | 4.83 | 4.94 | 0.029 | 6.367E+09 | 7 |
| **40** | 71.55 | 10.43 | 9.49 | 0.102 | 1.424E+09 | 7 |
| **41** | 133.36 | 5.75 | 5.68 | 0.027 | 3.939E+09 | 7 |
| **42** | 69.47 | 3.04 | 3.00 | 0.033 | 7.664E+09 | 7 |
| **43** | 74.58 | 2.87 | 2.80 | 0.029 | 4.282E+09 | 7 |
| **44** | 23.55 | 2.04 | 1.90 | 0.085 | 3.313E+09 | 7 |
| **45** | 42.72 | 2.93 | 2.92 | 0.059 | 1.739E+09 | 7 |
| **46** | 100.18 | 5.41 | 5.36 | 0.037 | 4.137E+09 | 7 |
| **47** | 58.84 | 5.52 | 5.16 | 0.071 | 2.096E+09 | 7 |
| **48** | 189.31 | 7.61 | 7.57 | 0.021 | 8.127E+09 | 7 |
| **49** | 134.58 | 3.11 | 3.04 | 0.014 | 3.102E+10 | 7 |
| **50** | 56.00 | 2.00 | 1.97 | 0.029 | 2.602E+10 | 7 |
| **51** | 136.14 | 3.51 | 3.51 | 0.016 | 3.309E+10 | 7 |
| **52** | 134.54 | 15.41 | 14.75 | 0.069 | 8.940E+08 | 7 |
| **53** | 159.20 | 6.35 | 6.27 | 0.023 | 4.734E+09 | 7 |
| **54** | 136.88 | 3.74 | 3.67 | 0.017 | 3.474E+10 | 7 |
| **55** | 36.06 | 2.33 | 2.23 | 0.056 | 1.156E+09 | 7 |
| **56** | 91.77 | 3.06 | 3.05 | 0.024 | 3.752E+09 | 7 |

**Table S27.** $T_2$ relaxation times of **a$_4$CdMT** (N28) binding **$Cd^{2+}$**. The standard error for $T_2$ relaxation times was estimated using a Monte Carlo approach ($T_2$ MC SE) that combined the standard error for the $T_2$ fit ($T_2$ SE) and the standard error for fitting the initial intensity (M0 SE). The M0 SE is given for an intensity of one and is therefore a relative measure. The fitted absolute initial (delay = 0 ms) intensity (M0) is given in a separate column. The degrees of freedom (df) indicate the number of recorded data points (9 data points for a df of 7).

| | $T_2$ Relaxation [ms] | $T_2$ MC SE [ms] | $T_2$ SE [ms] | M0 SE [rel] | M0 [abs] | df |
|---|---|---|---|---|---|---|
| **1** | 180.75 | 5.50 | 5.52 | 0.017 | 4.125E+10 | 7 |
| **2** | 142.35 | 6.35 | 6.02 | 0.026 | 4.568E+10 | 7 |
| **3** | 155.08 | 5.04 | 4.90 | 0.019 | 4.315E+10 | 7 |
| **4** | 175.59 | 4.89 | 5.07 | 0.016 | 3.637E+10 | 7 |
| **5** | 137.72 | 5.21 | 5.18 | 0.023 | 2.184E+10 | 7 |
| **6** | 143.32 | 6.11 | 6.01 | 0.026 | 2.976E+10 | 7 |
| **7** | 150.15 | 5.92 | 5.65 | 0.022 | 2.602E+10 | 7 |
| **8** | 154.47 | 3.91 | 4.07 | 0.016 | 3.069E+10 | 7 |
| **9** | 136.60 | 4.11 | 4.12 | 0.019 | 3.536E+10 | 7 |
| **10** | 206.81 | 5.74 | 5.87 | 0.014 | 5.048E+10 | 7 |
| **11** | 126.05 | 5.18 | 5.04 | 0.026 | 4.614E+10 | 7 |
| **12** | 136.00 | 5.47 | 5.51 | 0.025 | 4.502E+10 | 7 |
| **13** | 124.41 | 3.63 | 3.53 | 0.018 | 1.934E+10 | 7 |
| **14** | 130.63 | 6.27 | 6.17 | 0.030 | 3.469E+10 | 7 |
| **15** | 174.98 | 3.71 | 3.64 | 0.012 | 4.612E+10 | 7 |
| **16** | 161.26 | 7.82 | 7.52 | 0.027 | 4.710E+10 | 7 |
| **17** | 149.79 | 6.04 | 6.02 | 0.024 | 4.754E+10 | 7 |
| **18** | 145.54 | 3.90 | 4.00 | 0.017 | 2.998E+10 | 7 |
| **19** | 165.88 | 4.94 | 4.95 | 0.017 | 4.605E+10 | 7 |
| **20** | 149.97 | 6.04 | 5.71 | 0.023 | 8.433E+09 | 7 |
| **21** | 167.32 | 8.92 | 8.42 | 0.029 | 4.289E+10 | 7 |
| **22** | 177.14 | 5.10 | 4.96 | 0.015 | 4.237E+10 | 7 |
| **23** | 163.06 | 5.56 | 5.55 | 0.020 | 4.263E+10 | 7 |
| **24** | 159.14 | 4.17 | 4.04 | 0.015 | 4.024E+10 | 7 |
| **25** | 172.43 | 5.09 | 5.01 | 0.016 | 3.552E+10 | 7 |
| **26** | 133.18 | 5.82 | 5.48 | 0.026 | 2.151E+10 | 7 |
| **27** | 140.90 | 4.47 | 4.43 | 0.019 | 3.627E+10 | 7 |
| **28** | 170.24 | 6.95 | 6.85 | 0.023 | 4.574E+10 | 7 |
| **29** | 169.29 | 5.43 | 5.34 | 0.018 | 4.319E+10 | 7 |
| **30** | 186.69 | 5.16 | 5.09 | 0.015 | 3.578E+10 | 7 |
| **31** | 171.77 | 5.11 | 5.11 | 0.017 | 4.873E+10 | 7 |
| **32** | 121.58 | 4.71 | 4.79 | 0.026 | 3.393E+10 | 7 |
| **33** | 199.23 | 9.73 | 9.54 | 0.025 | 9.028E+09 | 7 |
| **34** | 121.08 | 4.17 | 4.20 | 0.023 | 3.818E+10 | 7 |
| **35** | 167.46 | 4.74 | 4.85 | 0.016 | 3.712E+10 | 7 |
| **36** | 162.37 | 6.66 | 6.84 | 0.024 | 2.206E+10 | 7 |
| **37** | 135.74 | 5.40 | 5.36 | 0.025 | 3.862E+10 | 7 |
| **38** | 162.80 | 4.57 | 4.55 | 0.016 | 4.907E+10 | 7 |
| **39** | 161.49 | 6.65 | 6.46 | 0.023 | 4.699E+10 | 7 |
| **40** | 144.44 | 4.28 | 4.23 | 0.018 | 4.691E+10 | 7 |
| **41** | 140.14 | 4.09 | 4.00 | 0.018 | 3.599E+10 | 7 |
| **42** | 163.46 | 5.11 | 5.06 | 0.018 | 4.361E+10 | 7 |
| **43** | 208.42 | 10.40 | 10.33 | 0.025 | 5.060E+10 | 7 |
| **44** | 144.14 | 6.31 | 6.10 | 0.026 | 4.065E+10 | 7 |
| **45** | 117.40 | 5.33 | 5.18 | 0.029 | 3.275E+10 | 7 |
| **46** | 168.84 | 4.95 | 5.07 | 0.017 | 4.188E+10 | 7 |
| **47** | 170.36 | 5.91 | 5.83 | 0.019 | 4.307E+10 | 7 |
| **48** | 172.19 | 5.59 | 5.35 | 0.017 | 4.435E+10 | 7 |
| **49** | 172.39 | 5.35 | 5.18 | 0.017 | 5.451E+10 | 7 |
| **50** | 158.88 | 6.98 | 6.99 | 0.026 | 3.920E+10 | 7 |
| **51** | 124.26 | 5.18 | 5.19 | 0.027 | 2.587E+10 | 7 |
| **52** | 171.74 | 7.04 | 7.15 | 0.023 | 4.285E+10 | 7 |
| **53** | 165.05 | 7.06 | 7.01 | 0.023 | 3.895E+10 | 6 |
| **54** | 169.08 | 5.42 | 5.49 | 0.018 | 4.018E+10 | 7 |
| **55** | 110.03 | 3.03 | 3.05 | 0.019 | 9.563E+09 | 7 |
| **56** | 128.84 | 3.74 | 3.66 | 0.018 | 4.225E+10 | 7 |
| **57** | 158.89 | 4.91 | 4.87 | 0.018 | 4.273E+10 | 7 |
| **58** | 101.18 | 3.34 | 3.21 | 0.022 | 2.903E+10 | 7 |
| **59** | 181.41 | 8.93 | 8.87 | 0.027 | 6.860E+09 | 7 |
| **60** | 135.78 | 4.86 | 4.96 | 0.023 | 3.543E+10 | 7 |

**Table S28.** $T_2$ relaxation times of **a$_4$CuMT** (N46) binding **$Cd^{2+}$**. The standard error for $T_2$ relaxation times was estimated using a Monte Carlo approach ($T_2$ MC SE) that combined the standard error for the $T_2$ fit ($T_2$ SE) and the standard error for fitting the initial intensity (M0 SE). The M0 SE is given for an intensity of one and is therefore a relative measure. The fitted absolute initial (delay = 0 ms) intensity (M0) is given in a separate column. The degrees of freedom (df) indicate the number of recorded data points (9 data points for a df of 7).

| | $T_2$ Relaxation [ms] | $T_2$ MC SE [ms] | $T_2$ SE [ms] | M0 SE [rel] | M0 [abs] | df |
|---|---|---|---|---|---|---|
| **1** | 69.10 | 2.42 | 2.38 | 0.027 | 3.287E+09 | 7 |
| **2** | 138.20 | 2.52 | 2.50 | 0.011 | 3.125E+10 | 7 |
| **3** | 193.07 | 8.89 | 8.91 | 0.024 | 6.904E+09 | 7 |
| **4** | 86.55 | 3.39 | 3.41 | 0.029 | 4.243E+09 | 7 |
| **5** | 28.89 | 2.04 | 1.96 | 0.065 | 2.080E+09 | 7 |
| **6** | 114.36 | 4.15 | 4.11 | 0.024 | 2.519E+10 | 7 |
| **7** | 156.95 | 3.64 | 3.61 | 0.013 | 2.687E+10 | 7 |
| **8** | 139.40 | 4.52 | 4.65 | 0.021 | 3.237E+10 | 7 |
| **9** | 58.53 | 4.35 | 4.37 | 0.060 | 1.192E+09 | 7 |
| **10** | 110.69 | 3.53 | 3.54 | 0.022 | 2.889E+10 | 7 |
| **11** | 139.97 | 4.53 | 4.53 | 0.020 | 3.255E+10 | 7 |
| **12** | 125.82 | 2.96 | 2.85 | 0.015 | 5.371E+09 | 7 |
| **13** | 117.36 | 7.43 | 7.30 | 0.041 | 2.144E+09 | 7 |
| **14** | 143.73 | 4.60 | 4.56 | 0.019 | 3.135E+10 | 7 |
| **15** | 188.64 | 9.26 | 9.00 | 0.026 | 4.666E+09 | 7 |
| **16** | 176.35 | 3.62 | 3.47 | 0.011 | 2.569E+10 | 7 |
| **17** | 121.94 | 5.69 | 5.47 | 0.029 | 3.389E+09 | 7 |
| **18** | 103.28 | 4.95 | 5.05 | 0.034 | 1.846E+09 | 7 |
| **19** | 95.06 | 2.29 | 2.25 | 0.017 | 2.145E+10 | 7 |
| **20** | 160.08 | 5.02 | 5.07 | 0.018 | 2.651E+10 | 7 |
| **21** | 45.58 | 18.83 | 12.66 | 0.237 | 7.408E+08 | 7 |
| **22** | 30.59 | 2.68 | 2.73 | 0.084 | 2.507E+09 | 7 |
| **23** | 161.69 | 6.96 | 6.86 | 0.025 | 2.936E+09 | 7 |
| **24** | 160.52 | 4.15 | 4.01 | 0.014 | 8.484E+09 | 7 |
| **25** | 121.67 | 2.40 | 2.41 | 0.013 | 1.501E+10 | 7 |
| **26** | 148.35 | 1.89 | 1.87 | 0.008 | 3.961E+10 | 7 |
| **27** | 161.12 | 3.44 | 3.37 | 0.012 | 1.583E+10 | 7 |
| **28** | 126.98 | 4.03 | 4.04 | 0.020 | 2.874E+10 | 7 |
| **29** | 91.17 | 3.29 | 3.28 | 0.026 | 1.948E+10 | 7 |
| **30** | 59.63 | 1.21 | 1.22 | 0.016 | 4.718E+09 | 7 |
| **31** | 132.61 | 5.05 | 4.87 | 0.023 | 1.871E+10 | 7 |
| **32** | 126.72 | 3.23 | 3.26 | 0.016 | 1.575E+10 | 7 |
| **33** | 154.78 | 4.41 | 4.29 | 0.016 | 4.990E+09 | 7 |
| **34** | 45.85 | 1.49 | 1.52 | 0.028 | 2.083E+10 | 7 |
| **35** | 174.44 | 2.93 | 2.98 | 0.010 | 4.045E+10 | 7 |
| **36** | 83.89 | 1.37 | 1.38 | 0.012 | 9.473E+09 | 7 |
| **37** | 146.30 | 2.94 | 2.97 | 0.012 | 4.309E+10 | 7 |
| **38** | 106.40 | 2.46 | 2.40 | 0.015 | 8.672E+09 | 7 |
| **39** | 97.28 | 2.10 | 2.09 | 0.015 | 8.929E+09 | 7 |
| **40** | 111.56 | 2.30 | 2.40 | 0.014 | 4.654E+10 | 7 |
| **41** | 92.74 | 3.42 | 3.30 | 0.025 | 1.597E+10 | 7 |
| **42** | 144.24 | 3.81 | 3.72 | 0.016 | 3.696E+10 | 7 |

**Table S29.** $T_2$ relaxation times of **$a_4$UnMT** (N41) binding **$Cd^{2+}$**. The standard error for $T_2$ relaxation times was estimated using a Monte Carlo approach ($T_2$ MC SE) that combined the standard error for the $T_2$ fit ($T_2$ SE) and the standard error for fitting the initial intensity (M0 SE). The M0 SE is given for an intensity of one and is therefore a relative measure. The fitted absolute initial (delay = 0 ms) intensity (M0) is given in a separate column. The degrees of freedom (df) indicate the number of recorded data points (8 data points for a df of 6).

| | $T_2$ Relaxation [ms] | $T_2$ MC SE [ms] | $T_2$ SE [ms] | M0 SE [rel] | M0 [abs] | df |
|---|---|---|---|---|---|---|
| **1** | 153.03 | 4.92 | 4.82 | 0.019 | 4.480E+09 | 6 |
| **2** | 114.14 | 7.95 | 7.32 | 0.043 | 1.101E+10 | 6 |
| **3** | 181.59 | 3.04 | 3.09 | 0.010 | 5.929E+09 | 6 |
| **4** | 175.82 | 4.84 | 4.84 | 0.016 | 4.216E+09 | 6 |
| **5** | 161.77 | 8.61 | 8.44 | 0.031 | 3.336E+09 | 6 |
| **6** | 155.13 | 11.52 | 11.28 | 0.044 | 3.477E+09 | 6 |
| **7** | 185.32 | 6.01 | 5.78 | 0.018 | 3.737E+09 | 6 |
| **8** | 109.71 | 6.13 | 6.05 | 0.037 | 4.234E+09 | 6 |
| **9** | 173.43 | 4.33 | 4.40 | 0.015 | 4.061E+09 | 6 |
| **10** | 144.27 | 7.22 | 7.10 | 0.031 | 1.054E+10 | 6 |
| **11** | 134.54 | 5.88 | 5.85 | 0.028 | 1.002E+10 | 6 |
| **12** | 110.61 | 4.18 | 4.13 | 0.025 | 1.435E+10 | 6 |
| **13** | 114.51 | 3.59 | 3.48 | 0.020 | 3.432E+09 | 6 |
| **14** | 184.16 | 5.78 | 5.67 | 0.017 | 4.170E+09 | 6 |
| **15** | 137.83 | 4.81 | 4.88 | 0.022 | 1.970E+10 | 6 |
| **16** | 106.05 | 3.94 | 3.89 | 0.025 | 5.570E+09 | 6 |
| **17** | 108.00 | 7.22 | 7.06 | 0.044 | 1.452E+10 | 6 |
| **18** | 189.66 | 2.16 | 2.20 | 0.006 | 4.814E+09 | 6 |
| **19** | 215.51 | 7.57 | 7.48 | 0.018 | 3.354E+09 | 6 |
| **20** | 74.32 | 2.65 | 2.55 | 0.025 | 2.832E+10 | 6 |
| **21** | 81.71 | 3.06 | 3.03 | 0.026 | 7.945E+09 | 6 |
| **22** | 202.80 | 5.32 | 5.05 | 0.013 | 4.927E+09 | 6 |
| **23** | 172.48 | 4.92 | 4.89 | 0.016 | 4.476E+09 | 6 |
| **24** | 209.07 | 8.02 | 8.02 | 0.020 | 4.183E+09 | 6 |
| **25** | 140.18 | 8.41 | 8.19 | 0.037 | 9.931E+09 | 6 |
| **26** | 172.11 | 7.31 | 7.31 | 0.025 | 4.204E+09 | 6 |
| **27** | 35.59 | 1.81 | 1.79 | 0.042 | 1.108E+10 | 6 |
| **28** | 129.51 | 7.06 | 7.05 | 0.035 | 3.380E+09 | 6 |
| **29** | 39.17 | 2.31 | 2.25 | 0.046 | 6.916E+09 | 6 |
| **30** | 137.14 | 6.70 | 6.40 | 0.030 | 1.794E+10 | 6 |
| **31** | 123.39 | 5.95 | 5.96 | 0.032 | 1.061E+10 | 6 |
| **32** | 148.95 | 3.73 | 3.76 | 0.016 | 4.757E+09 | 6 |
| **33** | 152.38 | 8.63 | 8.55 | 0.034 | 3.351E+09 | 6 |
| **34** | 108.52 | 4.65 | 4.50 | 0.028 | 1.175E+10 | 6 |
| **35** | 116.66 | 3.85 | 3.79 | 0.022 | 1.052E+10 | 6 |
| **36** | 167.72 | 9.25 | 8.90 | 0.031 | 3.594E+09 | 6 |
| **37** | 117.50 | 6.03 | 5.87 | 0.033 | 1.178E+10 | 6 |
| **38** | 178.00 | 5.87 | 5.50 | 0.018 | 4.226E+09 | 6 |
| **39** | 159.80 | 5.10 | 5.23 | 0.020 | 3.978E+09 | 6 |
| **40** | 172.88 | 7.17 | 7.09 | 0.024 | 3.765E+09 | 6 |
| **41** | 157.06 | 9.79 | 9.41 | 0.036 | 2.478E+09 | 6 |
| **42** | 64.72 | 5.07 | 4.96 | 0.056 | 1.406E+10 | 6 |
| **43** | 179.15 | 3.37 | 3.38 | 0.011 | 6.056E+09 | 6 |
| **44** | 164.51 | 2.77 | 2.83 | 0.010 | 3.794E+09 | 6 |
| **45** | 163.30 | 7.89 | 7.86 | 0.029 | 9.053E+09 | 6 |
| **46** | 181.14 | 8.31 | 8.20 | 0.026 | 3.527E+09 | 6 |
| **47** | 177.49 | 3.85 | 3.85 | 0.012 | 3.901E+09 | 6 |
| **48** | 74.40 | 2.99 | 2.93 | 0.028 | 1.331E+10 | 6 |
| **49** | 130.00 | 5.24 | 5.23 | 0.026 | 3.082E+10 | 6 |
| **50** | 135.96 | 5.79 | 5.57 | 0.026 | 4.394E+09 | 6 |
| **51** | 185.65 | 4.68 | 4.61 | 0.014 | 3.624E+09 | 6 |
| **52** | 95.85 | 5.58 | 5.47 | 0.040 | 4.056E+09 | 6 |
| **53** | 128.49 | 6.42 | 6.41 | 0.032 | 1.314E+10 | 6 |
| **54** | 143.21 | 5.72 | 5.67 | 0.025 | 3.288E+10 | 6 |
| **55** | 110.70 | 5.76 | 5.82 | 0.035 | 4.119E+09 | 6 |
| **56** | 122.77 | 5.75 | 5.69 | 0.030 | 2.230E+10 | 6 |
| **57** | 135.14 | 3.85 | 3.83 | 0.018 | 3.849E+09 | 6 |
| **58** | 172.51 | 9.05 | 8.77 | 0.029 | 4.001E+09 | 6 |
| **59** | 144.58 | 7.36 | 7.02 | 0.030 | 3.742E+09 | 6 |
| **60** | 142.00 | 8.96 | 8.78 | 0.039 | 2.244E+09 | 6 |
| **61** | 80.15 | 2.60 | 2.52 | 0.022 | 4.357E+09 | 6 |
| **62** | 39.17 | 2.27 | 2.25 | 0.046 | 6.916E+09 | 6 |

| | | | | | | |
|---|---|---|---|---|---|---|
| **63** | 165.80 | 5.62 | 5.45 | 0.019 | 4.065E+09 | 6 |
| **64** | 62.68 | 3.69 | 3.58 | 0.042 | 6.727E+09 | 6 |
| **65** | 161.94 | 7.72 | 7.55 | 0.028 | 7.450E+09 | 6 |
| **66** | 72.29 | 2.29 | 2.22 | 0.022 | 4.409E+09 | 6 |
| **67** | 135.81 | 5.41 | 5.52 | 0.026 | 2.504E+10 | 6 |
| **68** | 88.26 | 3.52 | 3.56 | 0.028 | 1.685E+10 | 6 |
| **69** | 175.98 | 7.15 | 7.02 | 0.023 | 4.295E+09 | 6 |
| **70** | 106.35 | 4.44 | 4.38 | 0.028 | 5.264E+09 | 6 |
| **71** | 126.95 | 3.83 | 3.83 | 0.020 | 3.076E+10 | 6 |

**Table S30.** $T_2$ relaxation times of **HpCdMT** binding **$Cd^{2+}$**. The standard error for $T_2$ relaxation times was estimated using a Monte Carlo approach ($T_2$ MC SE) that combined the standard error for the $T_2$ fit ($T_2$ SE) and the standard error for fitting the initial intensity (M0 SE). The M0 SE is given for an intensity of one and is therefore a relative measure. The fitted absolute initial (delay = 0 ms) intensity (M0) is given in a separate column. The degrees of freedom (df) indicate the number of recorded data points (9 data points for a df of 7).

| | $T_2$ Relaxation [ms] | $T_2$ MC SE [ms] | $T_2$ SE [ms] | M0 SE [rel] | M0 [abs] | df |
|---|---|---|---|---|---|---|
| **1** | 236.66 | 3.31 | 3.38 | 0.005 | 7.371E+05 | 7 |
| **2** | 213.05 | 14.38 | 13.93 | 0.023 | 4.348E+05 | 7 |
| **3** | 189.94 | 3.67 | 3.59 | 0.007 | 6.460E+05 | 7 |
| **4** | 135.18 | 6.21 | 6.31 | 0.021 | 3.316E+05 | 7 |
| **5** | 199.31 | 4.35 | 4.41 | 0.008 | 6.217E+05 | 7 |
| **6** | 173.20 | 4.60 | 4.62 | 0.011 | 5.102E+05 | 7 |
| **7** | 183.51 | 3.34 | 3.26 | 0.007 | 4.716E+05 | 7 |
| **8** | 150.82 | 3.31 | 3.22 | 0.009 | 4.378E+05 | 7 |
| **9** | 145.38 | 3.90 | 3.91 | 0.012 | 2.142E+05 | 7 |
| **10** | 185.57 | 3.80 | 3.90 | 0.008 | 5.188E+05 | 7 |
| **11** | 187.51 | 3.40 | 3.37 | 0.007 | 3.430E+05 | 7 |
| **12** | 150.45 | 4.34 | 4.24 | 0.012 | 4.201E+05 | 7 |
| **13** | 198.75 | 3.30 | 3.35 | 0.006 | 4.906E+05 | 7 |
| **14** | 131.17 | 3.46 | 3.45 | 0.012 | 1.866E+05 | 7 |
| **15** | 168.74 | 4.46 | 4.53 | 0.011 | 5.239E+05 | 7 |
| **16** | 178.56 | 4.18 | 4.22 | 0.009 | 5.844E+05 | 7 |
| **17** | 163.86 | 3.09 | 3.14 | 0.008 | 5.629E+05 | 7 |
| **18** | 174.32 | 3.82 | 3.95 | 0.009 | 4.279E+05 | 7 |
| **19** | 167.54 | 3.38 | 3.56 | 0.009 | 5.209E+05 | 7 |
| **20** | 178.57 | 4.25 | 4.28 | 0.009 | 5.480E+05 | 7 |
| **21** | 191.68 | 3.81 | 3.85 | 0.008 | 5.779E+05 | 7 |
| **22** | 207.05 | 3.71 | 3.79 | 0.007 | 5.689E+05 | 7 |
| **23** | 214.31 | 4.92 | 4.99 | 0.008 | 2.891E+05 | 7 |
| **24** | 194.53 | 3.94 | 3.93 | 0.008 | 5.069E+05 | 7 |
| **25** | 267.78 | 4.13 | 4.11 | 0.005 | 6.706E+05 | 7 |
| **26** | 183.84 | 3.19 | 3.14 | 0.007 | 6.639E+05 | 7 |
| **27** | 183.74 | 3.45 | 3.50 | 0.007 | 5.461E+05 | 7 |
| **28** | 179.03 | 3.14 | 3.12 | 0.007 | 5.544E+05 | 7 |
| **29** | 186.21 | 2.77 | 2.78 | 0.006 | 8.446E+05 | 7 |
| **30** | 132.95 | 5.07 | 5.15 | 0.017 | 4.815E+05 | 7 |
| **31** | 142.63 | 5.13 | 5.26 | 0.016 | 3.798E+05 | 7 |
| **32** | 173.70 | 3.49 | 3.41 | 0.008 | 3.240E+05 | 7 |
| **33** | 136.77 | 4.89 | 5.17 | 0.017 | 5.513E+05 | 7 |
| **34** | 187.78 | 4.90 | 4.82 | 0.010 | 5.112E+05 | 7 |
| **35** | 193.90 | 3.06 | 3.06 | 0.006 | 4.498E+05 | 7 |
| **36** | 206.97 | 2.76 | 2.81 | 0.005 | 5.158E+05 | 7 |
| **37** | 181.44 | 5.68 | 5.49 | 0.012 | 1.902E+05 | 7 |
| **38** | 239.31 | 5.46 | 5.47 | 0.008 | 2.375E+05 | 7 |
| **39** | 162.22 | 4.45 | 4.56 | 0.012 | 5.584E+05 | 7 |
| **40** | 193.78 | 3.97 | 4.02 | 0.008 | 5.650E+05 | 7 |
| **41** | 222.85 | 12.23 | 12.16 | 0.019 | 9.205E+04 | 7 |
| **42** | 203.60 | 4.00 | 3.99 | 0.007 | 5.332E+05 | 7 |
| **43** | 169.24 | 3.88 | 3.91 | 0.009 | 5.276E+05 | 7 |
| **44** | 189.70 | 3.15 | 3.06 | 0.006 | 4.239E+05 | 7 |
| **45** | 189.03 | 3.34 | 3.40 | 0.007 | 5.662E+05 | 7 |
| **46** | 135.28 | 3.54 | 3.65 | 0.012 | 3.742E+05 | 7 |
| **47** | 209.11 | 3.38 | 3.36 | 0.006 | 5.697E+05 | 7 |
| **48** | 177.53 | 9.24 | 8.89 | 0.020 | 7.016E+05 | 7 |
| **49** | 109.17 | 3.53 | 3.58 | 0.016 | 3.729E+05 | 7 |
| **50** | 170.76 | 3.20 | 3.29 | 0.008 | 5.945E+05 | 7 |
| **51** | 178.59 | 3.71 | 3.68 | 0.008 | 4.544E+05 | 7 |
| **52** | 175.73 | 4.41 | 4.31 | 0.010 | 4.553E+05 | 7 |
| **53** | 170.21 | 3.99 | 3.97 | 0.009 | 3.628E+05 | 7 |
| **54** | 190.27 | 2.68 | 2.75 | 0.005 | 5.228E+05 | 7 |
| **55** | 191.09 | 3.62 | 3.71 | 0.007 | 4.322E+05 | 7 |
| **56** | 122.29 | 6.74 | 6.60 | 0.025 | 5.173E+05 | 7 |
| **57** | 126.57 | 4.95 | 4.94 | 0.018 | 4.315E+05 | 7 |
| **58** | 189.99 | 3.15 | 3.11 | 0.006 | 2.452E+05 | 7 |
| **59** | 125.60 | 4.69 | 4.93 | 0.018 | 3.936E+05 | 7 |

**Table S31.** $T_2$ relaxation times of **HpCuMT** binding **$Cd^{2+}$**. The standard error for $T_2$ relaxation times was estimated using a Monte Carlo approach ($T_2$ MC SE) that combined the standard error for the $T_2$ fit ($T_2$ SE) and the standard error for fitting the initial intensity (M0 SE). The M0 SE is given for an intensity of one and is therefore a relative measure. The fitted absolute initial (delay = 0 ms) intensity (M0) is given in a separate column. The degrees of freedom (df) indicate the number of recorded data points (8 data points for a df of 6).

| | $T_2$ Relaxation [ms] | $T_2$ MC SE [ms] | $T_2$ SE [ms] | M0 SE [rel] | M0 [abs] | df |
|---|---|---|---|---|---|---|
| **1** | 140.81 | 4.61 | 4.64 | 0.019 | 5.528E+09 | 6 |
| **2** | 99.91 | 3.42 | 3.44 | 0.023 | 2.892E+09 | 6 |
| **3** | 81.48 | 4.39 | 4.23 | 0.037 | 2.416E+09 | 6 |
| **4** | 120.80 | 4.29 | 4.31 | 0.022 | 2.729E+09 | 6 |
| **5** | 135.10 | 5.67 | 5.64 | 0.025 | 4.398E+09 | 6 |
| **6** | 137.13 | 4.90 | 4.91 | 0.021 | 6.090E+09 | 6 |
| **7** | 98.61 | 5.95 | 6.03 | 0.041 | 2.490E+09 | 6 |
| **8** | 107.34 | 5.48 | 5.36 | 0.032 | 1.326E+09 | 6 |
| **9** | 166.89 | 11.47 | 11.38 | 0.036 | 1.751E+09 | 6 |
| **10** | 81.56 | 3.92 | 3.90 | 0.034 | 1.223E+09 | 6 |
| **11** | 146.27 | 4.61 | 4.65 | 0.018 | 5.204E+09 | 6 |
| **12** | 108.88 | 2.49 | 2.44 | 0.014 | 4.033E+09 | 6 |
| **13** | 169.45 | 7.09 | 7.13 | 0.022 | 4.922E+09 | 6 |
| **14** | 63.40 | 3.24 | 3.23 | 0.040 | 4.211E+09 | 6 |
| **15** | 98.76 | 4.63 | 4.41 | 0.030 | 3.111E+09 | 6 |
| **16** | 162.40 | 4.49 | 4.52 | 0.015 | 4.495E+09 | 6 |
| **17** | 106.57 | 2.62 | 2.70 | 0.017 | 2.020E+09 | 6 |
| **18** | 109.38 | 3.31 | 3.31 | 0.019 | 5.634E+09 | 6 |
| **19** | 132.89 | 5.10 | 5.20 | 0.023 | 4.314E+09 | 6 |
| **20** | 103.66 | 4.99 | 5.01 | 0.032 | 2.322E+09 | 6 |
| **21** | 120.56 | 7.99 | 7.79 | 0.040 | 1.163E+09 | 6 |
| **22** | 122.22 | 4.93 | 5.07 | 0.025 | 1.450E+09 | 6 |
| **23** | 90.64 | 5.78 | 5.61 | 0.043 | 1.206E+09 | 6 |
| **24** | 151.39 | 9.20 | 8.95 | 0.033 | 2.594E+09 | 6 |
| **25** | 154.68 | 5.74 | 5.52 | 0.020 | 5.396E+09 | 6 |
| **26** | 149.00 | 4.26 | 4.19 | 0.016 | 4.628E+09 | 6 |
| **27** | 57.92 | 3.17 | 3.14 | 0.043 | 1.571E+09 | 6 |

**Table S32.** $T_2$ relaxation times of **HpUnMT1** binding **$Cd^{2+}$**. The standard error for $T_2$ relaxation times was estimated using a Monte Carlo approach ($T_2$ MC SE) that combined the standard error for the $T_2$ fit ($T_2$ SE) and the standard error for fitting the initial intensity (M0 SE). The M0 SE is given for an intensity of one and is therefore a relative measure. The fitted absolute initial (delay = 0 ms) intensity (M0) is given in a separate column. The degrees of freedom (df) indicate the number of recorded data points (9 data points for a df of 7).

| | $T_2$ Relaxation [ms] | $T_2$ MC SE [ms] | $T_2$ SE [ms] | M0 SE [rel] | M0 [abs] | df |
|---|---|---|---|---|---|---|
| **1** | 119.44 | 3.44 | 3.38 | 0.019 | 1.006E+10 | 7 |
| **2** | 147.52 | 4.30 | 4.34 | 0.018 | 3.592E+09 | 7 |
| **3** | 139.09 | 7.35 | 7.36 | 0.033 | 1.167E+09 | 7 |
| **4** | 142.77 | 19.99 | 19.29 | 0.082 | 5.505E+08 | 7 |
| **5** | 215.43 | 22.59 | 20.80 | 0.048 | 1.041E+09 | 7 |
| **6** | 122.94 | 22.35 | 20.68 | 0.109 | 5.572E+08 | 7 |
| **7** | 131.52 | 6.08 | 5.91 | 0.028 | 3.963E+09 | 7 |
| **8** | 136.17 | 6.41 | 6.35 | 0.029 | 2.208E+09 | 7 |
| **9** | 207.68 | 19.49 | 19.27 | 0.047 | 7.572E+08 | 7 |
| **10** | 154.16 | 3.16 | 3.18 | 0.012 | 1.159E+10 | 7 |
| **11** | 131.12 | 4.31 | 4.37 | 0.021 | 5.025E+09 | 7 |
| **12** | 161.97 | 3.59 | 3.63 | 0.013 | 1.580E+10 | 7 |
| **13** | 151.50 | 8.57 | 8.33 | 0.033 | 2.754E+09 | 7 |
| **14** | 135.81 | 4.26 | 4.17 | 0.019 | 1.134E+10 | 7 |
| **15** | 153.00 | 3.82 | 3.81 | 0.015 | 8.754E+09 | 7 |
| **16** | 148.90 | 18.69 | 18.23 | 0.073 | 6.700E+08 | 7 |
| **17** | 135.31 | 3.73 | 3.88 | 0.018 | 1.097E+10 | 7 |
| **18** | 136.87 | 5.45 | 5.40 | 0.025 | 3.856E+09 | 7 |
| **19** | 127.16 | 6.01 | 5.91 | 0.030 | 1.800E+09 | 7 |
| **20** | 159.75 | 4.74 | 4.80 | 0.017 | 6.169E+09 | 7 |
| **21** | 147.51 | 4.44 | 4.20 | 0.017 | 8.637E+09 | 7 |
| **22** | 141.31 | 24.97 | 22.20 | 0.096 | 6.295E+08 | 7 |
| **23** | 79.84 | 2.47 | 2.55 | 0.024 | 1.097E+10 | 7 |
| **24** | 147.82 | 3.86 | 3.85 | 0.016 | 8.629E+09 | 7 |
| **25** | 142.67 | 4.50 | 4.38 | 0.019 | 6.355E+09 | 7 |
| **26** | 130.21 | 3.66 | 3.51 | 0.017 | 6.051E+09 | 7 |
| **27** | 125.88 | 5.07 | 5.01 | 0.026 | 3.273E+09 | 7 |
| **28** | 112.30 | 4.14 | 4.20 | 0.025 | 4.513E+09 | 7 |
| **29** | 128.18 | 3.10 | 3.12 | 0.016 | 1.031E+10 | 7 |
| **30** | 145.47 | 14.87 | 14.31 | 0.060 | 6.560E+08 | 7 |
| **31** | 178.22 | 17.26 | 17.07 | 0.053 | 1.075E+09 | 7 |
| **32** | 127.86 | 4.29 | 4.23 | 0.021 | 5.388E+09 | 7 |
| **33** | 102.94 | 4.03 | 4.16 | 0.028 | 6.066E+09 | 7 |
| **34** | 103.09 | 22.43 | 20.68 | 0.139 | 6.455E+08 | 7 |
| **35** | 170.90 | 12.22 | 11.83 | 0.039 | 8.041E+08 | 7 |
| **36** | 141.09 | 3.84 | 3.92 | 0.017 | 6.373E+09 | 7 |
| **37** | 105.79 | 11.37 | 11.16 | 0.072 | 6.638E+08 | 7 |
| **38** | 71.83 | 9.20 | 9.05 | 0.097 | 8.017E+08 | 7 |
| **39** | 81.14 | 2.70 | 2.66 | 0.024 | 9.746E+09 | 7 |
| **40** | 187.38 | 6.20 | 6.03 | 0.017 | 5.091E+09 | 7 |
| **41** | 161.52 | 8.43 | 8.53 | 0.031 | 2.507E+09 | 7 |
| **42** | 194.69 | 17.09 | 16.76 | 0.045 | 1.728E+09 | 7 |
| **43** | 137.78 | 4.05 | 3.88 | 0.017 | 3.091E+09 | 7 |
| **44** | 109.20 | 11.72 | 11.93 | 0.074 | 8.199E+08 | 7 |
| **45** | 207.42 | 10.57 | 10.53 | 0.026 | 2.723E+09 | 7 |
| **46** | 44.61 | 3.51 | 3.53 | 0.068 | 2.072E+09 | 7 |
| **47** | 150.47 | 3.52 | 3.67 | 0.015 | 4.638E+09 | 7 |
| **48** | 189.99 | 18.53 | 17.21 | 0.048 | 7.655E+08 | 7 |
| **49** | 198.98 | 14.05 | 14.16 | 0.037 | 9.908E+08 | 7 |
| **50** | 89.97 | 5.70 | 5.98 | 0.048 | 1.512E+09 | 7 |
| **51** | 88.75 | 2.71 | 2.75 | 0.022 | 1.293E+09 | 7 |
| **52** | 170.61 | 14.77 | 14.33 | 0.047 | 9.474E+08 | 7 |
| **53** | 200.05 | 16.36 | 16.51 | 0.043 | 8.317E+08 | 7 |
| **54** | 89.19 | 4.82 | 4.84 | 0.039 | 3.149E+09 | 7 |
| **55** | 128.76 | 14.13 | 13.55 | 0.067 | 6.944E+08 | 7 |

**Table S33.** $T_2$ relaxation times of **HpUnMT2** binding **$Cd^{2+}$**. The standard error for $T_2$ relaxation times was estimated using a Monte Carlo approach ($T_2$ MC SE) that combined the standard error for the $T_2$ fit ($T_2$ SE) and the standard error for fitting the initial intensity (M0 SE). The M0 SE is given for an intensity of one and is therefore a relative measure. The fitted absolute initial (delay = 0 ms) intensity (M0) is given in a separate column. The degrees of freedom (df) indicate the number of recorded data points (9 data points for a df of 7).

| | $T_2$ Relaxation [ms] | $T_2$ MC SE [ms] | $T_2$ SE [ms] | M0 SE [rel] | M0 [abs] | df |
|---|---|---|---|---|---|---|
| **1** | 129.23 | 3.80 | 3.88 | 0.019 | 1.181E+10 | 7 |
| **2** | 128.83 | 2.28 | 2.34 | 0.012 | 2.084E+10 | 7 |
| **3** | 127.44 | 11.36 | 11.08 | 0.056 | 1.186E+09 | 7 |
| **4** | 224.25 | 11.18 | 11.40 | 0.025 | 9.212E+08 | 7 |
| **5** | 136.03 | 4.03 | 4.00 | 0.018 | 8.619E+09 | 7 |
| **6** | 137.62 | 3.69 | 3.84 | 0.017 | 1.798E+10 | 7 |
| **7** | 145.52 | 3.28 | 3.43 | 0.014 | 2.014E+10 | 7 |
| **8** | 198.03 | 5.84 | 5.74 | 0.015 | 7.304E+09 | 7 |
| **9** | 147.91 | 3.31 | 3.53 | 0.014 | 1.572E+10 | 7 |
| **10** | 157.12 | 3.75 | 3.74 | 0.014 | 1.325E+10 | 7 |
| **11** | 114.50 | 22.18 | 19.54 | 0.114 | 6.531E+09 | 7 |
| **12** | 193.31 | 23.54 | 22.47 | 0.061 | 8.060E+08 | 7 |
| **13** | 133.82 | 15.66 | 15.79 | 0.074 | 9.412E+08 | 7 |
| **14** | 112.47 | 5.38 | 5.37 | 0.032 | 2.106E+09 | 7 |
| **15** | 112.94 | 4.45 | 4.35 | 0.026 | 1.919E+10 | 7 |
| **16** | 155.12 | 20.95 | 20.47 | 0.078 | 8.830E+08 | 7 |
| **17** | 114.31 | 6.98 | 6.69 | 0.039 | 1.070E+09 | 7 |
| **18** | 124.26 | 5.13 | 4.95 | 0.026 | 1.606E+09 | 7 |
| **19** | 114.87 | 2.78 | 2.79 | 0.016 | 7.492E+09 | 7 |
| **20** | 87.70 | 8.27 | 7.54 | 0.063 | 1.453E+09 | 7 |
| **21** | 125.13 | 3.82 | 3.84 | 0.020 | 1.238E+10 | 7 |
| **22** | 157.86 | 3.55 | 3.62 | 0.013 | 1.938E+09 | 7 |
| **23** | 157.02 | 3.71 | 3.58 | 0.013 | 2.531E+10 | 7 |
| **24** | 150.89 | 3.89 | 3.84 | 0.015 | 2.085E+10 | 7 |
| **25** | 136.13 | 4.81 | 4.92 | 0.023 | 5.815E+09 | 7 |
| **26** | 101.98 | 3.77 | 3.73 | 0.025 | 3.728E+09 | 7 |
| **27** | 112.96 | 7.07 | 7.09 | 0.042 | 1.569E+09 | 7 |
| **28** | 131.50 | 4.41 | 4.38 | 0.021 | 2.945E+09 | 7 |
| **29** | 87.50 | 5.41 | 5.36 | 0.045 | 9.979E+08 | 7 |
| **30** | 131.23 | 3.86 | 3.92 | 0.019 | 2.051E+10 | 7 |
| **31** | 140.49 | 3.43 | 3.42 | 0.015 | 1.349E+10 | 7 |
| **32** | 24.33 | 24.90 | 6.64 | 0.282 | 8.414E+08 | 7 |
| **33** | 151.78 | 17.33 | 16.14 | 0.063 | 8.812E+08 | 7 |
| **34** | 84.12 | 2.60 | 2.68 | 0.023 | 2.052E+10 | 7 |
| **35** | 99.11 | 6.30 | 6.24 | 0.044 | 1.814E+09 | 7 |
| **36** | 186.08 | 22.68 | 22.09 | 0.064 | 7.251E+08 | 7 |
| **37** | 117.61 | 3.22 | 3.10 | 0.017 | 1.654E+10 | 7 |
| **38** | 102.31 | 4.55 | 4.58 | 0.031 | 2.875E+09 | 7 |
| **39** | 156.01 | 5.12 | 5.12 | 0.019 | 1.850E+09 | 7 |
| **40** | 106.71 | 3.71 | 3.69 | 0.024 | 1.307E+10 | 7 |
| **41** | 177.52 | 7.64 | 7.43 | 0.023 | 2.151E+09 | 7 |
| **42** | 107.16 | 11.17 | 10.66 | 0.068 | 4.898E+08 | 7 |
| **43** | 111.65 | 7.43 | 7.21 | 0.043 | 8.429E+08 | 7 |
| **44** | 151.38 | 4.43 | 4.37 | 0.017 | 3.147E+09 | 7 |
| **45** | 124.70 | 15.83 | 15.61 | 0.081 | 7.068E+08 | 7 |
| **46** | 135.48 | 18.19 | 17.43 | 0.080 | 9.287E+08 | 7 |
| **47** | 117.82 | 5.38 | 5.51 | 0.031 | 2.235E+09 | 7 |
| **48** | 104.63 | 5.84 | 5.80 | 0.038 | 1.270E+09 | 7 |
| **49** | 117.64 | 8.43 | 8.61 | 0.048 | 1.198E+09 | 7 |
| **50** | 164.71 | 12.84 | 12.44 | 0.043 | 1.313E+09 | 7 |
| **51** | 41.04 | 1.82 | 1.78 | 0.038 | 5.722E+09 | 7 |
| **52** | 91.18 | 2.77 | 2.92 | 0.023 | 5.878E+09 | 7 |
| **53** | 125.05 | 6.89 | 6.84 | 0.035 | 1.000E+09 | 7 |
| **54** | 40.77 | 8.20 | 7.37 | 0.158 | 7.237E+08 | 7 |
| **55** | 140.25 | 5.52 | 5.61 | 0.025 | 1.800E+10 | 7 |
| **56** | 119.36 | 7.96 | 7.73 | 0.042 | 1.656E+09 | 7 |
| **57** | 167.03 | 13.29 | 13.05 | 0.044 | 9.676E+08 | 7 |
| **58** | 124.63 | 7.81 | 7.82 | 0.040 | 9.434E+08 | 7 |
| **59** | 115.64 | 6.36 | 6.16 | 0.035 | 2.816E+09 | 7 |
| **60** | 180.60 | 2.66 | 2.73 | 0.008 | 3.707E+09 | 7 |
| **61** | 191.87 | 7.91 | 7.93 | 0.022 | 5.752E+09 | 7 |
| **62** | 123.36 | 4.79 | 4.67 | 0.025 | 7.891E+09 | 7 |

| | | | | | | |
|---|---|---|---|---|---|---|
| **63** | 141.08 | 15.16 | 15.06 | 0.066 | 1.342E+09 | 7 |
| **64** | 92.36 | 5.58 | 5.41 | 0.042 | 1.503E+09 | 7 |
| **65** | 178.90 | 13.30 | 13.04 | 0.040 | 1.649E+09 | 7 |
| **66** | 57.52 | 1.91 | 1.89 | 0.027 | 1.441E+09 | 7 |
| **67** | 126.71 | 3.47 | 3.48 | 0.018 | 1.192E+10 | 7 |

**Table S34.** $T_2$ relaxation times of **a$_1$CdMT** (N20) binding **$Cu^+$**. The standard error for $T_2$ relaxation times was estimated using a Monte Carlo approach ($T_2$ MC SE) that combined the standard error for the $T_2$ fit ($T_2$ SE) and the standard error for fitting the initial intensity (M0 SE). The M0 SE is given for an intensity of one and is therefore a relative measure. The fitted absolute initial (delay = 0 ms) intensity (M0) is given in a separate column. The degrees of freedom (df) indicate the number of recorded data points (9 data points for a df of 7).

| | $T_2$ Relaxation [ms] | $T_2$ MC SE [ms] | $T_2$ SE [ms] | M0 SE [rel] | M0 [abs] | df |
|---|---|---|---|---|---|---|
| **1** | 98.17 | 4.49 | 4.36 | 0.031 | 1.81E+09 | 7 |
| **2** | 100.98 | 2.61 | 2.65 | 0.018 | 2.56E+09 | 7 |
| **3** | 101.59 | 16.27 | 15.30 | 0.105 | 1.06E+09 | 7 |
| **4** | 119.73 | 7.26 | 7.22 | 0.039 | 4.50E+09 | 7 |
| **5** | 48.71 | 13.07 | 9.98 | 0.172 | 8.29E+08 | 7 |
| **6** | 94.89 | 5.66 | 5.68 | 0.042 | 5.19E+08 | 7 |
| **7** | 143.91 | 12.44 | 12.36 | 0.052 | 6.16E+08 | 7 |
| **8** | 167.36 | 5.12 | 5.14 | 0.017 | 5.88E+09 | 7 |
| **9** | 130.02 | 11.97 | 11.49 | 0.056 | 1.26E+09 | 7 |
| **10** | 140.21 | 10.55 | 10.11 | 0.044 | 3.10E+09 | 7 |
| **11** | 175.46 | 11.09 | 11.55 | 0.037 | 1.02E+09 | 7 |
| **12** | 131.79 | 22.84 | 19.76 | 0.095 | 5.73E+08 | 7 |
| **13** | 149.60 | 5.32 | 5.44 | 0.022 | 4.84E+09 | 7 |
| **14** | 149.63 | 11.38 | 11.14 | 0.045 | 2.15E+09 | 7 |
| **15** | 168.34 | 12.44 | 12.15 | 0.041 | 1.70E+09 | 7 |
| **16** | 122.35 | 15.40 | 14.18 | 0.075 | 8.46E+08 | 7 |
| **17** | 182.30 | 16.80 | 16.08 | 0.048 | 1.22E+09 | 7 |
| **18** | 107.93 | 5.26 | 5.23 | 0.033 | 2.63E+09 | 7 |
| **19** | 161.37 | 15.25 | 15.16 | 0.054 | 9.68E+08 | 7 |
| **20** | 150.79 | 5.92 | 5.99 | 0.024 | 4.75E+09 | 7 |
| **21** | 134.81 | 11.45 | 11.11 | 0.052 | 1.11E+09 | 7 |
| **22** | 161.74 | 6.41 | 6.33 | 0.023 | 5.52E+09 | 7 |
| **23** | 52.35 | 13.86 | 13.01 | 0.206 | 8.70E+08 | 7 |
| **24** | 118.67 | 6.67 | 6.55 | 0.036 | 2.35E+09 | 7 |
| **25** | 115.57 | 4.87 | 4.78 | 0.027 | 6.87E+09 | 7 |
| **26** | 143.23 | 19.18 | 18.67 | 0.079 | 7.61E+08 | 7 |
| **27** | 50.65 | 2.61 | 2.51 | 0.041 | 2.37E+09 | 7 |
| **28** | 100.09 | 6.00 | 6.04 | 0.042 | 3.91E+09 | 7 |
| **29** | 167.73 | 3.77 | 3.73 | 0.013 | 6.26E+09 | 7 |
| **30** | 94.07 | 16.26 | 15.23 | 0.115 | 5.94E+08 | 7 |
| **31** | 151.37 | 13.03 | 12.74 | 0.050 | 1.30E+09 | 7 |
| **32** | 111.07 | 22.35 | 19.81 | 0.120 | 8.84E+08 | 7 |
| **33** | 163.09 | 7.27 | 6.89 | 0.024 | 1.43E+09 | 7 |
| **34** | 106.66 | 19.42 | 16.06 | 0.103 | 5.87E+08 | 7 |
| **35** | 223.10 | 13.25 | 13.12 | 0.029 | 1.44E+09 | 7 |
| **36** | 114.57 | 6.75 | 6.73 | 0.039 | 2.71E+09 | 7 |
| **37** | 101.94 | 4.96 | 4.81 | 0.033 | 3.72E+09 | 7 |
| **38** | 90.88 | 5.86 | 5.62 | 0.045 | 2.43E+09 | 7 |
| **39** | 77.04 | 16.98 | 16.12 | 0.158 | 6.66E+08 | 7 |
| **40** | 102.92 | 13.45 | 13.07 | 0.088 | 8.24E+08 | 7 |
| **41** | 164.45 | 8.65 | 8.97 | 0.031 | 2.85E+09 | 7 |
| **42** | 142.22 | 6.78 | 6.69 | 0.029 | 5.09E+09 | 7 |
| **43** | 184.95 | 3.78 | 3.84 | 0.011 | 5.93E+09 | 7 |
| **44** | 116.06 | 8.48 | 8.27 | 0.047 | 1.12E+09 | 7 |
| **45** | 143.05 | 19.12 | 18.37 | 0.078 | 1.08E+09 | 7 |
| **46** | 168.27 | 5.01 | 5.00 | 0.017 | 4.59E+09 | 7 |
| **47** | 102.64 | 16.78 | 16.95 | 0.114 | 8.78E+08 | 7 |
| **48** | 85.17 | 8.28 | 7.75 | 0.067 | 9.13E+08 | 7 |
| **49** | 118.75 | 18.88 | 18.14 | 0.100 | 9.68E+08 | 7 |
| **50** | 132.26 | 10.29 | 10.01 | 0.048 | 2.26E+09 | 7 |

**Table S35.** $T_2$ relaxation times of **a₂CuMT** (N36) binding **$Cu^+$**. The standard error for $T_2$ relaxation times was estimated using a Monte Carlo approach ($T_2$ MC SE) that combined the standard error for the $T_2$ fit ($T_2$ SE) and the standard error for fitting the initial intensity (M0 SE). The M0 SE is given for an intensity of one and is therefore a relative measure. The fitted absolute initial (delay = 0 ms) intensity (M0) is given in a separate column. The degrees of freedom (df) indicate the number of recorded data points (9 data points for a df of 7).

| | $T_2$ Relaxation [ms] | $T_2$ MC SE [ms] | $T_2$ SE [ms] | M0 SE [rel] | M0 [abs] | df |
|---|---|---|---|---|---|---|
| **1** | 29.59 | 7.62 | 6.84 | 0.221 | 2.243E+10 | 7 |
| **2** | 109.69 | 10.31 | 9.96 | 0.061 | 4.164E+10 | 7 |
| **3** | 148.59 | 8.88 | 8.31 | 0.034 | 5.690E+10 | 7 |
| **4** | 172.98 | 8.26 | 7.99 | 0.026 | 1.917E+11 | 7 |
| **5** | 170.27 | 5.76 | 5.70 | 0.019 | 1.270E+11 | 7 |
| **6** | 119.14 | 7.57 | 7.30 | 0.040 | 3.644E+10 | 7 |
| **7** | 82.55 | 14.99 | 14.02 | 0.126 | 2.368E+10 | 7 |
| **8** | 34.95 | 9.34 | 7.76 | 0.202 | 2.164E+10 | 7 |
| **9** | 112.22 | 14.55 | 13.51 | 0.081 | 1.927E+10 | 7 |
| **10** | 159.90 | 5.64 | 5.66 | 0.021 | 1.647E+11 | 7 |
| **11** | 107.57 | 7.89 | 7.69 | 0.049 | 2.099E+10 | 7 |
| **12** | 100.81 | 15.89 | 14.39 | 0.099 | 2.788E+10 | 7 |
| **13** | 104.93 | 12.54 | 12.25 | 0.080 | 2.373E+10 | 7 |
| **14** | 118.76 | 14.00 | 13.92 | 0.077 | 3.202E+10 | 7 |
| **15** | 121.64 | 6.24 | 6.10 | 0.033 | 4.866E+10 | 7 |
| **16** | 157.71 | 8.67 | 8.84 | 0.033 | 4.770E+10 | 7 |
| **17** | 143.43 | 3.91 | 3.76 | 0.016 | 1.567E+11 | 7 |
| **18** | 119.70 | 6.11 | 5.89 | 0.032 | 6.407E+10 | 7 |
| **19** | 110.49 | 7.85 | 7.76 | 0.047 | 2.696E+10 | 7 |
| **20** | 70.64 | 8.31 | 7.30 | 0.080 | 3.450E+10 | 7 |
| **21** | 134.99 | 7.91 | 7.97 | 0.037 | 1.162E+11 | 7 |
| **22** | 184.24 | 8.13 | 7.80 | 0.023 | 2.622E+11 | 7 |
| **23** | 200.42 | 17.62 | 16.12 | 0.042 | 7.748E+10 | 7 |
| **24** | 110.83 | 7.22 | 7.07 | 0.043 | 4.030E+10 | 7 |
| **25** | 137.57 | 14.56 | 14.36 | 0.065 | 1.602E+10 | 7 |
| **26** | 91.75 | 14.97 | 13.95 | 0.109 | 2.427E+10 | 7 |
| **27** | 140.44 | 5.24 | 5.40 | 0.024 | 1.100E+11 | 7 |
| **28** | 93.75 | 17.44 | 15.01 | 0.114 | 2.449E+10 | 7 |
| **29** | 104.32 | 5.70 | 5.75 | 0.038 | 5.686E+10 | 7 |
| **30** | 182.44 | 10.24 | 9.83 | 0.029 | 5.904E+10 | 7 |
| **31** | 106.11 | 10.88 | 10.41 | 0.067 | 3.470E+10 | 7 |
| **32** | 107.10 | 8.04 | 7.85 | 0.050 | 4.293E+10 | 7 |
| **33** | 53.18 | 2.45 | 2.43 | 0.038 | 1.100E+11 | 7 |
| **34** | 114.06 | 12.30 | 11.65 | 0.068 | 2.919E+10 | 7 |
| **35** | 21.33 | 4.22 | 3.45 | 0.178 | 5.261E+10 | 7 |
| **36** | 124.78 | 10.13 | 10.46 | 0.054 | 3.742E+10 | 7 |
| **37** | 86.13 | 7.65 | 7.33 | 0.062 | 2.534E+10 | 7 |
| **38** | 152.78 | 5.06 | 5.07 | 0.020 | 2.741E+11 | 7 |
| **39** | 44.35 | 2.15 | 2.13 | 0.041 | 6.162E+10 | 7 |
| **40** | 68.34 | 3.79 | 3.61 | 0.041 | 5.766E+10 | 7 |
| **41** | 65.24 | 7.82 | 7.40 | 0.089 | 1.328E+10 | 7 |
| **42** | 69.49 | 4.21 | 3.98 | 0.044 | 2.605E+10 | 7 |
| **43** | 180.28 | 6.12 | 5.94 | 0.018 | 2.198E+11 | 7 |
| **44** | 154.86 | 7.04 | 7.13 | 0.027 | 2.196E+11 | 7 |
| **45** | 160.77 | 5.75 | 5.84 | 0.021 | 1.330E+11 | 7 |
| **46** | 63.63 | 2.35 | 2.32 | 0.029 | 6.603E+10 | 7 |
| **47** | 96.07 | 10.47 | 9.86 | 0.073 | 3.850E+10 | 7 |
| **48** | 114.65 | 4.33 | 4.39 | 0.025 | 9.254E+10 | 7 |
| **49** | 176.73 | 5.20 | 5.14 | 0.016 | 2.559E+11 | 7 |
| **50** | 110.19 | 3.29 | 3.31 | 0.020 | 9.904E+10 | 7 |
| **51** | 137.35 | 3.96 | 3.93 | 0.018 | 1.583E+11 | 7 |

**Table S36.** $T_2$ relaxation times of **$a_3$CuMT1** (N40) binding **$Cu^+$**. The standard error for $T_2$ relaxation times was estimated using a Monte Carlo approach ($T_2$ MC SE) that combined the standard error for the $T_2$ fit ($T_2$ SE) and the standard error for fitting the initial intensity (M0 SE). The M0 SE is given for an intensity of one and is therefore a relative measure. The fitted absolute initial (delay = 0 ms) intensity (M0) is given in a separate column. The degrees of freedom (df) indicate the number of recorded data points (9 data points for a df of 7).

| | $T_2$ Relaxation [ms] | $T_2$ MC SE [ms] | $T_2$ SE [ms] | M0 SE [rel] | M0 [abs] | df |
|---|---|---|---|---|---|---|
| **1** | 95.26 | 3.64 | 3.66 | 0.027 | 2.470E+09 | 7 |
| **2** | 191.87 | 16.35 | 15.59 | 0.043 | 2.318E+09 | 7 |
| **3** | 127.21 | 6.22 | 6.40 | 0.032 | 3.583E+09 | 7 |
| **4** | 138.25 | 6.58 | 6.71 | 0.030 | 6.033E+09 | 7 |
| **5** | 156.24 | 4.23 | 4.26 | 0.016 | 6.963E+09 | 7 |
| **6** | 144.36 | 4.82 | 4.89 | 0.021 | 4.186E+09 | 7 |
| **7** | 181.52 | 8.43 | 8.61 | 0.026 | 5.725E+09 | 7 |
| **8** | 140.85 | 6.97 | 7.08 | 0.031 | 3.506E+09 | 7 |
| **9** | 162.25 | 4.33 | 4.36 | 0.015 | 7.643E+09 | 7 |
| **10** | 112.63 | 3.39 | 3.44 | 0.020 | 5.215E+09 | 7 |
| **11** | 74.41 | 4.18 | 4.08 | 0.042 | 1.783E+09 | 7 |
| **12** | 181.88 | 9.80 | 9.84 | 0.029 | 3.674E+09 | 7 |
| **13** | 167.23 | 10.17 | 10.14 | 0.034 | 8.173E+09 | 7 |
| **14** | 173.94 | 6.64 | 6.42 | 0.021 | 5.660E+09 | 7 |
| **15** | 169.87 | 5.21 | 4.97 | 0.016 | 6.453E+09 | 7 |
| **16** | 108.34 | 10.10 | 9.93 | 0.062 | 2.822E+09 | 7 |
| **17** | 121.29 | 8.15 | 8.21 | 0.044 | 2.234E+09 | 7 |
| **18** | 184.93 | 5.53 | 5.40 | 0.016 | 5.063E+09 | 7 |
| **19** | 105.46 | 8.34 | 8.20 | 0.053 | 2.272E+09 | 7 |
| **20** | 132.22 | 4.94 | 4.92 | 0.023 | 6.238E+09 | 7 |
| **21** | 95.92 | 4.23 | 4.23 | 0.031 | 3.297E+09 | 7 |

**Table S37.** $T_2$ relaxation times of **a$_3$CuMT2** (N40 G43S) binding **$Cu^+$**. The standard error for $T_2$ relaxation times was estimated using a Monte Carlo approach ($T_2$ MC SE) that combined the standard error for the $T_2$ fit ($T_2$ SE) and the standard error for fitting the initial intensity (M0 SE). The M0 SE is given for an intensity of one and is therefore a relative measure. The fitted absolute initial (delay = 0 ms) intensity (M0) is given in a separate column. The degrees of freedom (df) indicate the number of recorded data points (9 data points for a df of 7).

| | $T_2$ Relaxation [ms] | $T_2$ MC SE [ms] | $T_2$ SE [ms] | M0 SE [rel] | M0 [abs] | df |
|---|---|---|---|---|---|---|
| **1** | 155.68 | 4.57 | 4.73 | 0.018 | 8.421E+09 | 7 |
| **2** | 183.94 | 4.57 | 4.60 | 0.014 | 6.789E+09 | 7 |
| **3** | 97.01 | 14.49 | 13.46 | 0.098 | 1.214E+09 | 7 |
| **4** | 177.61 | 19.36 | 18.78 | 0.058 | 1.470E+09 | 7 |
| **5** | 159.43 | 7.15 | 6.84 | 0.025 | 1.528E+09 | 7 |
| **6** | 110.65 | 3.84 | 3.61 | 0.022 | 3.100E+09 | 7 |
| **7** | 128.90 | 8.84 | 8.97 | 0.044 | 1.106E+09 | 7 |
| **8** | 157.63 | 3.04 | 3.07 | 0.011 | 8.497E+09 | 7 |
| **9** | 124.82 | 4.84 | 4.77 | 0.025 | 5.731E+09 | 7 |
| **10** | 140.29 | 4.79 | 4.75 | 0.021 | 4.535E+09 | 7 |
| **11** | 179.74 | 7.84 | 7.60 | 0.023 | 6.887E+09 | 7 |
| **12** | 125.64 | 2.22 | 2.30 | 0.012 | 7.120E+09 | 7 |
| **13** | 182.04 | 9.38 | 9.56 | 0.029 | 1.490E+09 | 7 |
| **14** | 177.26 | 7.61 | 7.50 | 0.023 | 5.757E+09 | 7 |
| **15** | 132.00 | 4.33 | 4.28 | 0.020 | 5.681E+09 | 7 |
| **16** | 68.81 | 4.43 | 4.42 | 0.050 | 2.450E+09 | 7 |
| **17** | 107.76 | 6.38 | 6.56 | 0.041 | 3.900E+09 | 7 |
| **18** | 164.85 | 21.89 | 21.21 | 0.074 | 1.233E+09 | 7 |
| **19** | 165.35 | 5.75 | 5.85 | 0.020 | 2.269E+09 | 7 |
| **20** | 123.56 | 5.41 | 5.37 | 0.028 | 2.433E+09 | 7 |
| **21** | 90.99 | 6.01 | 5.96 | 0.047 | 3.498E+09 | 7 |
| **22** | 181.34 | 6.08 | 6.17 | 0.019 | 6.784E+09 | 7 |
| **23** | 136.88 | 5.31 | 5.25 | 0.024 | 6.818E+09 | 7 |
| **24** | 117.12 | 4.36 | 4.22 | 0.024 | 5.008E+09 | 7 |
| **25** | 108.26 | 10.33 | 10.53 | 0.066 | 1.194E+09 | 7 |
| **26** | 100.82 | 5.32 | 5.48 | 0.038 | 3.917E+09 | 7 |
| **27** | 172.66 | 6.90 | 7.08 | 0.023 | 9.043E+09 | 7 |
| **28** | 47.27 | 10.22 | 8.98 | 0.161 | 6.275E+08 | 7 |
| **29** | 144.55 | 15.39 | 14.73 | 0.062 | 1.650E+09 | 7 |

**Table S38.** $T_2$ relaxation times of **$a_4$CdMT** (N28) binding **$Cu^+$**. The standard error for $T_2$ relaxation times was estimated using a Monte Carlo approach ($T_2$ MC SE) that combined the standard error for the $T_2$ fit ($T_2$ SE) and the standard error for fitting the initial intensity (M0 SE). The M0 SE is given for an intensity of one and is therefore a relative measure. The fitted absolute initial (delay = 0 ms) intensity (M0) is given in a separate column. The degrees of freedom (df) indicate the number of recorded data points (9 data points for a df of 7).

| | $T_2$ Relaxation [ms] | $T_2$ MC SE [ms] | $T_2$ SE [ms] | M0 SE [rel] | M0 [abs] | df |
|---|---|---|---|---|---|---|
| **1** | 119.01 | 8.36 | 8.34 | 0.046 | 9.303E+08 | 7 |
| **2** | 114.66 | 10.24 | 10.57 | 0.061 | 1.087E+09 | 7 |
| **3** | 122.41 | 16.06 | 15.52 | 0.082 | 7.523E+08 | 7 |
| **4** | 142.28 | 19.05 | 17.75 | 0.076 | 9.321E+08 | 7 |
| **5** | 102.23 | 5.29 | 5.27 | 0.036 | 2.843E+09 | 7 |
| **6** | 115.32 | 21.00 | 18.41 | 0.106 | 7.884E+08 | 7 |
| **7** | 89.25 | 11.26 | 10.40 | 0.084 | 5.882E+08 | 7 |
| **8** | 95.51 | 12.46 | 11.46 | 0.085 | 1.491E+09 | 7 |
| **9** | 118.14 | 12.42 | 11.98 | 0.067 | 1.679E+09 | 7 |
| **10** | 146.95 | 11.55 | 10.90 | 0.045 | 2.205E+09 | 7 |
| **11** | 101.74 | 5.34 | 5.37 | 0.037 | 2.259E+09 | 7 |
| **12** | 168.51 | 18.67 | 17.78 | 0.060 | 8.019E+08 | 7 |
| **13** | 129.51 | 10.03 | 9.99 | 0.049 | 6.912E+08 | 7 |
| **14** | 151.06 | 18.61 | 17.55 | 0.069 | 1.298E+09 | 7 |
| **15** | 129.21 | 11.98 | 11.39 | 0.056 | 1.665E+09 | 7 |
| **16** | 132.99 | 17.97 | 17.81 | 0.084 | 7.522E+08 | 7 |
| **17** | 135.53 | 11.96 | 11.63 | 0.054 | 9.153E+08 | 7 |
| **18** | 195.26 | 22.92 | 22.31 | 0.060 | 1.021E+09 | 7 |
| **19** | 98.51 | 13.25 | 12.82 | 0.091 | 7.479E+08 | 7 |
| **20** | 202.62 | 17.90 | 17.66 | 0.045 | 2.176E+09 | 7 |
| **21** | 144.36 | 15.08 | 15.01 | 0.063 | 1.742E+09 | 7 |
| **22** | 108.37 | 12.68 | 12.22 | 0.077 | 1.662E+09 | 7 |
| **23** | 100.78 | 7.82 | 7.48 | 0.052 | 1.932E+09 | 7 |
| **24** | 161.31 | 14.54 | 13.76 | 0.049 | 2.629E+09 | 7 |
| **25** | 155.57 | 10.28 | 10.00 | 0.038 | 1.962E+09 | 7 |
| **26** | 172.44 | 14.46 | 14.32 | 0.046 | 1.209E+09 | 7 |
| **27** | 91.52 | 6.46 | 6.18 | 0.048 | 2.155E+09 | 7 |

**Table S39.** $T_2$ relaxation times of **$a_4$CuMT** (N46) binding **$Cu^+$**. The standard error for $T_2$ relaxation times was estimated using a Monte Carlo approach ($T_2$ MC SE) that combined the standard error for the $T_2$ fit ($T_2$ SE) and the standard error for fitting the initial intensity (M0 SE). The M0 SE is given for an intensity of one and is therefore a relative measure. The fitted absolute initial (delay = 0 ms) intensity (M0) is given in a separate column. The degrees of freedom (df) indicate the number of recorded data points (9 data points for a df of 7).

| | $T_2$ Relaxation [ms] | $T_2$ MC SE [ms] | $T_2$ SE [ms] | M0 SE [rel] | M0 [abs] | df |
|---|---|---|---|---|---|---|
| **1** | 224.32 | 14.72 | 14.95 | 0.032 | 3.509E+09 | 7 |
| **2** | 136.31 | 18.03 | 16.89 | 0.077 | 1.317E+09 | 7 |
| **3** | 178.85 | 9.15 | 8.96 | 0.028 | 2.723E+09 | 7 |
| **4** | 181.33 | 6.12 | 6.32 | 0.019 | 7.044E+09 | 7 |
| **5** | 182.31 | 18.63 | 17.78 | 0.053 | 1.851E+09 | 7 |
| **6** | 114.98 | 6.01 | 5.92 | 0.034 | 2.201E+09 | 7 |
| **7** | 170.24 | 16.45 | 16.51 | 0.055 | 2.304E+09 | 7 |
| **8** | 183.65 | 8.62 | 8.46 | 0.025 | 3.844E+09 | 7 |
| **9** | 122.47 | 4.18 | 4.27 | 0.023 | 9.550E+09 | 7 |
| **10** | 47.06 | 1.85 | 1.81 | 0.033 | 5.448E+09 | 7 |
| **11** | 37.82 | 10.58 | 8.48 | 0.200 | 1.736E+09 | 7 |
| **12** | 186.26 | 5.85 | 5.75 | 0.017 | 2.198E+09 | 7 |
| **13** | 154.61 | 4.73 | 4.91 | 0.019 | 1.087E+10 | 7 |
| **14** | 148.51 | 4.41 | 4.38 | 0.018 | 7.555E+09 | 7 |
| **15** | 135.11 | 2.37 | 2.39 | 0.011 | 2.024E+10 | 7 |
| **16** | 175.15 | 5.00 | 5.13 | 0.016 | 1.574E+10 | 7 |
| **17** | 142.41 | 4.87 | 4.84 | 0.021 | 8.123E+09 | 7 |
| **18** | 187.11 | 3.90 | 3.97 | 0.011 | 1.150E+10 | 7 |
| **19** | 174.57 | 4.59 | 4.64 | 0.015 | 1.790E+10 | 7 |
| **20** | 160.28 | 8.27 | 8.19 | 0.030 | 3.762E+09 | 7 |
| **21** | 134.26 | 7.24 | 7.45 | 0.035 | 3.023E+09 | 7 |
| **22** | 112.02 | 4.82 | 4.87 | 0.029 | 3.402E+09 | 7 |
| **23** | 43.43 | 6.88 | 6.64 | 0.132 | 1.510E+09 | 7 |
| **24** | 189.07 | 17.08 | 16.56 | 0.047 | 1.932E+10 | 7 |
| **25** | 107.67 | 3.71 | 3.69 | 0.023 | 1.113E+10 | 7 |
| **26** | 149.67 | 24.46 | 23.60 | 0.094 | 1.315E+09 | 7 |
| **27** | 128.09 | 5.51 | 5.54 | 0.028 | 9.833E+09 | 7 |
| **28** | 143.91 | 7.16 | 7.12 | 0.030 | 3.729E+09 | 7 |
| **29** | 65.58 | 3.59 | 3.55 | 0.043 | 3.155E+09 | 7 |
| **30** | 183.09 | 12.23 | 12.09 | 0.036 | 3.151E+09 | 7 |
| **31** | 113.52 | 10.94 | 10.89 | 0.064 | 2.011E+09 | 7 |

**Table S40.** $T_2$ relaxation times of **$a_4$UnMT** (N41) binding **$Cu^+$**. The standard error for $T_2$ relaxation times was estimated using a Monte Carlo approach ($T_2$ MC SE) that combined the standard error for the $T_2$ fit ($T_2$ SE) and the standard error for fitting the initial intensity (M0 SE). The M0 SE is given for an intensity of one and is therefore a relative measure. The fitted absolute initial (delay = 0 ms) intensity (M0) is given in a separate column. The degrees of freedom (df) indicate the number of recorded data points (9 data points for a df of 7).

| | $T_2$ Relaxation [ms] | $T_2$ MC SE [ms] | $T_2$ SE [ms] | M0 SE [rel] | M0 [abs] | df |
|---|---|---|---|---|---|---|
| **1** | 164.07 | 6.08 | 6.01 | 0.021 | 5.180E+09 | 7 |
| **2** | 177.00 | 7.21 | 6.85 | 0.021 | 6.320E+09 | 7 |
| **3** | 132.03 | 7.67 | 7.51 | 0.036 | 4.297E+09 | 7 |
| **4** | 80.31 | 4.81 | 4.66 | 0.043 | 1.767E+09 | 7 |
| **5** | 183.54 | 6.80 | 6.67 | 0.020 | 3.641E+09 | 7 |
| **6** | 216.39 | 18.08 | 18.76 | 0.043 | 2.554E+09 | 7 |
| **7** | 174.82 | 7.04 | 6.86 | 0.022 | 4.024E+09 | 7 |
| **8** | 145.57 | 5.57 | 5.57 | 0.023 | 2.474E+09 | 7 |
| **9** | 97.41 | 7.84 | 7.49 | 0.054 | 1.838E+09 | 7 |
| **10** | 105.60 | 4.18 | 4.17 | 0.027 | 5.389E+09 | 7 |
| **11** | 155.09 | 4.49 | 4.59 | 0.017 | 5.838E+09 | 7 |
| **12** | 154.31 | 11.35 | 11.28 | 0.043 | 2.642E+09 | 7 |
| **13** | 178.02 | 11.67 | 11.72 | 0.036 | 3.600E+09 | 7 |
| **14** | 173.23 | 5.29 | 5.39 | 0.017 | 5.187E+09 | 7 |
| **15** | 124.29 | 7.48 | 7.78 | 0.040 | 5.713E+09 | 7 |
| **16** | 173.98 | 4.39 | 4.36 | 0.014 | 9.291E+09 | 7 |
| **17** | 151.55 | 4.32 | 4.40 | 0.017 | 7.227E+09 | 7 |
| **18** | 113.83 | 6.92 | 6.92 | 0.041 | 3.541E+09 | 7 |
| **19** | 194.15 | 22.13 | 21.04 | 0.057 | 2.185E+09 | 7 |
| **20** | 118.77 | 7.88 | 7.47 | 0.041 | 2.193E+09 | 7 |
| **21** | 66.11 | 13.88 | 12.59 | 0.149 | 8.386E+08 | 7 |
| **22** | 175.81 | 6.59 | 6.69 | 0.021 | 8.342E+09 | 7 |
| **23** | 55.93 | 4.01 | 4.05 | 0.059 | 2.166E+09 | 7 |
| **24** | 169.89 | 14.11 | 13.55 | 0.045 | 2.596E+09 | 7 |
| **25** | 155.39 | 10.13 | 10.03 | 0.038 | 2.128E+09 | 7 |
| **26** | 116.91 | 9.00 | 8.64 | 0.049 | 1.363E+09 | 7 |
| **27** | 141.48 | 11.43 | 11.28 | 0.049 | 3.862E+09 | 7 |
| **28** | 38.87 | 3.70 | 3.74 | 0.085 | 1.406E+09 | 7 |
| **29** | 126.66 | 3.85 | 3.91 | 0.020 | 3.167E+09 | 7 |
| **30** | 185.73 | 5.71 | 5.95 | 0.017 | 4.216E+09 | 7 |
| **31** | 149.60 | 8.12 | 8.50 | 0.034 | 4.756E+09 | 7 |
| **32** | 142.77 | 8.67 | 8.66 | 0.037 | 2.579E+09 | 7 |
| **33** | 129.66 | 5.88 | 5.74 | 0.028 | 2.995E+09 | 7 |
| **34** | 162.60 | 19.63 | 18.39 | 0.065 | 1.164E+09 | 7 |
| **35** | 168.43 | 4.23 | 4.17 | 0.014 | 3.646E+09 | 7 |
| **36** | 148.28 | 8.04 | 7.96 | 0.032 | 2.905E+09 | 7 |
| **37** | 143.47 | 10.33 | 10.05 | 0.043 | 2.298E+09 | 7 |
| **38** | 196.60 | 17.16 | 17.09 | 0.045 | 3.454E+09 | 7 |
| **39** | 87.50 | 7.38 | 7.29 | 0.061 | 2.243E+09 | 7 |
| **40** | 141.28 | 9.11 | 9.21 | 0.040 | 1.790E+09 | 7 |
| **41** | 165.85 | 7.88 | 8.05 | 0.028 | 5.999E+09 | 7 |
| **42** | 179.82 | 8.86 | 8.82 | 0.027 | 4.769E+09 | 7 |
| **43** | 171.81 | 3.94 | 3.81 | 0.012 | 3.489E+09 | 7 |
| **44** | 186.89 | 19.66 | 19.25 | 0.055 | 1.227E+09 | 7 |
| **45** | 212.16 | 10.75 | 10.65 | 0.025 | 3.057E+09 | 7 |
| **46** | 131.92 | 19.50 | 17.91 | 0.086 | 1.526E+09 | 7 |
| **47** | 95.40 | 2.61 | 2.63 | 0.019 | 3.295E+09 | 7 |
| **48** | 159.51 | 4.33 | 4.18 | 0.015 | 3.771E+09 | 7 |
| **49** | 177.34 | 11.35 | 10.95 | 0.034 | 1.745E+09 | 7 |
| **50** | 214.27 | 12.69 | 12.57 | 0.029 | 2.576E+09 | 7 |

**Table S41.** $T_2$ relaxation times of **HpCdMT** binding **$Cu^+$**. The standard error for $T_2$ relaxation times was estimated using a Monte Carlo approach ($T_2$ MC SE) that combined the standard error for the $T_2$ fit ($T_2$ SE) and the standard error for fitting the initial intensity (M0 SE). The M0 SE is given for an intensity of one and is therefore a relative measure. The fitted absolute initial (delay = 0 ms) intensity (M0) is given in a separate column. The degrees of freedom (df) indicate the number of recorded data points (9 data points for a df of 7).

| | $T_2$ Relaxation [ms] | $T_2$ MC SE [ms] | $T_2$ SE [ms] | M0 SE [rel] | M0 [abs] | df |
|---|---|---|---|---|---|---|
| **1** | 74.71 | 8.83 | 8.49 | 0.086 | 5.392E+08 | 7 |
| **2** | 137.70 | 8.14 | 8.12 | 0.037 | 1.226E+09 | 7 |
| **3** | 131.38 | 17.62 | 16.29 | 0.078 | 8.219E+08 | 7 |
| **4** | 84.27 | 8.78 | 8.79 | 0.077 | 7.380E+08 | 7 |
| **5** | 146.80 | 8.61 | 8.18 | 0.034 | 1.641E+09 | 7 |
| **6** | 95.30 | 7.77 | 7.75 | 0.058 | 9.407E+08 | 7 |
| **7** | 134.18 | 4.60 | 4.69 | 0.022 | 1.975E+09 | 7 |
| **8** | 106.68 | 7.73 | 7.55 | 0.048 | 1.297E+09 | 7 |
| **9** | 183.27 | 11.79 | 11.51 | 0.034 | 2.044E+09 | 7 |
| **10** | 143.28 | 5.56 | 5.59 | 0.024 | 2.903E+09 | 7 |
| **11** | 137.97 | 16.70 | 16.08 | 0.072 | 7.949E+08 | 7 |
| **12** | 104.43 | 13.06 | 13.02 | 0.086 | 8.059E+08 | 7 |
| **13** | 199.41 | 8.67 | 8.67 | 0.023 | 1.515E+09 | 7 |
| **14** | 126.66 | 22.91 | 22.30 | 0.113 | 5.215E+08 | 7 |
| **15** | 92.58 | 13.21 | 12.70 | 0.098 | 6.898E+08 | 7 |
| **16** | 147.27 | 10.04 | 9.71 | 0.040 | 1.223E+09 | 7 |
| **17** | 132.05 | 9.36 | 9.26 | 0.044 | 1.071E+09 | 7 |
| **18** | 60.45 | 8.07 | 7.38 | 0.098 | 5.974E+08 | 7 |
| **19** | 122.56 | 12.96 | 12.21 | 0.065 | 1.115E+09 | 7 |
| **20** | 133.48 | 8.43 | 8.11 | 0.038 | 2.221E+09 | 7 |
| **21** | 139.47 | 11.94 | 11.84 | 0.052 | 8.544E+08 | 7 |
| **22** | 103.30 | 9.06 | 8.47 | 0.057 | 1.498E+09 | 7 |
| **23** | 163.70 | 11.40 | 11.09 | 0.039 | 1.567E+09 | 7 |
| **24** | 122.59 | 8.64 | 8.48 | 0.045 | 1.130E+09 | 7 |
| **25** | 143.41 | 5.22 | 5.42 | 0.023 | 2.824E+09 | 7 |
| **26** | 80.96 | 9.60 | 9.48 | 0.087 | 6.326E+08 | 7 |
| **27** | 101.54 | 16.64 | 15.59 | 0.107 | 6.367E+08 | 7 |
| **28** | 125.32 | 14.23 | 14.06 | 0.072 | 9.304E+08 | 7 |

**Table S42.** $T_2$ relaxation times of **HpCuMT** binding **$Cu^+$**. The standard error for $T_2$ relaxation times was estimated using a Monte Carlo approach ($T_2$ MC SE) that combined the standard error for the $T_2$ fit ($T_2$ SE) and the standard error for fitting the initial intensity (M0 SE). The M0 SE is given for an intensity of one and is therefore a relative measure. The fitted absolute initial (delay = 0 ms) intensity (M0) is given in a separate column. The degrees of freedom (df) indicate the number of recorded data points (9 data points for a df of 7).

| | $T_2$ Relaxation [ms] | $T_2$ MC SE [ms] | $T_2$ SE [ms] | M0 SE [rel] | M0 [abs] | df |
|---|---|---|---|---|---|---|
| **1** | 103.47 | 7.07 | 7.21 | 0.048 | 2.986E+09 | 7 |
| **2** | 96.10 | 4.26 | 4.29 | 0.032 | 4.762E+09 | 7 |
| **3** | 70.78 | 4.08 | 4.02 | 0.044 | 5.161E+09 | 7 |
| **4** | 83.04 | 4.78 | 4.84 | 0.043 | 6.243E+09 | 7 |
| **5** | 111.99 | 4.73 | 4.82 | 0.029 | 6.103E+09 | 7 |
| **6** | 130.72 | 4.46 | 4.33 | 0.021 | 4.657E+09 | 7 |
| **7** | 129.87 | 4.07 | 4.17 | 0.020 | 8.225E+09 | 7 |
| **8** | 92.98 | 6.86 | 6.47 | 0.050 | 6.238E+09 | 7 |
| **9** | 102.37 | 5.20 | 5.26 | 0.036 | 4.599E+09 | 7 |
| **10** | 142.39 | 3.27 | 3.28 | 0.014 | 8.953E+09 | 7 |
| **11** | 133.45 | 3.40 | 3.32 | 0.016 | 8.596E+09 | 7 |
| **12** | 133.22 | 7.02 | 6.86 | 0.032 | 3.011E+09 | 7 |
| **13** | 80.53 | 3.40 | 3.42 | 0.032 | 4.740E+09 | 7 |
| **14** | 164.46 | 17.26 | 16.20 | 0.056 | 1.320E+09 | 7 |
| **15** | 107.43 | 4.78 | 4.69 | 0.030 | 6.254E+09 | 7 |
| **16** | 118.68 | 4.45 | 4.42 | 0.024 | 8.867E+09 | 7 |
| **17** | 153.31 | 6.94 | 6.63 | 0.026 | 1.162E+10 | 7 |
| **18** | 149.19 | 8.09 | 7.91 | 0.032 | 6.285E+09 | 7 |
| **19** | 139.32 | 5.94 | 6.03 | 0.027 | 6.615E+09 | 7 |
| **20** | 141.20 | 5.57 | 5.48 | 0.024 | 5.675E+09 | 7 |
| **21** | 116.29 | 3.91 | 3.63 | 0.021 | 5.283E+09 | 7 |
| **22** | 155.53 | 4.98 | 4.82 | 0.018 | 7.279E+09 | 7 |
| **23** | 134.36 | 4.14 | 4.07 | 0.019 | 5.282E+09 | 7 |
| **24** | 76.97 | 3.05 | 2.91 | 0.029 | 5.977E+09 | 7 |
| **25** | 172.72 | 2.92 | 2.80 | 0.009 | 1.342E+10 | 7 |
| **26** | 159.05 | 5.75 | 5.74 | 0.021 | 8.632E+09 | 7 |
| **27** | 123.44 | 9.54 | 9.25 | 0.049 | 4.679E+09 | 7 |
| **28** | 92.03 | 4.24 | 4.01 | 0.031 | 5.011E+09 | 7 |
| **29** | 93.08 | 2.96 | 2.92 | 0.022 | 5.185E+09 | 7 |
| **30** | 30.78 | 5.41 | 4.98 | 0.152 | 2.498E+09 | 7 |
| **31** | 52.30 | 5.41 | 5.41 | 0.086 | 3.488E+09 | 7 |
| **32** | 163.65 | 6.55 | 6.45 | 0.023 | 5.810E+09 | 7 |
| **33** | 160.83 | 6.49 | 6.43 | 0.023 | 3.971E+09 | 7 |
| **34** | 74.09 | 3.32 | 3.32 | 0.034 | 3.810E+09 | 7 |
| **35** | 107.05 | 5.86 | 5.72 | 0.036 | 7.007E+09 | 7 |
| **36** | 142.51 | 5.80 | 5.94 | 0.025 | 5.647E+09 | 7 |
| **37** | 98.19 | 8.53 | 8.33 | 0.060 | 1.901E+09 | 7 |
| **38** | 135.58 | 2.34 | 2.36 | 0.011 | 5.640E+09 | 7 |
| **39** | 142.46 | 5.91 | 5.61 | 0.024 | 7.355E+09 | 7 |
| **40** | 110.06 | 5.32 | 5.24 | 0.032 | 3.063E+09 | 7 |
| **41** | 134.01 | 4.62 | 4.45 | 0.021 | 5.117E+09 | 7 |
| **42** | 147.27 | 4.70 | 4.67 | 0.019 | 1.031E+10 | 7 |
| **43** | 114.94 | 5.19 | 5.35 | 0.031 | 4.129E+09 | 7 |
| **44** | 71.70 | 2.26 | 2.30 | 0.025 | 5.833E+09 | 7 |
| **45** | 145.01 | 5.60 | 5.76 | 0.024 | 6.115E+09 | 7 |
| **46** | 109.48 | 6.77 | 6.23 | 0.039 | 4.427E+09 | 7 |
| **47** | 98.99 | 8.94 | 8.38 | 0.059 | 2.955E+09 | 7 |
| **48** | 122.37 | 10.57 | 10.56 | 0.056 | 1.574E+09 | 7 |
| **49** | 116.83 | 8.03 | 7.81 | 0.044 | 5.158E+09 | 7 |
| **50** | 142.48 | 5.88 | 5.97 | 0.026 | 1.068E+10 | 7 |
| **51** | 83.98 | 14.27 | 13.31 | 0.117 | 1.457E+09 | 7 |
| **52** | 98.89 | 5.19 | 5.27 | 0.037 | 3.973E+09 | 7 |
| **53** | 114.42 | 4.82 | 4.76 | 0.028 | 4.019E+09 | 7 |

**Table S43.** $T_2$ relaxation times of **HpUnMT1** binding **$Cu^+$**. The standard error for $T_2$ relaxation times was estimated using a Monte Carlo approach ($T_2$ MC SE) that combined the standard error for the $T_2$ fit ($T_2$ SE) and the standard error for fitting the initial intensity (M0 SE). The M0 SE is given for an intensity of one and is therefore a relative measure. The fitted absolute initial (delay = 0 ms) intensity (M0) is given in a separate column. The degrees of freedom (df) indicate the number of recorded data points (9 data points for a df of 7).

| | $T_2$ Relaxation [ms] | $T_2$ MC SE [ms] | $T_2$ SE [ms] | M0 SE [rel] | M0 [abs] | df |
|---|---|---|---|---|---|---|
| **1** | 92.00 | 8.48 | 8.24 | 0.064 | 7.060E+09 | 7 |
| **2** | 83.17 | 4.06 | 3.96 | 0.035 | 1.066E+10 | 7 |
| **3** | 51.65 | 2.24 | 2.11 | 0.034 | 5.168E+09 | 7 |
| **4** | 183.27 | 6.38 | 6.38 | 0.019 | 1.993E+10 | 7 |
| **5** | 217.13 | 7.36 | 7.21 | 0.016 | 1.097E+10 | 7 |
| **6** | 151.26 | 3.94 | 3.67 | 0.014 | 1.302E+10 | 7 |
| **7** | 153.72 | 3.67 | 3.78 | 0.015 | 1.812E+10 | 7 |
| **8** | 224.38 | 6.25 | 6.20 | 0.013 | 1.413E+10 | 7 |
| **9** | 165.62 | 6.95 | 6.99 | 0.024 | 1.314E+10 | 7 |
| **10** | 137.71 | 3.94 | 4.03 | 0.018 | 1.383E+10 | 7 |
| **11** | 105.54 | 7.19 | 6.91 | 0.045 | 7.554E+09 | 7 |
| **12** | 135.29 | 14.79 | 13.87 | 0.064 | 5.336E+09 | 7 |
| **13** | 104.86 | 6.95 | 6.93 | 0.045 | 5.363E+09 | 7 |
| **14** | 116.62 | 11.71 | 11.19 | 0.063 | 1.021E+10 | 7 |
| **15** | 109.90 | 2.60 | 2.62 | 0.016 | 1.151E+10 | 7 |
| **16** | 81.38 | 4.27 | 4.26 | 0.039 | 5.619E+09 | 7 |
| **17** | 181.89 | 5.39 | 5.61 | 0.017 | 1.893E+10 | 7 |
| **18** | 166.86 | 3.73 | 3.65 | 0.012 | 1.606E+10 | 7 |
| **19** | 200.36 | 7.08 | 7.19 | 0.019 | 1.705E+10 | 7 |
| **20** | 91.85 | 7.07 | 6.90 | 0.054 | 7.087E+09 | 7 |
| **21** | 83.01 | 3.81 | 3.77 | 0.034 | 1.181E+10 | 7 |
| **22** | 107.27 | 3.11 | 3.25 | 0.021 | 1.154E+10 | 7 |
| **23** | 173.82 | 5.64 | 5.78 | 0.019 | 1.744E+10 | 7 |
| **24** | 158.13 | 3.11 | 3.15 | 0.012 | 1.345E+10 | 7 |
| **25** | 159.64 | 11.08 | 10.71 | 0.039 | 4.658E+09 | 7 |
| **26** | 170.66 | 6.75 | 6.74 | 0.022 | 1.629E+10 | 7 |
| **27** | 181.90 | 6.13 | 6.11 | 0.018 | 1.533E+10 | 7 |
| **28** | 158.49 | 4.61 | 4.76 | 0.017 | 1.234E+10 | 7 |
| **29** | 184.77 | 6.12 | 6.07 | 0.018 | 1.067E+10 | 7 |
| **30** | 187.63 | 6.36 | 6.24 | 0.018 | 2.062E+10 | 7 |
| **31** | 135.15 | 18.25 | 17.16 | 0.079 | 3.550E+09 | 7 |
| **32** | 165.96 | 7.81 | 7.70 | 0.026 | 1.415E+10 | 7 |
| **33** | 76.79 | 3.07 | 3.14 | 0.031 | 6.452E+09 | 7 |
| **34** | 194.40 | 4.09 | 4.15 | 0.011 | 1.159E+10 | 7 |
| **35** | 129.37 | 9.98 | 9.68 | 0.048 | 1.111E+10 | 7 |
| **36** | 132.86 | 4.33 | 4.38 | 0.021 | 8.689E+09 | 7 |
| **37** | 128.68 | 5.49 | 5.56 | 0.028 | 1.455E+10 | 7 |
| **38** | 104.68 | 3.68 | 3.62 | 0.024 | 9.211E+09 | 7 |
| **39** | 134.66 | 5.05 | 4.85 | 0.023 | 1.241E+10 | 7 |
| **40** | 83.66 | 3.53 | 3.66 | 0.032 | 9.441E+09 | 7 |
| **41** | 123.50 | 3.69 | 3.82 | 0.020 | 1.175E+10 | 7 |
| **42** | 130.46 | 5.24 | 5.17 | 0.025 | 1.085E+10 | 7 |
| **43** | 123.23 | 9.53 | 9.04 | 0.048 | 9.095E+09 | 7 |
| **44** | 144.55 | 7.63 | 7.88 | 0.033 | 1.618E+10 | 7 |
| **45** | 167.88 | 6.03 | 6.04 | 0.020 | 1.603E+10 | 7 |
| **46** | 129.14 | 9.53 | 9.05 | 0.045 | 1.244E+10 | 7 |
| **47** | 132.83 | 6.47 | 6.28 | 0.030 | 1.926E+10 | 7 |
| **48** | 143.78 | 5.93 | 6.08 | 0.026 | 1.846E+10 | 7 |
| **49** | 145.28 | 4.06 | 4.00 | 0.017 | 8.822E+09 | 7 |
| **50** | 112.45 | 5.32 | 5.41 | 0.032 | 1.545E+10 | 7 |
| **51** | 111.44 | 11.38 | 10.66 | 0.064 | 7.131E+09 | 7 |
| **52** | 135.84 | 14.25 | 13.19 | 0.061 | 6.733E+09 | 7 |

**Table S44.** $T_2$ relaxation times of **HpUnMT2** binding **$Cu^+$**. The standard error for $T_2$ relaxation times was estimated using a Monte Carlo approach ($T_2$ MC SE) that combined the standard error for the $T_2$ fit ($T_2$ SE) and the standard error for fitting the initial intensity (M0 SE). The M0 SE is given for an intensity of one and is therefore a relative measure. The fitted absolute initial (delay = 0 ms) intensity (M0) is given in a separate column. The degrees of freedom (df) indicate the number of recorded data points (9 data points for a df of 7).

| | $T_2$ Relaxation [ms] | $T_2$ MC SE [ms] | $T_2$ SE [ms] | M0 SE [rel] | M0 [abs] | df |
|---|---|---|---|---|---|---|
| **1** | 159.87 | 5.07 | 4.94 | 0.018 | 1.010E+10 | 7 |
| **2** | 229.09 | 13.53 | 13.89 | 0.029 | 1.462E+09 | 7 |
| **3** | 99.35 | 3.11 | 3.13 | 0.022 | 8.416E+09 | 7 |
| **4** | 162.87 | 4.11 | 4.07 | 0.014 | 1.339E+10 | 7 |
| **5** | 155.44 | 6.53 | 6.60 | 0.025 | 1.330E+10 | 7 |
| **6** | 168.83 | 5.25 | 5.23 | 0.018 | 1.139E+10 | 7 |
| **7** | 188.69 | 6.46 | 6.46 | 0.018 | 1.094E+10 | 7 |
| **8** | 126.92 | 4.56 | 4.58 | 0.023 | 1.081E+10 | 7 |
| **9** | 182.49 | 11.10 | 10.65 | 0.032 | 1.275E+10 | 7 |
| **10** | 156.36 | 4.65 | 4.53 | 0.017 | 1.168E+10 | 7 |
| **11** | 111.51 | 4.63 | 4.84 | 0.029 | 1.037E+10 | 7 |
| **12** | 124.98 | 5.24 | 5.26 | 0.027 | 8.704E+09 | 7 |
| **13** | 158.48 | 4.14 | 4.12 | 0.015 | 1.233E+10 | 7 |
| **14** | 179.52 | 4.42 | 4.43 | 0.014 | 1.515E+10 | 7 |
| **15** | 145.95 | 5.83 | 5.66 | 0.023 | 7.345E+09 | 7 |
| **16** | 162.80 | 5.18 | 5.09 | 0.018 | 8.992E+09 | 7 |
| **17** | 183.46 | 15.70 | 14.84 | 0.044 | 2.302E+09 | 7 |
| **18** | 201.67 | 5.32 | 5.15 | 0.013 | 8.395E+09 | 7 |
| **19** | 116.72 | 5.89 | 5.94 | 0.034 | 8.820E+09 | 7 |
| **20** | 177.69 | 6.69 | 6.51 | 0.020 | 1.152E+10 | 7 |
| **21** | 193.40 | 4.41 | 4.39 | 0.012 | 1.431E+10 | 7 |
| **22** | 186.40 | 4.94 | 5.00 | 0.014 | 1.304E+10 | 7 |
| **23** | 135.88 | 3.97 | 3.89 | 0.018 | 1.176E+10 | 7 |
| **24** | 150.31 | 3.46 | 3.36 | 0.013 | 8.022E+09 | 7 |
| **25** | 113.20 | 2.78 | 2.80 | 0.017 | 6.720E+09 | 7 |
| **26** | 149.69 | 3.95 | 3.88 | 0.016 | 8.750E+09 | 7 |
| **27** | 96.21 | 2.35 | 2.35 | 0.017 | 5.583E+09 | 7 |
| **28** | 53.66 | 2.63 | 2.56 | 0.039 | 4.265E+09 | 7 |
| **29** | 113.56 | 3.81 | 3.84 | 0.023 | 1.318E+10 | 7 |
| **30** | 100.18 | 4.02 | 4.03 | 0.028 | 9.639E+09 | 7 |
| **31** | 162.32 | 4.05 | 4.03 | 0.014 | 1.084E+10 | 7 |
| **32** | 97.08 | 2.44 | 2.41 | 0.017 | 5.336E+09 | 7 |
| **33** | 162.17 | 7.56 | 7.50 | 0.027 | 1.249E+10 | 7 |
| **34** | 197.32 | 5.65 | 5.64 | 0.015 | 1.355E+10 | 7 |
| **35** | 144.51 | 6.18 | 6.17 | 0.026 | 4.993E+09 | 7 |
| **36** | 175.87 | 3.54 | 3.57 | 0.011 | 1.096E+10 | 7 |
| **37** | 154.80 | 4.32 | 4.36 | 0.017 | 3.987E+09 | 7 |
| **38** | 214.87 | 5.57 | 5.59 | 0.013 | 9.992E+09 | 7 |
| **39** | 158.95 | 6.95 | 6.91 | 0.025 | 6.357E+09 | 7 |
| **40** | 179.89 | 4.51 | 4.39 | 0.013 | 1.380E+10 | 7 |
| **41** | 154.93 | 4.38 | 4.18 | 0.016 | 6.584E+09 | 7 |
| **42** | 106.44 | 3.35 | 3.40 | 0.022 | 7.766E+09 | 7 |
| **43** | 153.20 | 4.13 | 4.04 | 0.016 | 6.403E+09 | 7 |
| **44** | 140.80 | 5.57 | 5.79 | 0.025 | 6.757E+09 | 7 |
| **45** | 157.73 | 10.49 | 10.30 | 0.038 | 3.672E+09 | 7 |
| **46** | 192.24 | 3.45 | 3.37 | 0.009 | 1.001E+10 | 7 |
| **47** | 111.32 | 3.47 | 3.58 | 0.022 | 4.965E+09 | 7 |
| **48** | 148.45 | 4.56 | 4.66 | 0.019 | 1.263E+10 | 7 |
| **49** | 103.48 | 8.26 | 7.99 | 0.053 | 1.313E+09 | 7 |
| **50** | 124.59 | 3.83 | 3.85 | 0.020 | 1.087E+10 | 7 |
| **51** | 199.32 | 6.37 | 6.22 | 0.016 | 1.014E+10 | 7 |
| **52** | 155.66 | 4.33 | 4.41 | 0.017 | 1.110E+10 | 7 |
| **53** | 135.48 | 7.75 | 7.51 | 0.035 | 5.418E+09 | 7 |
